# A new African Titanosaurian Sauropod Dinosaur from the middle Cretaceous Galula Formation (Mtuka Member), Rukwa Rift Basin, Southwestern Tanzania

**DOI:** 10.1371/journal.pone.0211412

**Published:** 2019-02-13

**Authors:** Eric Gorscak, Patrick M. O’Connor

**Affiliations:** 1 Department of Anatomy, Midwestern University, Downers Grove, Illinois, United States of America; 2 Integrative Research Center, Field Museum of Natural History, Chicago, Illinois, United States of America; 3 Department of Biological Sciences, Ohio University, Athens, Ohio, United States of America; 4 Department of Biomedical Sciences, Ohio University Heritage College of Osteopathic Medicine, Athens, Ohio, United States of America; 5 Ohio Center for Ecology and Evolutionary Studies, Ohio University, Athens, Ohio, United States of America; State Museum of Natural History, GERMANY

## Abstract

The African terrestrial fossil record has been limited in its contribution to our understanding of both regional and global Cretaceous paleobiogeography, an interval of significant geologic and macroevolutionary change. A common component in Cretaceous African faunas, titanosaurian sauropods diversified into one of the most specious groups of dinosaurs worldwide. Here we describe the new titanosaurian *Mnyamawamtuka moyowamkia* gen. et sp. nov. from the Mtuka Member of the Galula Formation in southwest Tanzania. The new specimen preserves teeth, elements from all regions of the postcranial axial skeleton, parts of both appendicular girdles, and portions of both limbs including a complete metatarsus. Unique traits of *M*. *moyowamkia* include the lack of an interpostzygapophyseal lamina in posterior dorsal vertebrae, pronounced posterolateral expansion of middle caudal centra, and an unusually small sternal plate. Phylogenetic analyses consistently place *M*. *moyowamkia* as either a close relative to lithostrotian titanosaurians (e.g., parsimony, uncalibrated Bayesian analyses) or as a lithostrotian and sister taxon to *Malawisaurus dixeyi* from the nearby Aptian? Dinosaur Beds of Malawi (e.g., tip-dating Bayesian analyses). *M*. *moyowamkia* shares a few features with *M*. *dixeyi*, including semi-spatulate teeth and a median lamina between the neural canal and interpostzygapophyseal lamina in anterior dorsal vertebrae. Both comparative morphology and phylogenetic analyses support *Mnyamawamtuka* as a distinct and distant relative to *Rukwatitan bisepultus* and *Shingopana songwensis* from the younger Namba Member of the Galula Formation with these results largely congruent with newly constrained ages for the Mtuka Member (Aptian–Cenomanian) and Namba Member (Campanian). Coupled with recent discoveries from the Dahkla Oasis, Egypt (e.g., *Mansourasaurus shahinae*) and other parts of continental Afro-Arabia, the Tanzania titanosaurians refine perspectives on the development of African terrestrial faunas throughout the Cretaceous—a critical step in understanding non-marine paleobiogeographic patterns of Africa that have remained elusive until the past few years.

## Introduction

The Cretaceous fossil record of Afro-Arabia remains an active area of research from both regional and global paleobiogeographic perspectives [1–16]. Despite this handful of recent discoveries, extensive work is still required to adequately characterize and constrain depositional units in concert with the recovery of diagnostic fossils (i.e., identifiable to the genus-species level and evaluated in a robust macroevolutionary context) in order to properly assess the paleobiogeographic role of continental Afro-Arabia and its faunas throughout the Cretaceous Period. One group of terrestrial organisms that may provide a clearer perspective on the Cretaceous paleobiogeography of Africa are the titanosaurian sauropod dinosaurs [12,16,17]. Titanosaurians were one of the most speciose and globally distributed groups of dinosaurs during the Cretaceous period [18–23]. Importantly, other major sauropod groups (e.g., rebbachisaurids, dicraeosaurids, euhelopodids, brachiosaurids) persisted alongside early titanosaurians from the Early Cretaceous until all non-titanosaurian sauropods finally succumbed to extinction by the early Late Cretaceous with their fossil remains recovered from North and South America, northern Africa, Europe, and Asia [9, 20, 22, 24–30]. With many different sauropod clades present globally during the Early Cretaceous, some of which clearly represent groups with a Jurassic origin, the origins of titanosaurians remain elusive among the diverse assemblage of sauropod clades known from around the world [17, 29, 31–33].

Currently, the earliest unambiguous titanosaurian body fossils are known from Barremian units on the Isle of Wright, United Kingdom (NHMUK 5333, procoelous middle caudal vertebrae), whereas the geologically oldest unambiguously named titanosaurian taxa are from the slightly younger Aptian: *Malawisaurus dixeyi*, Malawi [34], *Karongasaurus gittelmani*, Malawi [3], and *Tapuiasaurus macedoi*, Brazil [35]. *T*. *macedoi* preserves a nearly complete skull and multiple elements from the postcranium, but awaits full description [35, 36]. *M*. *dixeyi* is currently the best-known titanosaurian from Africa, represented by both cranial and post-cranial materials collected from several localities [3, 34, 37], whereas the other Malawian titanosaurian, *K*. *gittelmani*, is known only from a partial dentary and several referred teeth [3]. Other proposed titanosaurian fossils from older deposits are both much more incomplete and/or ambiguous from character distribution perspectives. Mannion et al. [33] recovered the Late Jurassic *Australodocus bohetii* from Tanzania as a titanosaurian, however, the taxon was removed in some analyses due to its instability. *A*. *bohetii* is overwhelmingly incomplete, as the holotype consists of a single partial middle cervical vertebra with another partial middle cervical vertebra as a referred specimen [38]. In previous studies, *A*. *bohetii* has been recovered as a diplodocid [38], an indeterminate macronarian [28], or indeterminate titanosauriform [29, 39], and is here regarded as ambiguous in its affinities until more informative fossils are recovered. Other recently described titanosaurians from Lower Cretaceous units are, unfortunately, largely incomplete: *Triunfosaurus leonardii* from the Rio Piranhas Formation (Berriasian–Hauterivian) in Brazil [40], and *Tengrisaurus starkovi* from the Murtoi Formation (Barremian–Aptian) in Russia [41]. A partial middle caudal vertebra and two unidentified elements recovered from the Aptian–Albian of Italy have been proposed to be of lithostrotian affinities [42], and more specifically, to be linked with the Afro-Malagasy titanosaurians *M*. *dixeyi* (Aptian of Malawi) and *Rapetosaurus krausei* (Maastrichtian of Madagascar). These recent titanosaurian discoveries are encouraging, regardless of completeness, as they support an Early Cretaceous origin for titanosaurians during this poorly sampled interval [17, 29].

The evolutionary history of Titanosauria is best represented in South America, the continent that has yielded the vast majority of known species. By contrast, the African-side of their evolutionary history is steadily gaining recognition with recently established taxa *Rukwatitan bisepultus* [11], *Shingopana songwensis* [12], and *Mansourasaurus shahinae* [16], alongside other notable and/or potential African titanosaurians (e.g., *Angolatitan adamastor*, *K*. *gittelmani*, *M*. *dixeyi*, *Paralititan stromeri*). Initially, Gorscak et al. [11] recovered *R*. *bisepultus* as a non-lithostrotian titanosaurian, but subsequent studies have regularly recovered *R*. *bisepultus* as a Lithostrotian titanosaurian either as the sister taxon to *M*. *dixeyi* [43], or as a member of the saltasaur-lineage within Lithostrotia [12, 17]. Moreover, *R*. *bisepultus* was recovered from the younger Namba Member of the Galula Formation of Tanzania along with all of the currently named taxa from the Galula Formation, such as the aeolosaurine-related titanosaurian *S*. *songwensis* [12], and the crocodyliforms *Pakasuchus kapilimai* (Notosuchia; [5]) and *Rukwasuchus yajabalijekundu* (Peirosauridae; [13]). Yet, the stratigraphically lower Mtuka Member is comparatively less known with recovered fossils representing osteichthyian fish, turtles, and indeterminate theropod and sauropod dinosaurs [44]. Here, the description of a new titanosaurian skeleton provides a critical glimpse into the Mtuka Member of the Galula Formation, offering better insight for comparisons with the neighboring Aptian? Dinosaur Beds of Malawi and other Afro-Arabian sauropod bearing strata. More significantly, this new species provides a window into the early evolutionary history of titanosaurian sauropods with one of the best represented skeletons of an individual titanosaurian that includes information from most regions of the body.

## Materials and methods

### Nomenclatural acts

The electronic edition of this article conforms to the requirements of the amended International Code of Zoological Nomenclature, and hence the new names contained herein are available under that Code from the electronic edition of this article. This published work and the nomenclatural acts it contains have been registered in ZooBank, the online registration system for the ICZN. The ZooBank LSIDs (Life Science Identifiers) can be resolved and the associated information viewed through any standard web browser by appending the LSID to the prefix “http://zoobank.org/”. The LSID for this publication is: urn:lsid:zoobank.org:act:9D98DF30-6588-4F9F-9869-EE2CFD30EF94. The electronic edition of this work was published in a journal with an ISSN, and has been archived and is available from the following digital repositories: PubMed Central, LOCKSS.

## Institutional abbreviations

**MAL**—Malawi Department of Antiquities, Lilongwe and Blantyre, Malawi; **RRBP**—Rukwa Rift Basin Project, Tanzanian Antiquities Unit, Dar es Salaam, Tanzania.

### Systematic paleontology

DINOSAURIA [45]

SAURISCHIA [46]

SAUROPODA [47]

TITANOSAURIFORMES [48]

TITANOSAURIA [49]

LITHOSTROTIA [19]

*MNYAMAWAMTUKA MOYOWAMKIA*, gen. et sp. nov.

ZooBank Life Science Identifier (LSID) urn:lsid:zoobank.org:act:9D98DF30-6588-4F9F-9869-EE2CFD30EF94

### Etymology

*Mnyamawamtuka* (Mm-nya-ma-wah-mm-too-ka), ‘mnyama’ is the Kiswahili word for ‘animal’ or ‘beast’ and acts as a conceptual proxy to the titans in Titanosauria, and ‘wa Mtuka' is Kiswahili for ‘of the Mtuka’ in reference to the river drainage that yielded the type specimen. *Moyowamkia* (Mm-oh-yo-wa-mm-key-ah), ‘moyo’ is the Kiswahili word for heart and ‘wa mkia’ is Kiswahili for ‘of the tail’, in reference to the posterolateral expansion of the posterior centrum on the middle caudal vertebrae that gives the posterior centrum surface a heart-shape outline.

### Holotype

RRBP 05834, a partial skeleton including an anterior cervical vertebral neural arch and four cervical vertebral centra, seven partial dorsal vertebrae, a sacral neural arch, three partial sacral centra, three sacral ribs, seven caudal vertebral neural arches and seven centra, four chevrons, numerous dorsal rib fragments, a right scapula, a right sternal plate, a partial left humerus and distal right humerus, partial left ulna, right metacarpal I and left metacarpal III, a partial left ischium, a partial right pubis, partial left and right femora, left tibia and partial right tibia, a left fibula, left metatarsal I, left metatarsal II, right metatarsal III, left metatarsal IV, left metatarsal V, two pedal phalanges, a left ungual, and numerous unidentifiable fragments. The majority of the fossils were prepared at the Ohio University Fossil Preparation Facility, with some of the first-discovered elements prepared by J. P. Cavigelli. Preperation used standard manual and technical techniques including hand tools and pneumatic air scribes. Repository information of RRBP 05834 is the Rukwa Rift Basin Project, Tanzanian Antiquities Unit, Dar es Salaam, Tanzania. The fossils are, at time of publication, on temporary loan and deposited at Ohio University in Athens, Ohio. All of the fossils are accessible by request. Research casts will permanently be housed at Ohio University and in the collections at Denver Museum of Science and Nature.

### Type locality and horizon

**Fig 1:**
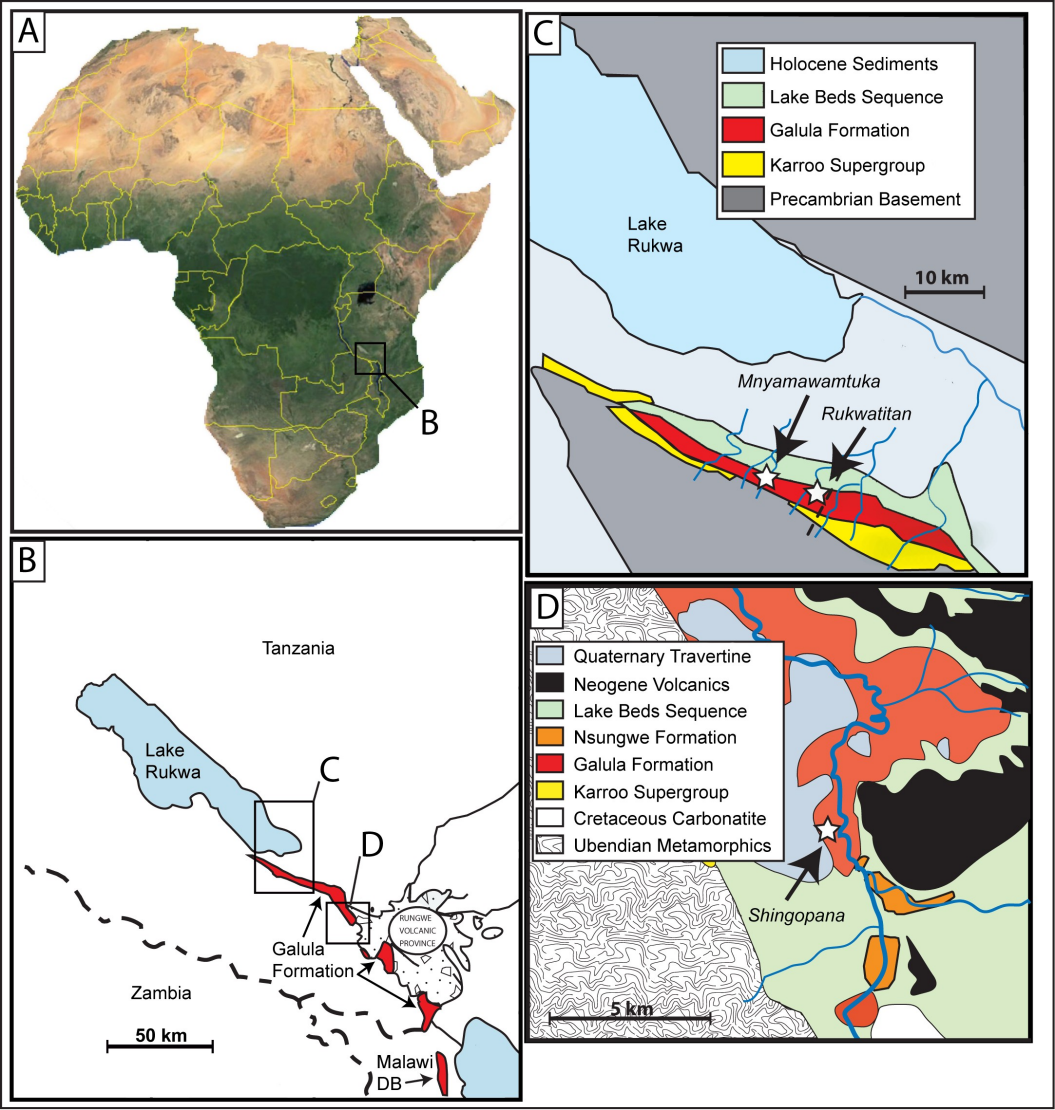
Map of research area. Map of Africa, A, with expanded regional map of the Rukwa Rift Basin of Tanzania, B, with the type localities of *Mnyamawamtuka moyowamkia* and *Rukwatitan bisepultus* quarry near the Galula study area, C, and the *Shingopana songwensis* quarry near the Nsungwe study area, D. Malawi Dinosaur Beds (DB) marked in B to demonstrate the proximity of the deposits to the Galula Formation.

**Fig 2:**
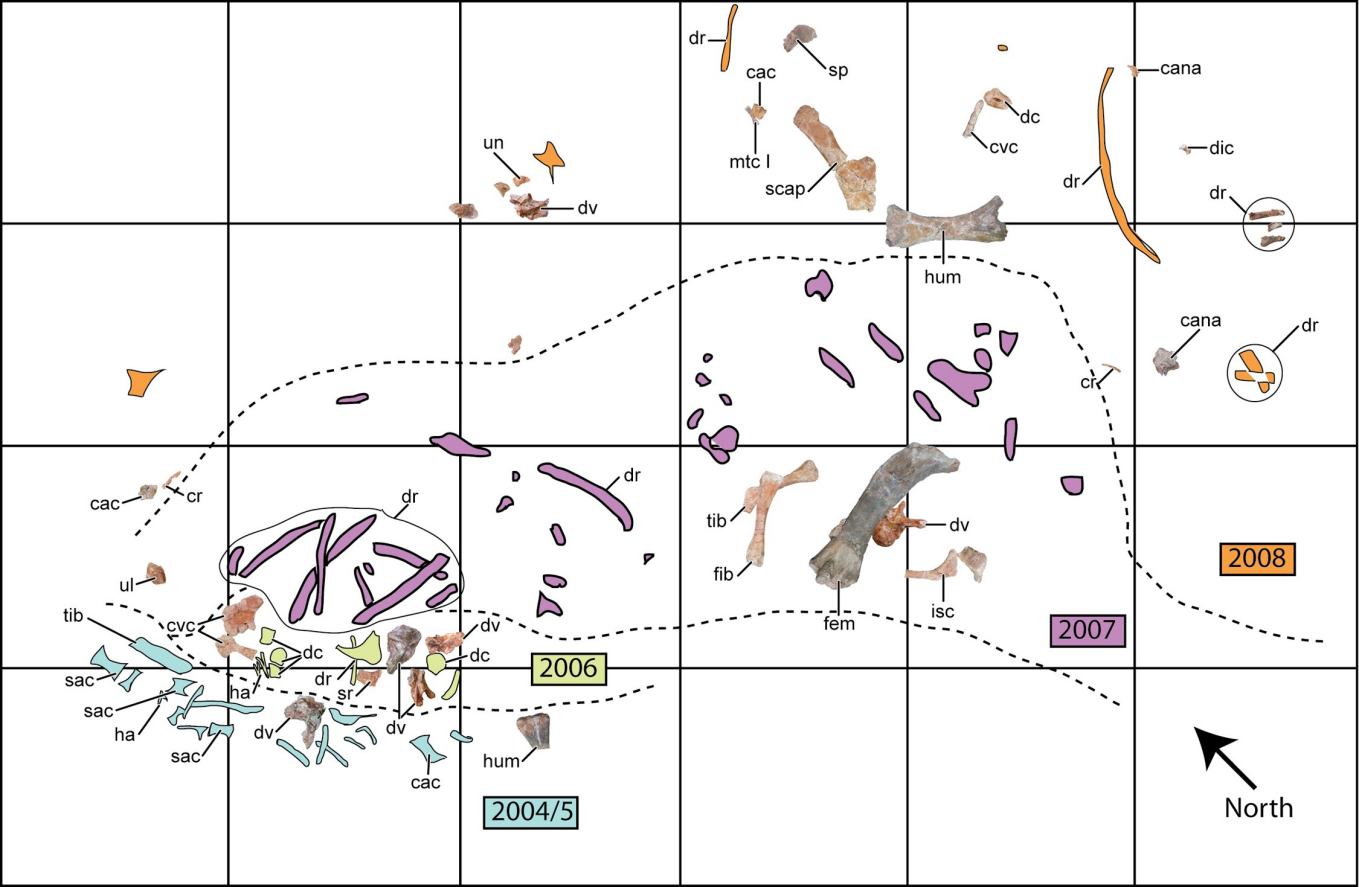
Quarry map. Quarry map of the Mtuka bonebed locality RRBP 2004–06. Recovered elements of *M*. *moyowamkia* are color-coded and separated by dashed lines according to the year they were collected. The quarry map is represented as a four-by-six-meter grid. Unmarked elements on the map are either fragments or unidentified. Abbreviations: cac, caudal vertebral centrum; cana, caudal vertebral neural arch; cr, cervical rib; cvc, cervical vertebral centrum; dc, dorsal vertebral centrum; dic, distal caudal vertebra; dr, dorsal rib; dv, dorsal vertebra; fem, femur; fib, fibula; ha, haemal arch; hum, humerus; isc, ischium; mtc I, metacarpal I; sac, sacral centrum; scap, scapula; sp, sternal plate; sr, sacral rib; tib, tibia; ul, ulna; un, ungual.

The specimen was recovered in the Mtuka Member of the Cretaceous Galula Formation. The Mtuka Member is dominated by coarse sandstone fluvial deposits and abundant overbank siltstone and mudstone lenses within an extensive fluvial braidplain system [44]. The holotype of *M*. *moyowamkia* was recovered from a quarry developed along the Mtuka River drainage in southwestern Tanzania (Fig 1). The quarry is roughly 20 kilometers south of Lake Rukwa near the coordinates of 32° 34’ E and 8° 34’ S. The initial discovery was made in 2004 at locality RRBP 2004–06, with additional elements recovered sequentially during the 2005–2008 field seasons by the Rukwa Rift Basin Project field teams (Fig 2). Generally, larger and more complete elements, such as appendicular remains, were recovered in the western part of the quarry whereas smaller and more fragmented elements were recovered from the eastern part of the quarry, indicating short-distance transport (Fig 2). Excavation permits were issued by The United Republic of Tanzania, Ministry of Natural Resources and Tourism, Antiquities Unit, P.O. Box 2280, Dar es Salaam, Tanzania to P. M. O’Connor under the specific permit numbers: 14–2004; EA 402/605/01; EA 402/605/01/78; EA 402/605/01/20; and EA 402/604/01/7. In a broader context, the *M*. *moyowamkia* discovery and excavation was made in the early years of the Rukwa Rift Basin Project with the aim of addressing the paucity of fossils recovered from the Cretaceous of sub-Saharan Africa [6].

### Age and distribution

The materials were recovered from the Mtuka Member of the Galula Formation of the Red Sandstone Group, Rukwa Rift Basin, southwestern Tanzania. Based on previous lines of evidence, including faunal data within the overlying Namba Member, the age of the Galula Formation was best constrained to the middle Cretaceous (Aptian–Cenomanian) with potential dates of 100–110 Ma. However, new paleomagnetic data place the Mtuka Member (i.e., the specific unit from which *M*. *moyowamkia* was recovered) within the Cretaceous long normal with estimates of Aptian–Cenomanian for the unit and a younger date for the overlying Namba Member as either Campanian or Cenomanian–Santonian (see [50] for a detailed discussion).

### Diagnosis

Titanosaurian sauropod dinosaur diagnosed by the following suite of characters: cervical vertebral neural canal narrows mid-length [21, 29]; postaxial cervical centra exhibit a shallow lateral fossa[33]; ventral midline keel absent in postaxial cervical vertebral centra [33]; anterior dorsal neural spines single, not bifurcated [33]; middle-posterior dorsal vertebrae with flat-top diapophysis [29, 33]; dorsal vertebrae lack hyposphene and hypantrum articulations [18, 29, 33]; middle–posterior dorsal vertebrae with dorsolaterally oriented diapophysis [33]; anterior caudal neural spines project dorsally [33]; estimated humerus length roughly 80% of femur length [18, 29]; humerus with posterolateral bulge near level of deltopectoral crest [29]; humerus with undivided radial condyle ([29, 33]); ratio of mediolateral width of distal end of tibia relative to long-axis length of midshaft width is greater than 2.0 [33]; anteromedially deflected crest of the proximal fibula [33]; tuberosity along ventral margin of pedal ungual [33].

*Mnyamawamtuka moyowamkia* is diagnosed by the following suite of autapomorphies: (1) middle and posterior dorsal vertebrae with vertical lamina between neural canal and interprezygapophyseal lamina that bifurcates dorsally; (2) posterior dorsal vertebra with no interpostzygapophyseal lamina as the postspinal lamina continues to the dorsal margin of the neural canal; (3) prominent dorsolateral expansion on the posterior centrum of the middle caudal vertebra; (4) curved crest with accompanying fossa within the dorsomedial region of the proximal scapular blade; (5) sternal plate unusually small, estimated to be, at most, 42% of humerus length.

## Description

### Teeth

**Fig 3:**
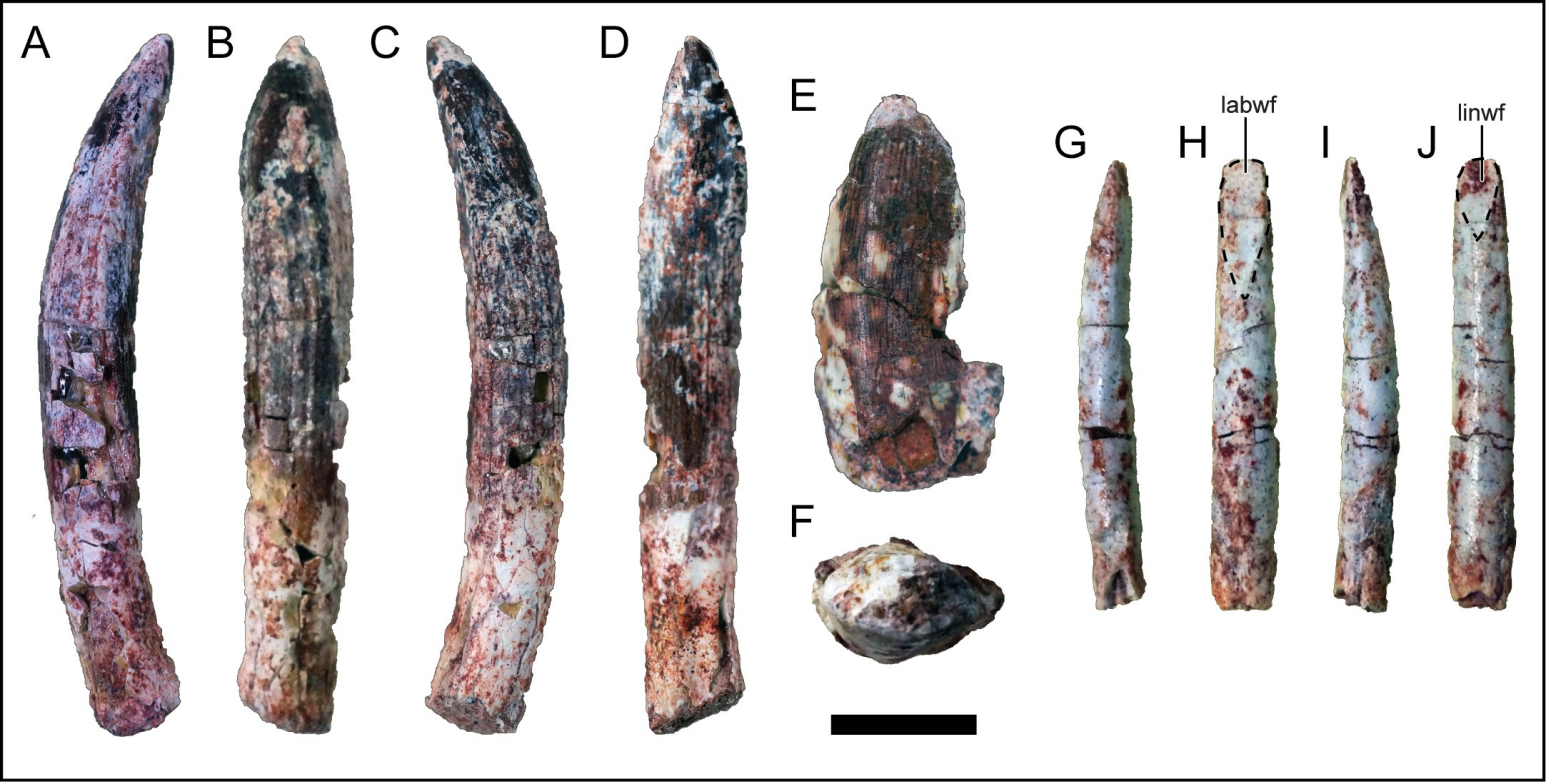
Teeth associated with *Mnyamawamtuka moyowamkia* skeleton. Teeth recovered from the *Mnyamawamtuka moyowamkia* quarry. A–D, tooth Morph A; E–F, tooth Morph B; and G–J, tooth Morph C. A, G, distal; B, E, H, labial; C, I, mesial; D, J, lingual; and F, occlusal views. Abbreviations: labwf, labial wear facet; linwf, lingual wear facet. Scale bar equals 1 cm.

Four teeth that can best be categorized into three different morphs were recovered from the quarry (Fig 3). Titanosaurians that preserve nearly complete skulls and/or dentigerous elements, for example, *M*. *dixeyi* [3, 34], *Nemegtosaurus mongoliensis* [51], *Sarmientosaurus musacchioi* [52], and *T*. *macedoi* [35, 36], exhibit a range of wear patterns and general tooth morphologies. It is unlikely that the tooth morphs indicate the presence of multiple individuals within the quarry due to the lack of repetitive or size inconsistent elements and the uncommon occurrence of shed sauropod teeth within the Galula Formation. Morph A (Fig 3A–3D) is consistent with a morphology that is traditionally attributed to some non-titanosaurian titanosauriforms by exhibiting a near D-shaped cross section along the crown, presence of weakly developed mesial and distal denticles, and longitudinal texturing [18]. However, Morph A does not resemble the spatulate tooth morphology best represented in *Camarasaurus* and non-titanosaurian titanosauriformes. Based on the detailed description of the nearly complete skull of the Aptian *T*. *macedoi* from Brazil [36] and the skull of the Cenomanian *S*. *musacchioi* from Argentina [52], Morph A is consistent with the mesial left maxillary tooth position by exhibiting a slight apicobasal twist and asymmetry along the mesial and distal margins (the former straighter and the latter more curved). Morph A does not exhibit any wear facets so the occlusal pattern is unknown. Although only partially preserved, Morph B is like the teeth observed in the tooth-bearing elements of *M*. *dixeyi* ([3, 34]; E.G. Pers. obvs., 2014, 2015). The general morphology is intermediate between the basal D-shape cross-sectional morphology and derived cylindrical cross-section within titanosauriforms (Fig 3D–3E), and the morph is grossly similar to the recovered teeth of *Ampelosaurus atacis* from the Maastrichtian of France [53. 54], *M*. *dixeyi* [3, 34], and *S*. *musacchioi* from the Cenomanian of Argentina [52]. There are no wear facets present and it does not appear to twist along the long axis. Morph B is relatively wider mesiodistally than Morph A. Therefore, Morph B is inferred to be within the mesial position of the upper tooth row, likely the premaxilla, as teeth of a comparable morphology are in *T*. *macedoi* and *S*. *musacchioi* [36, 52]. Longitudinal texturing is also present on the surface of Morph B. The two teeth of Morph C represent the traditional titanosaurian tooth morphology (Fig 3G–3J), exhibiting a cylindrical cross section along the slender tooth crown with both labial and lingual high-angled wear facets [29, 33]. The labial wear facet is elliptical and longer in maximum length than the subcircular lingual wear facet. The Morph C teeth likely derive from a mesial position due to the lack of asymmetrical twisting along the apicobasal axis and the presence of both labial and lingual wear facets; however, the size of the tooth is smaller than morph A and B and may represent an earlier stage of tooth replacement or different tooth position entirely [36]. Morph C appears to be distinct from the other two tooth morphs but this may be due to positional variation, stages of replacement, or even the possibility of an isolated tooth from a different sauropod altogether. In the absence of a fully preserved dentary, maxilla, or premaxilla, *M*. *moyowamkia* may have captured the transition of tooth morphology into the derived peg-like condition seen in most titanosaurians.

## Cervical vertebrae

Cervical vertebrae consist of four isolated centra (including representatives from the anterior, middle and posterior regions), and a single anterior cervical neural arch. The lack of fusion between the recovered cervical vertebral centra and neural arch suggests *M*. *moyowamkia* had not reach skeletal maturity at time of death. This is further corroborated by the unfused condition exhibited by most of the recovered axial skeleton described below. Cervical vertebral centra are moderately well preserved; however, the centra only preserve the base of the parapophysis. All recovered cervical and dorsal vertebral elements, where breakage and erosional surfaces are present, display internal camellate texturing typically seen in somphospondylian titanosauriforms [18, 29, 33]. All cervical vertebral centra lack a ventral keel, a characteristic typical in some capacity within the cervical series in most macronarian sauropods [33]. As currently preserved, *M*. *moyowamkia* does not exhibit the autapomorphies present in the cervical vertebrae of *M*. *shahinae* [16], *R*. *bisepultus* [11], and *S*. *songwensis* [12], further differentiating this taxon from other African titanosaurians.

### Anterior cervical vertebra

**Fig 4:**
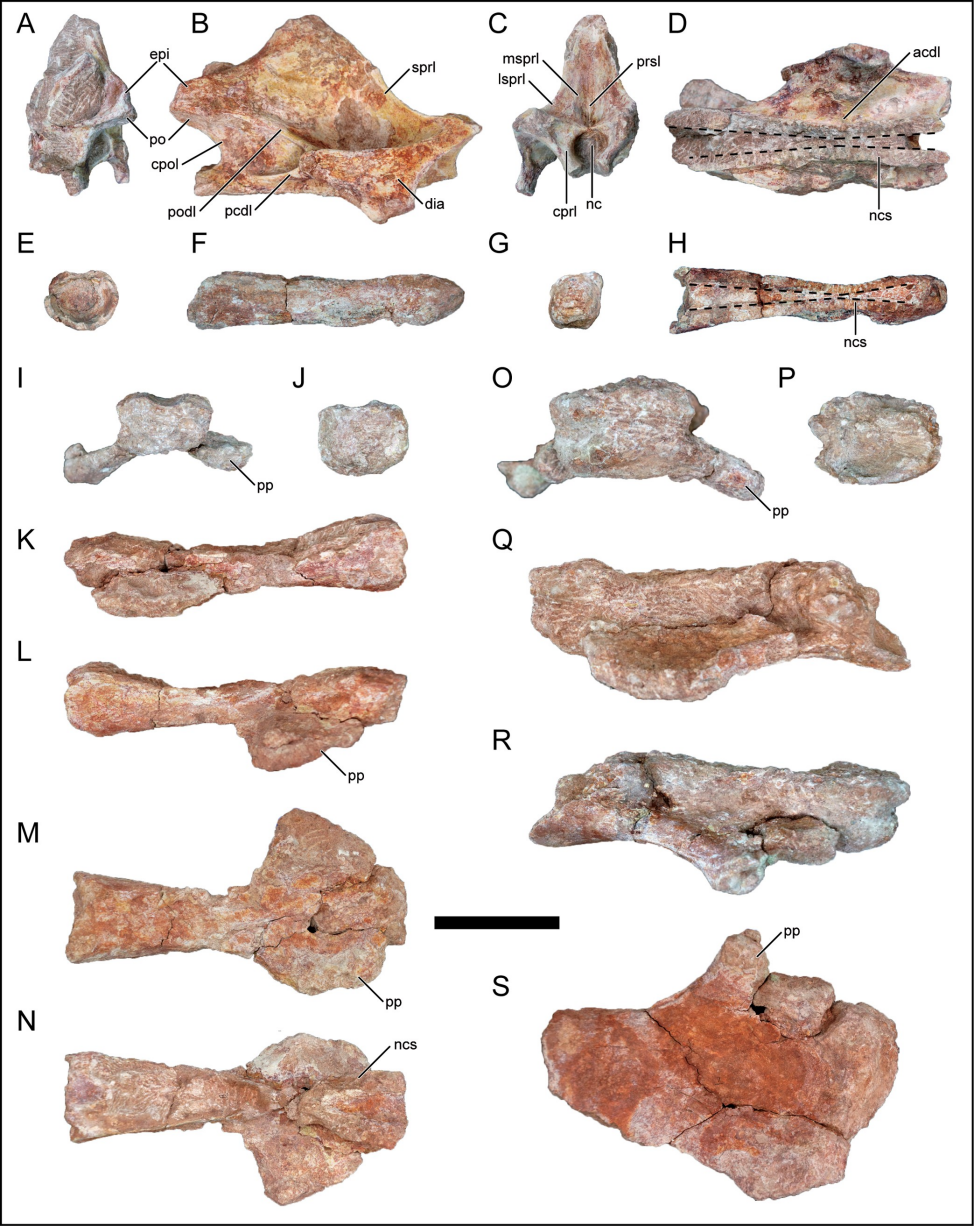
Cervical vertebrae of *Mnyamawamtuka moyowamkia*. A–D, anterior cervical neural arch; E–H, anterior cervical centrum; I–N, middle cervical centrum; and O–S, posterior cervical centrum. A, E, J, P, posterior; B, F, L, R, right lateral; C, G, I, O, anterior; D, M, S, ventral (anterior to the right); D, H, N, dorsal views (anterior to the right), and K, Q, left lateral views. Abbreviations: acdl, anterior centrodiapophyseal laminal; cpol, centropostzygapophyseal lamina; cprl, centroprezygapophyseal lamina; dia, diapophysis; epi, epipophysis; lsprl, lateral spinoprezygapophyseal ramus; msprl, medial spinoprezygapophyseal lamina ramus; nc, neural canal; ncs, neurocentral suture; pcdl, posterior centrodiapophyseal lamina; po, postzygapophysis; podl, postzygodiapophyseal; lamina; pp, parapophysis; prsl, prespinal lamina; sprl, spinoprezygapophyseal lamina. Scale bar equals 10 cm.

An anterior cervical neural arch (Fig 4A–4D) and a single anterior cervical centrum (Fig 4E–4H) were recovered from the quarry and do not precisely match enough to be considered the same vertebra. The centrum is elongate with subequal height and width at both anterior and posterior ends (Table 1). The ventral surface is nearly flat and is only slightly concave at the level where the parapophysis would have been had it been preserved on this element. Similarly, the centrum narrows (transversely) at this region and forms a shallow fossa. Furthermore, the neural canal, neurocentral sutures, and pedicles narrow here (Fig 4D and 4H). In *R*. *krausei* from the Late Cretaceous of Madagascar, the constriction is located near vertebral mid-length in the anterior cervical vertebrae and migrates anteriorly when progressing posteriorly along the cervical vertebral series [55]. The narrowing of the neural canal within the cervical series has been proposed as an autapomorphy for *R*. *krausei* [55–57], and has since been recognized to be a more widespread trait as a potential synapomorphy for Saltasauridae titanosaurians (29). However, D’Emic [29] mainly focused on the relationships of non-titanosaurian titanosauriforms and this trait may be more common within titanosaurians than previously anticipated. Concerning the recovered neural arch, the transverse process projects laterally from the neural arch with a triangular and flat lateral surface that narrows ventrally to the diaphysis that articulates with the tuberculum of the cervical rib. A weakly developed anterior centrodiapophyseal lamina (Fig 4B) is present along the ventral surface of the diapophysis and the short posterior centrodiapophyseal lamina (Fig 4B) terminates prior to the posterior margin of the pedicle. The centroprezygapophyseal lamina is singular (Fig 4C), and differs from the divided condition seen in saltasaurid titanosaurians [18, 29, 33]. Similarly, the singular centropostzygapophyseal lamina is vertically oriented. The prezygapophysis is poorly preserved whereas the subcircular postzygapophysis faces ventrally (Fig 4A). The epipophysis is dorsal to the posterior margin of the postzygapophysis (Fig 4B), and is not strongly developed nor posteriorly protruding as in Asian euhelopodid titanosauriforms (29). The spinoprezygapophyseal lamina is directed posterodorsally and exhibits a weak kink that occurs roughly halfway along the lamina (Fig 4B). This is similar to the condition in the cervical vertebrae attributed to *M*. *dixeyi* (E.G. Pers. obvs., 2014, 2015). The right spinoprezygapophyseal lamina is weakly divided into lateral and medial segments near the lateral and medial margins of the prezygapophysis, respectively (Fig 4C). The prespinal lamina is only partially developed near its base. The spinopostzygapophyseal lamina courses gently anterodorsally towards the neural spine. The dorsal portion of the neural spine is slightly thickened transversely and mildly rugose like the anterior cervical vertebrae in *Saltasaurus loricatus* [58] and *Maxakalisaurus topai* [59].

**Table 1:** Select measurements of the axial skeleton of *Mnyamawamtuka moyowamkia*.

	CL	ACH	ACW	PCH	PCW	TH	NAH	NASH	NATP
**Anterior Cervical**	211	37	32	42	52	-	-	-	-
**Middle Cervical**	263	-	-	55	70	-	-	-	-
**Posterior Cervical**	285	71	88	-	-	-	-	-	-
**DV 1**	-	-	-	-	-	-	106	181	195*
**DV 2**	-	-	-	-	-	-	73	148	210*
**DV 3**	162	87	130	143	109	284	60	179	178
**DV 4**	-	-	-	-	-	-	-	-	-
**DV 5**	-	-	-	-	-	-	60	155	153
**DV 6**	-	-	-	-	-	-	82	209	118
**DV 7**	-	-	-	-	-	-	91	204*	126
**Example Dorsal Centrum**	142*	-	-	108	-	-	-	-	-
**Dorsal Centrum**	113*	103	-	92	-	-	-	-	-
**Sacral Centrum 1**	99	56	-	46	94	-	-	-	-
**Sacral Centrum**									
**Anterior Caudal**	59	97	110	82	92	196	36	115	100
**Middle Caudal Vertebra**	92	63	69	62	69	104	16	32	
**Middle Caudal Centrum A**	64	66	81	58	71	-	-	-	-
**Middle Caudal Centrum B**	-	-	-	70	76	-	-	-	-
**Middle-Distal Caudal Centrum A**	60	-	-	73	85	-	-	-	-
**Middle-Distal Caudal Centrum B**	68	63	59	57	59	-	-	-	-
**Distal Caudal Centrum**	88	59	58	57	62	-	-	-	-

Abbreviations: ACvC, anterior cervical vertebral centrum; ACH, anterior centrum height; ACV, anterior caudal vertebra; ACW, anterior centrum width; CL, total centrum length; DCC, distal caudal centrum; DV, dorsal vertebra; DC, dorsal centrum; EDC, example dorsal centrum; MCvC, middle cervical vertebra; MCC, middle caudal centrum, MCV, middle caudal vertebra; MDC, middle-distal caudal centrum; NAH, neural arch height, base to ventral portion of postzygapophysis; NASH, neural arch and neural spine height; NATP, neural arch width, midpoint to lateral tip of the transverse process; PCvC, posterior cervical vertebra; PCH, posterior centrum height; PCW, posterior centrum width; SVC, sacral vertebral centrum; TH, total centrum and neural arch height.

* denotes measurement of incomplete area of fossil. All measurements are in mm.

### Middle and posterior cervical vertebrae

A single middle cervical centrum was recovered from the quarry (Fig 4I–4N). Although rather poorly preserved, most of the centrum and proximal portions of both parapophyses remain intact. The elongation index of the centrum (length: average of anterior and posterior height) is approximately 5.1, surpassing either the 4.0 [33] and 3.0 threshold [29] for titanosauriformes (Table 1). Elongation of middle cervical centra is common among titanosauriforms with few exceptions: the Late Cretaceous titanosaurians *Isisaurus colberti* [60] and *Mendozasaurus neguyelap* [61] (however see [62]). The opisthocoelous centrum and neural canal drastically narrows roughly one-third the distance from the anterior end (Fig 4N). The parapophysis projects laterally from the anterior half of the centrum, differing from euhelopodids where the parapophysis projects ventrally [29]. The ventral surface is weakly concave at the level of the parapophysis and does not exhibit a keel, as in most macronarians [33]. Two posterior cervical centra have been recovered; however, they are poorly preserved (Fig 4O–4S). The morphology is similar to the middle cervical centrum albeit slightly larger and proportionally broader in size due to its posterior position in the cervical series (Table 1). The recovered cervical vertebrae do not exhibit well-defined pleurocoels.

### Dorsal vertebrae

The dorsal series of *Mnyamawamtuka moyowamkia* is represented by a virtually complete anterior-middle dorsal vertebra, several fragmentary centra and six partial-to-nearly complete neural arches from the anterior, middle, and posterior regions. Although the complete number of dorsal vertebrae is unknown and the exact position of each recovered dorsal vertebra is somewhat uncertain, the dorsal vertebrae will be described by inferred relative order and demarcated as such (e.g., D1, D2, etc.). Relative order is estimated based on position of the dorsoventral position of the parapophysis along the neural arch, angle and distance between each prezygopophyses and postzygapophyses, length and angle of the transverse process, and general morphology compared to more complete dorsal vertebral series from other titanosauriform sauropods (e.g., *R*. *krausei* [55]) (Table 1). Similar to the cervical vertebrae, the internal texture of the dorsal vertebrae is camellate, typical of most titanosauriform sauropods [18, 29, 33]. The recovered dorsal neural arches do not exhibit a hyposphene-hypantrum complex; the absence of these features within the middle and posterior dorsal vertebral region is typical in most titanosaurians [18, 29, 33, 48]. Collectively, the neural spines are not bifurcated, a condition that is variably present throughout non-titanosaurian sauropods [18, 29, 33], and the only titanosaurian known with bifid dorsal neural spines is the enigmatic *Opisthocoelicaudia skarzynskii* from the Late Cretaceous of Mongolia [63]. Additionally, the neural spines do not exhibit aliform processes, a condition present variably within somphospondylian titanosauriforms such as *Argentinosaurus huinculensis* from the Cenomanian of Argentina and *Diamantinasaurus matildae* from the Cenomanian of Australia [21, 33, 64, 65].

#### Dorsal centra

**Fig 5:**
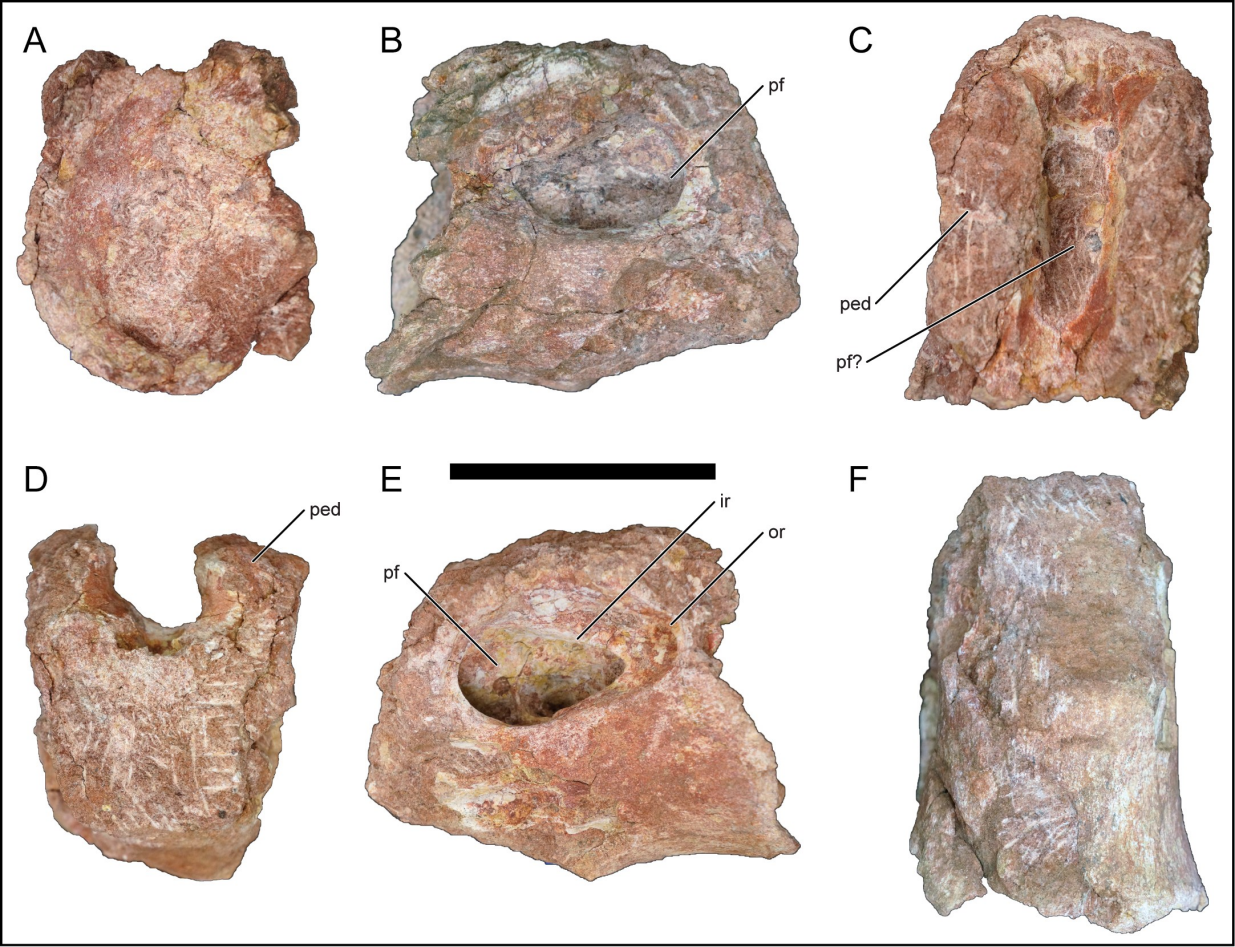
Example dorsal vertebra centrum of *Mnyamawamtuka moyowamkia*. A, anterior; B, right lateral; C, dorsal, D, posterior; E, left lateral; and F, ventral views; anterior to the top of page in C and F. Abbreviations: ir, inner rim of pleurocoel; or, outer rim of pleurocoel; ped, pedicle; pf, pneumatic fossa. Scale bar equals 10 cm.

Ten partial dorsal centra were recovered; however, due to poor preservation some of these centra may represent sacral (or dorsosacral) centra. Of the dorsal centra that are well preserved, all are opisthocoelous and exhibit an oval pleurocoel on the lateral surface. One representative dorsal centrum (Fig 5) exhibits a deep and undivided pleurocoel that preserves an outer ring and an inner ring that demarcates the margins of the left pleurocoel (Fig 5E). The preserved dorsal centra are taller than wide and derive from the middle–posterior region of the dorsal series. Typically, anterior dorsal centra are wide as they exhibit transitional morphology with the posterior cervical vertebrae that also exhibit wide centra (e.g., *R*. *krausei*: [55]). The neural canal does not constrict as in the cervical vertebrae but the neurocentral suture remains anteroposteriorly elongate. In one isolated dorsal centrum, a potential pneumatic space may be present along the dorsal surface of the centrum (i.e., the floor of the neural canal), but this may be due to erosional influences (Fig 5C).

#### Dorsal vertebra 1

**Fig 6:**
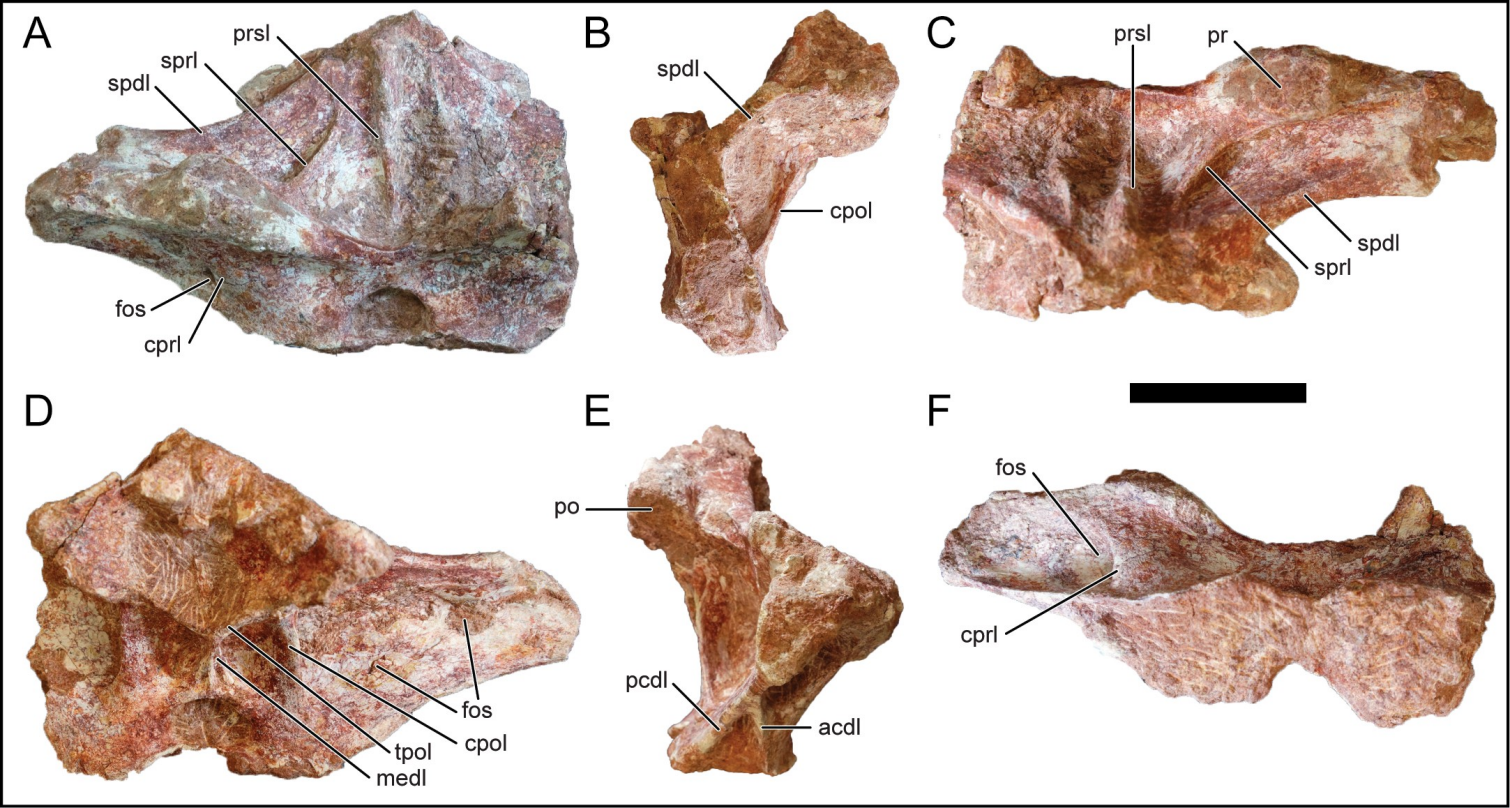
Anterior dorsal neural arch, D1, of *Mnyamawamtuka moyowamkia*. A, anterior; B, left lateral; C, dorsal; D, posterior; E, right lateral; and F, ventral views; anterior to the top of page in C and F. Abbreviations: acdl, anterior centrodiapophyseal laminal; cpol, centropostzygapophyseal lamina; cprl, centroprezygapophyseal lamina; fos, fossa; medl, median lamina; pcdl, posterior centrodiapophyseal lamina; po, postzygapophysis; pr, prezygapophysis; prsl, prespinal lamina; spdl, spinodiapophyseal laminal; sprl, spinoprezygapophyseal lamina; tpol, interpostzygapophyseal lamina. Scale bar equals 10 cm.

D1 may represent the cervicodorsal transitional vertebra based on the absence of the parapophysis on the neural arch, a dorsoventrally low neural arch, wide and low-angled prezygapophysis and postzygapophysis, elongated transverse process, and a short anteroposterior length (Fig 6). The neural arch is well preserved, and missing much of the left transverse process. The prezygapophysis is elliptical and faces mostly dorsally (Fig 6C) whereas the elliptical postzygapophysis faces ventrolaterally and is placed higher on the neural arch relative to the dorsoventral position of the prezygapophysis. The neural spine is low with the apex just dorsal to the level of the transverse process. A low neural spine of the anterior dorsal vertebrae is exhibited in the titanosaurians *R*. *krausei* [55], *M*. *dixeyi* (MAL- 236, MAL-238, [3]; E. G., pers. obvs., 2014, 2015), and *Muyelensaurus pecheni* [66].

The undivided centroprezygapophyseal lamina connects to the lateral margin of the prezygopophysis and forms the anterior border of a fossa that is bounded posteriorly by the transverse process (Fig 6A and 6F). A small fossa is present on the anterior face of the centroprezygopophyseal lamina ventral to the prezygopophysis (Fig 6A), and is similar to the condition in the non-titanosaurian titanosauriform *Chubutisaurus insignis* from the middle Cretaceous of Argentina [67]. The anterior and posterior centrodiapophyseal laminae merge together halfway along the ventral surface of the transverse process and continue as a single lamina to the diapophysis. The centropostzygapophyseal lamina is vertically oriented and buttresses the medial postzygapophysis margin. The interpostzygapophyseal lamina is present and V-shaped (Fig 6D). A vertical lamina runs from the interpostzygapophyseal lamina and contacts the neural canal, a trait also present in *R*. *krausei* [55] and the anterior dorsal vertebrae attributed to *M*. *dixeyi* (MAL-236 and MAL-239 [3]; E.G., pers. obs., 2014). The well-defined centropostzygapophyseal fossa is bounded by the vertical, interpostzygapophyseal, and centropostzygapophyseal laminae (Fig 6D). The prespinal lamina is undivided along its course. The spinoprezygapophyseal lamina is weakly developed and does not fully connect with the prezygapophysis. The postspinal lamina is poorly preserved within the wide spinopostzygapopyhseal fossa.

#### Dorsal vertebrae 2 and 3

**Fig 7:**
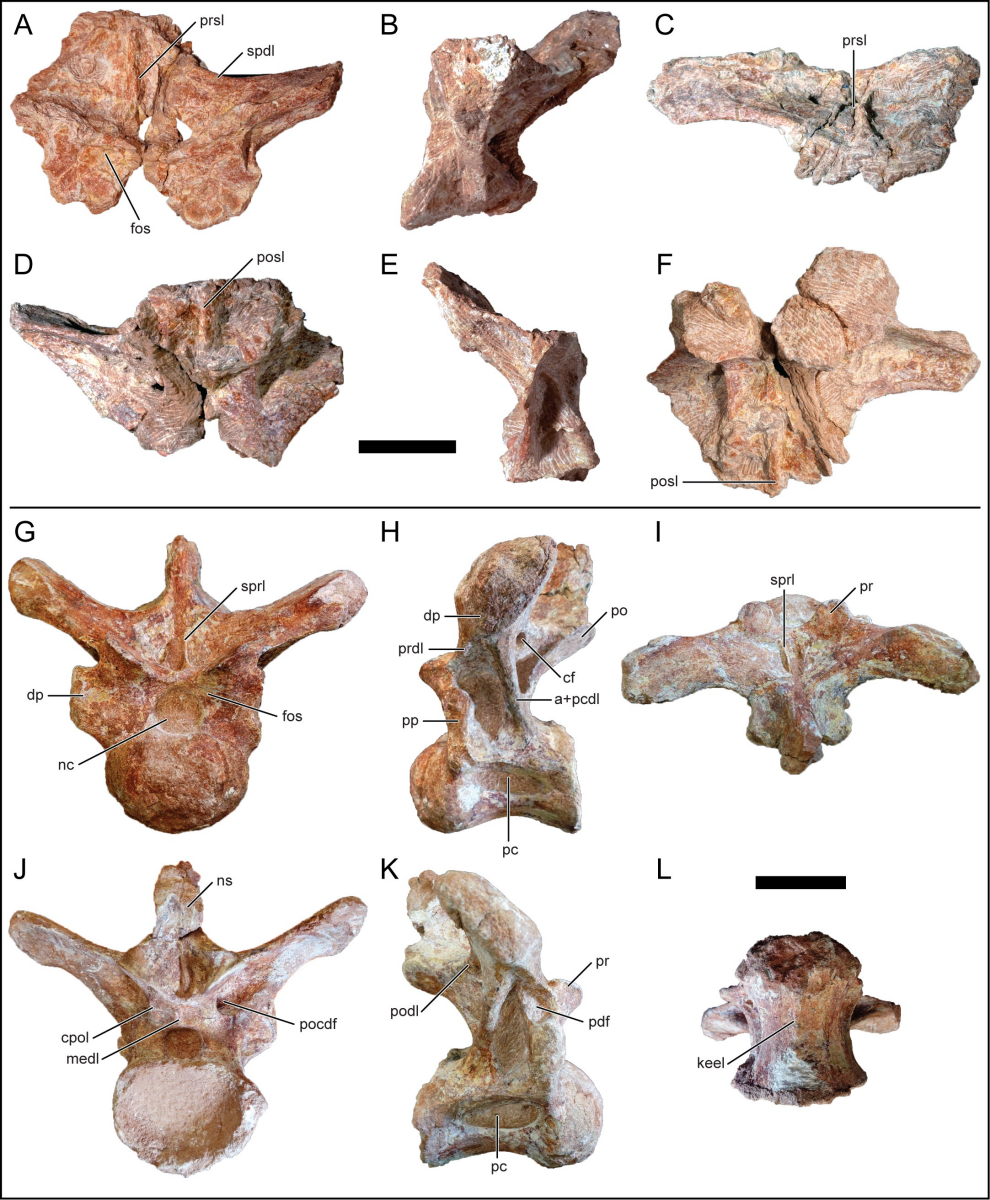
Anterior dorsal vertebrae of *Mnyamawamtuka moyowamkia*. A–F, anterior dorsal neural arch D2, and G–L, vertebra D3. A, G, anterior; B, H, left lateral; C, I, dorsal; D, J, posterior; E, K, right lateral; and F, L, ventral views. Anterior towards the top of the page in C, F, I, and L. Abbreviations: a+pcdl, merger of anterior and posterior centrodiapophyseal laminae; cf, circular foramen; cpol, centropostzygapophyseal lamina; dp, diapophysis; fos, fossa; medl, medial lamina; nc, neural canal; posl, postspinal lamina; pc, pleurocoel; pdf, paradiapophyseal lamina fossa; pocdf, postzygapophysis centrodiapophyseal fossa; pp, parapophysis; po, postzygapophysis; podl, postzygodiapophyseal lamina; prdl, prezygodiapophyseal laminal; prsl, prespinal lamina; spdl, spinodiapophyseal lamina; sprl, spinoprezygapophyseal lamina. Scale bar equals 10 cm.

D2 is represented by a partial neural arch (Fig 7A–7F) whereas D3 is exquisitely preserved (Fig 7G–7L). Overall, both vertebrae are similar to one another and the description will focus on D3 unless noted otherwise. The centrum is opisthocoelous and is slightly wider than tall. The ventral surface is concave along the long axis and exhibits a keel (Fig 7L). A ventral keel is present in the titanosauriforms *Brachiosaurus altithorax*, *Euhelopus zdanskyi*, and the titanosaurians *M*. *neguyelap* [61], *D*. *matildae* [65], and *O*. *skarzynskii* [33]. The lateral surface of the centrum exhibits an anteroposteriorly elongate pleurocoel that tapers posteriorly (Fig 7H), a feature that was previously considered diagnostic for titanosaurians [48], and has since been recognized to be more prevalent within macronarian sauropods [33].

The neural canal is subcircular anteriorly whereas its posterior opening is dorsoventrally compressed (Fig 7G and 7J). The parapophysis is located at the junction of the pedicle and centrum, suggesting an anterior-middle position within the dorsal vertebral series. The diapophysis is rounded and faces ventrolaterally. The prezygapophysis faces dorsomedially and the articular facet is subcircular. The postzygapophysis is angled ventrolaterally and the articular facet is elliptical. The transverse process is directed dorsolaterally at a low angle (~30 degrees) from the horizontal, differing from the horizontally-oriented transverse process on D1 and D2. The posterior surface of the transverse process is broad. Near the medial region of the left transverse process in D3, but not D2, lies a circular foramen just anterior to the postzygapophysis (Fig 7H). By contrast, the right transverse process preserves a shallow postzygapophyseal centrodiapophyseal fossa in this position (Fig 7J). The presence of the foramen/fossa in this area is exhibited in several of the anterior dorsal vertebrae of *M*. *dixeyi* (MAL-236, MAL-238; E. G., pers. obvs., 2014, 2015). The neural spine is compressed transversely and is dorsally angled with the posterior margin oriented vertically above the postzygapophysis.

The centroprezygapophyseal lamina is broad along its anterior face due to position of the parapophysis. The centroprezygapophyseal lamina of D2 exhibits a shallow fossa ventral to the prezygapophysis (Fig 7A), and is absent in D3. In both D2 and D3, a fossa lies within the right paradiapophyseal lamina (Fig 7K); however, the left paradiapophyseal lamina in D3 consists of a single lamina without an equivalent fossa. The centropostzygapophyseal lamina and median vertical lamina is comparatively short relative to that in D1 and D2. The posterior centrodiapophyseal lamina is well-developed. The prespinal lamina runs along the distal portion of the neural spine and tapers proximally. The spinoprezygopophyseal lamina originates near the midpoint of the neural spine, just lateral to the prespinal lamina, and is more developed in D2 than in D3. The postspinal lamina is within the relatively reduced postspinal fossa and the spinodiapophyseal lamina is developed only as a low ridge.

#### Dorsal vertebra 4

**Fig 8:**
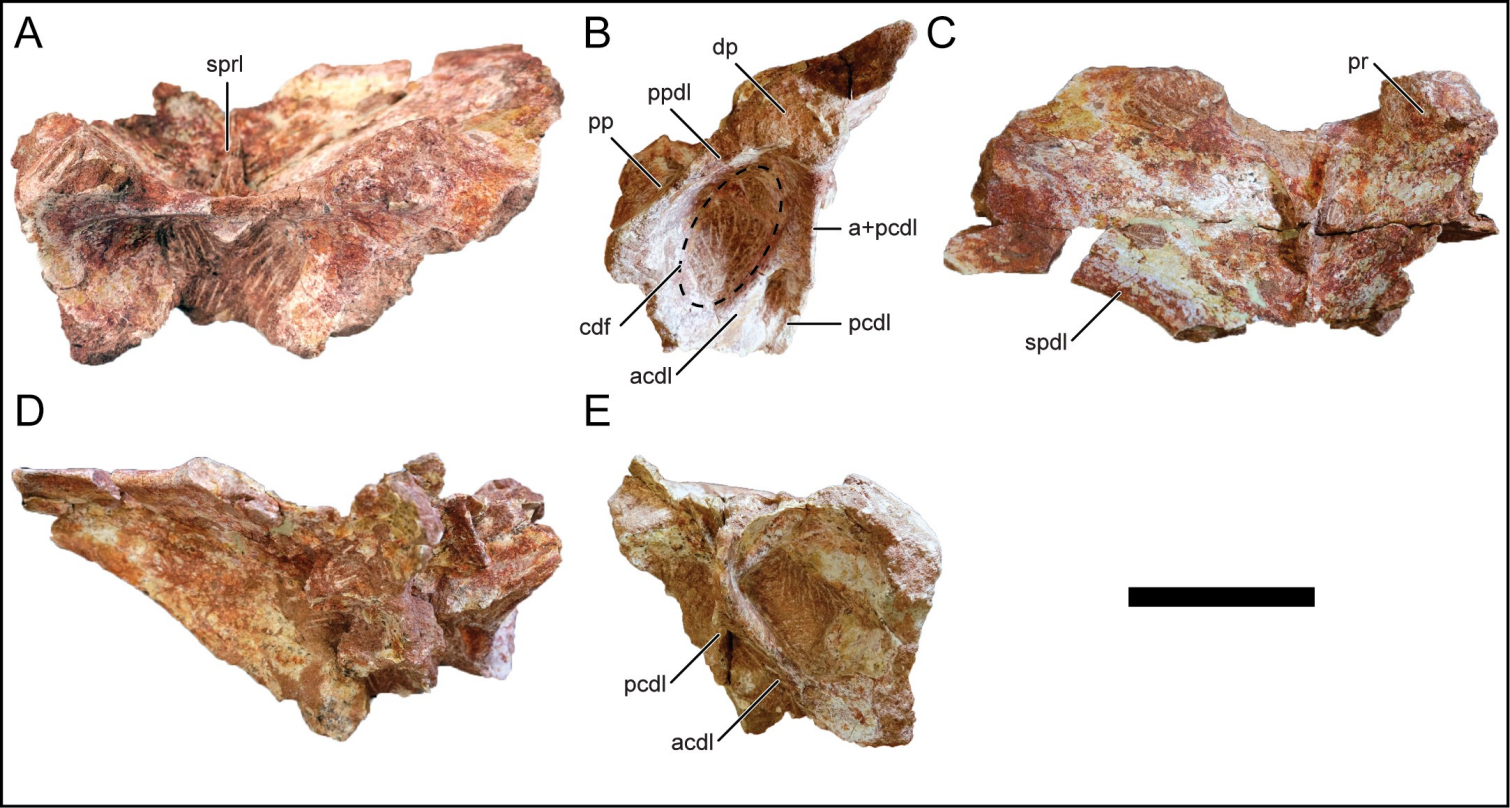
Middle dorsal neural arch, D4, of *Mnyamawamtuka moyowamkia*. A, anterior; B, left lateral; C, dorsal; D, posterior; and E, right lateral views. Anterior towards the top of the page in C. Abbreviations: a+pcdl, merger of anterior and posterior centrodiapophyseal laminae; acdl, anterior centrodiapophyseal lamina; cdf, centrodiapophyseal fossa; dp, diapophysis; pcdl, posterior centrodiapophyseal lamina; pp, parapophysis; ppdl, paradiapophyseal lamina; pr, prezygapophysis; spdl, spinodiapophyseal lamina; sprl, spinoprezygapophyseal lamina. Scale bar equals 10 cm.

D4 is represented by a fragmentary neural arch (Fig 8). The parapophysis is located on the neural arch supporting a more posterior dorsal position than D1–3 (Fig 8B). The left transverse process, similar to D2 and D3, is at a low angle relative to the horizontal but does not preserve much of the diapophysis at its distal end. The prezygapophyseal articular facet is circular and faces dorsomedially. The postzygapophysis and peripheral morphologies are too damaged for adequate description. The neural spine is not preserved, but based on the left spinodiapophyseal lamina, it is inferred to have been angled posterodorsally. There is an enlarged centrodiapophyseal fossa located lateral to the centroprezygapophyseal lamina (Fig 8B). The anterior centrodiapophyseal lamina is angled posterodorsally and merges with the posterior centrodiapophyseal lamina in a relative ventral position. The spinoprezygopophyseal lamina is remarkably reduced and both left and right laminae merge near the midline and continues dorsally (Fig 8A).

#### Dorsal vertebra 5

**Fig 9:**
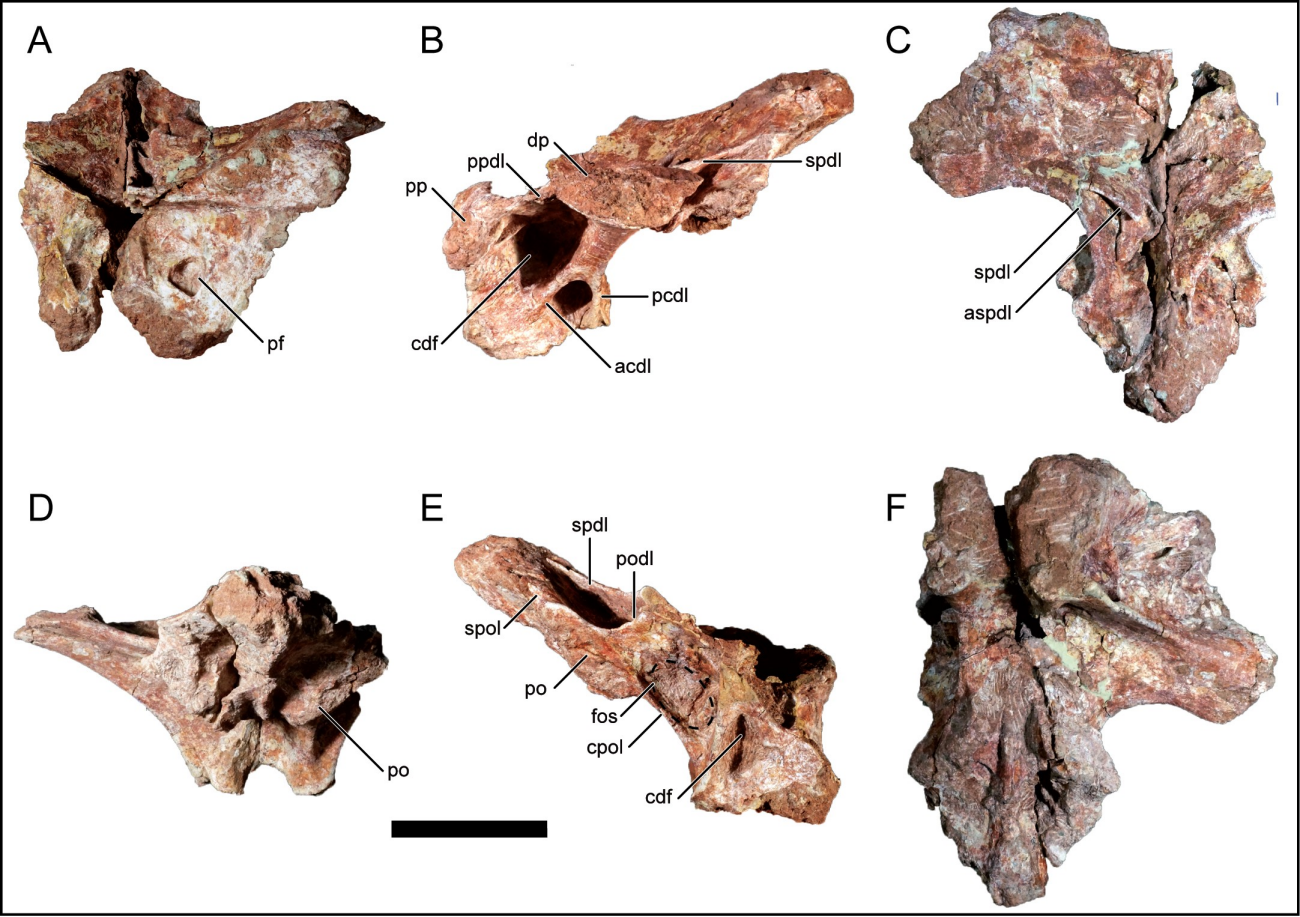
Middle dorsal neural arch, D5, of *Mnyamawamtuka moyowamkia*. A, anterior; B, left lateral; C, dorsal; D, posterior; E, right lateral; and F, ventral views. Anterior towards the top in C and F. Abbreviations: acdl, anterior centrodiapophyseal lamina; aspdl, accessary spinodiapophyseal lamina; cdf, centrodiapophyseal fossa; cpol, centropostzygapophyseal laminal; dp, diapophysis; fos, fossa; pcdl, posterior centrodiapophyseal lamina; pf, pneumatic fossa; po, postzygapophysis; podl, postzygadiapophyseal lamina; pp, parapophysis; ppdl, paradiapophyseal lamina; spdl, spinodiapophyseal lamina; spol, spinopostzygapophyseal laminal. Scale bar equals 10 cm.

D5 consists of the left side of the neural arch and only the proximal right side (Fig 9). The preserved parapophysis is located at the level of the prezygapophysis and just below the level of the diapophysis (Fig 9B). The parapophysis is oval and with a vertically-oriented long axis. On the posterior face of the parapophyseal portion of the transverse process, there is a small elliptical fossa that is similar to those in the more anterior dorsal vertebrae (Fig 9E). The partial transverse process is at a low angle relative to the horizontal. The prezygapophysis is slightly raised, exhibits an elliptical articular facet, and is angled dorsomedially. The postzygapophysis is angled ventrolaterally; however, the morphology medial to the postzygapophysis is not well preserved. The centroparapophyseal lamina is angled anterolaterally and is undivided. The centrodiapophyseal fossa is deeper than in D4 and exhibits a large foramen within its ventral portion (Fig 9B). Both anterior and posterior centrodiapophyseal laminae merge proximally and continue distally as a single lamina (Fig 9B). The paradiapophyseal lamina is short and undivided (Fig 9B). The prespinal lamina is not preserved and, at present, there is no evidence of a spinoprezygapophyseal lamina. The prespinal fossa is deep and broad. An accessory anterior and spinodiapophyseal lamina proper is present on the left side and the former terminates laterally about midway along the length of the transverse process (Fig 9C). Both spinodiapophyseal lamina are at a low angle anteroventrally from the base of the neural spine to the diapophysis. The location and orientation of the accessory spinodiapophyseal lamina with relation to the spinodiapophyseal lamina proper is similar to the fifth dorsal vertebra of *Trigonosaurus pricei* [68], but differs from the parallel and close association of these two laminae in other titanosaurians such as *A*. *huinculensis* and *S*. *loricatus* [69]. The short spinopostzygapophyseal lamina appears to merge with the posterior spinodiapophyseal lamina near the base of the neural spine (Fig 9B). The junction of the spinodiapophyseal and spinopostzygapophyseal laminae is located laterally off the neural spine and courses towards the apex. The neural spine is transversely constricted and is angled significantly posteriorly more so than the other recovered dorsal neural arches. This drastic inclination of the neural spine at roughly 30 degrees to the horizontal is similar to some titanosaurians such as *Rinconsaurus caudamirus* from the Late Cretaceous of Argentina [70], *Paludititan nalatzensis* from the Late Cretaceous of Romania [71], and *T*. *pricei* from the Late Cretaceous of Brazil [68].

#### Dorsal vertebrae 6 and 7

**Fig 10:**
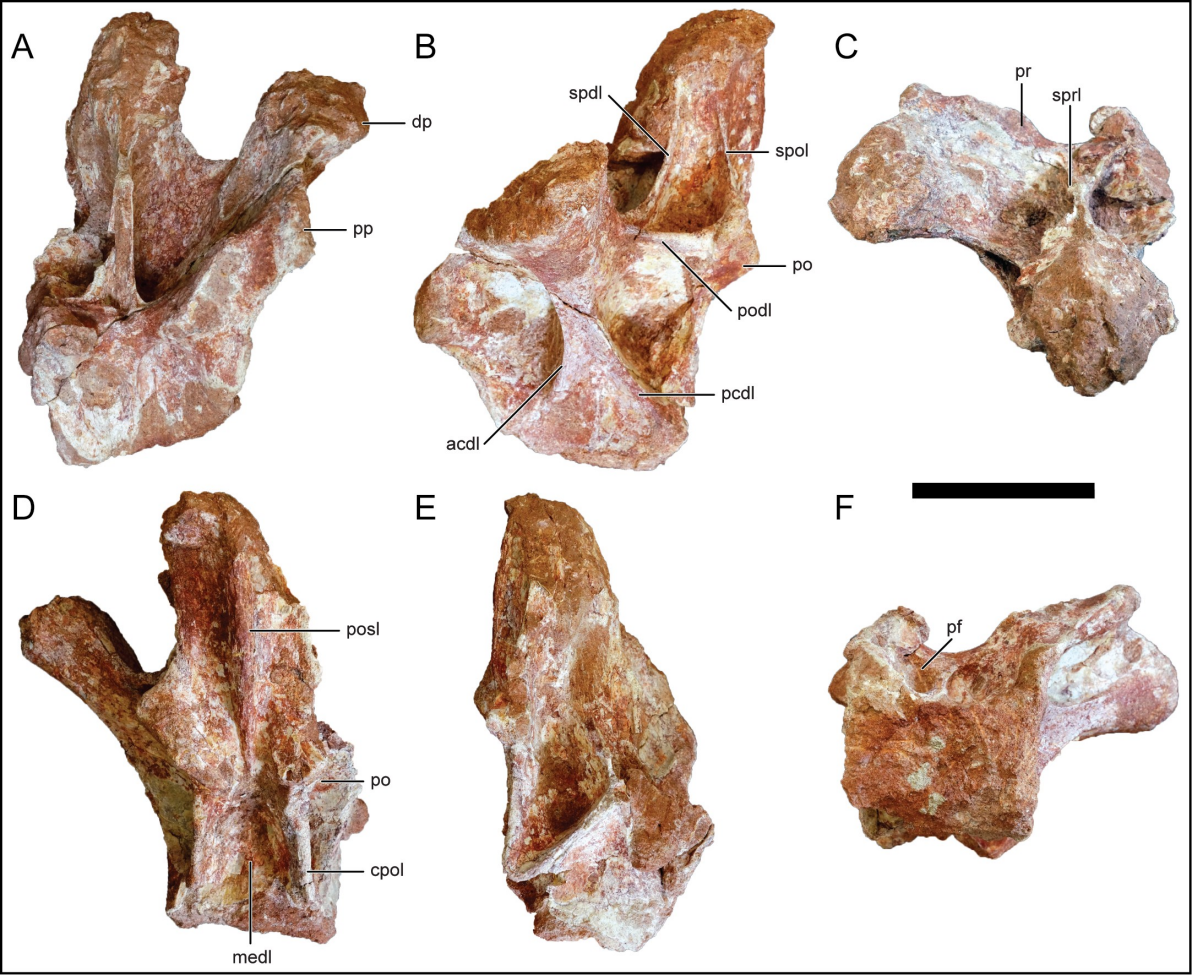
Posterior dorsal neural arch, D6, of *Mnyamawamtuka moyowamkia*. A, anterior; B, left lateral; C, dorsal; D, posterior; E, right lateral; and F, ventral views. Anterior towards the top of the page in C and F. Abbreviations: acdl, anterior centrodiapophyseal lamina; cpol, centropostzygapophyseal lamina; dp, diapophysis; medl, medial lamina; pcdl, posterior centrodiapophyseal lamina; po, postzygapophysis; podl, postzygodiapophyseal lamina; posl, postspinal lamina; pp, parapophysis; pr, prezygapophysis; spdl, spinodiapophyseal lamina; spol, spinopostzygapophyseal lamina; sprl, spinoprezygapophyseal lamina. Scale bar equals 10 cm.

**Fig 11:**
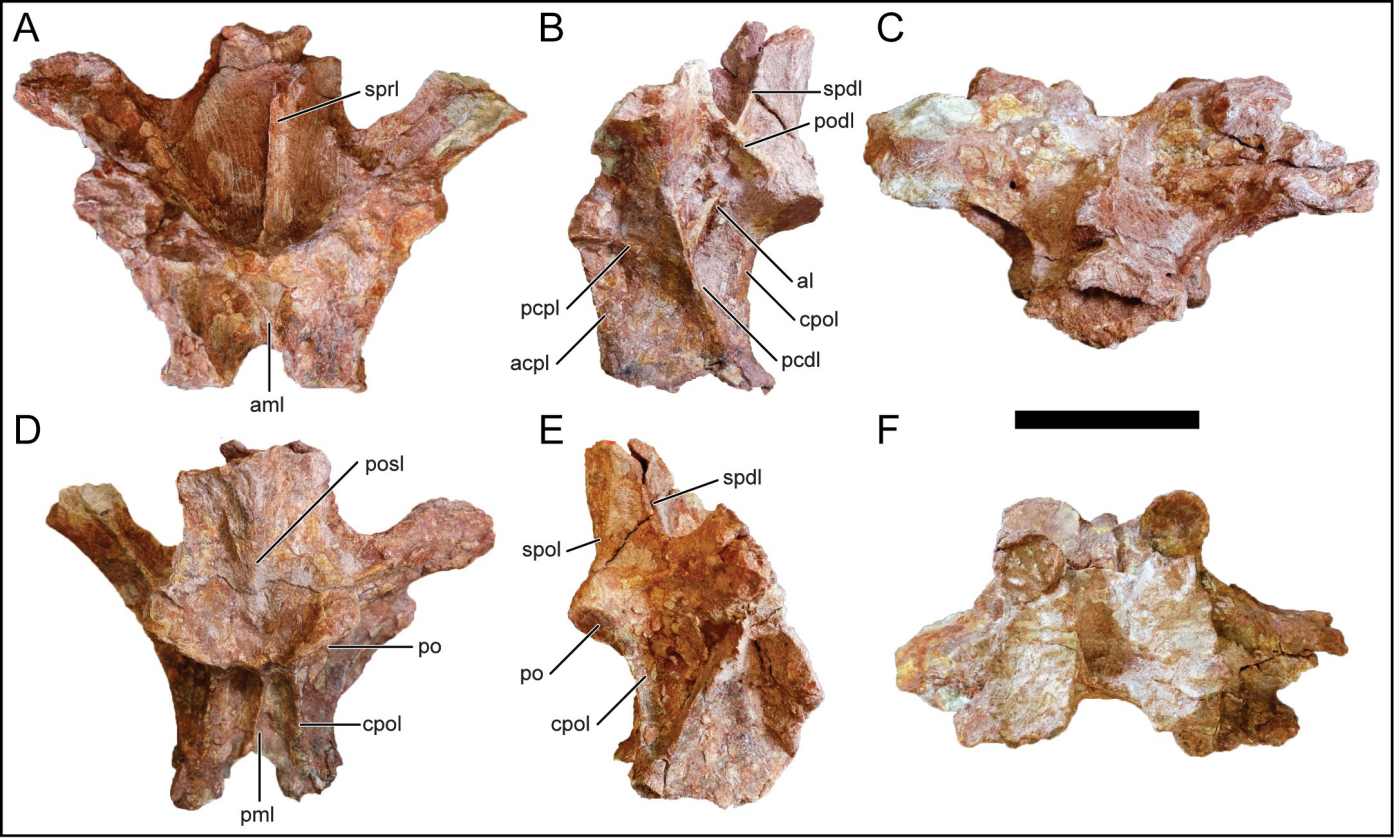
Posterior dorsal neural arch, D7, of *Mnyamawamtuka moyowamkia*. A, anterior; B, left lateral; C, dorsal; D, posterior; E, right lateral; and F, ventral views. Anterior towards the top of the page in C and F. Abbreviations: acpl, anterior centroparapophyseal lamina; al, accessary lamina; aml, anterior medial lamina; pcdl, posterior centrodiapophyseal lamina; cpol centropostzygapophyseal lamina; pcpl, posterior centroparapophyseal lamina; pml, posterior medial lamina; po, postzygapophysis; podl, postzygodiapophyseal lamina; posl, postspinal lamina; spdl, spinodiapophyseal lamina; spol, spinopostzygapophyseal lamina; sprl, spinoprezygapophyseal lamina. Scale bar equals 10 cm.

D6 (Fig 10) and D7 (Fig 11) are represented by nearly complete posterior dorsal neural arches. Both D6 and D7 are similar to one another, with the description predominantly based on D6 unless stated otherwise. The neural arch is relatively taller than the rest of the dorsal vertebrae. The parapophysis is partially preserved in both D6 and D7 and is located dorsolateral relative to the prezygapophysis. The transverse process is markedly inclined dorsolaterally (roughly 70 degrees from the horizontal) more so than the other dorsal vertebrae (Fig 10A). The prezygapophyseal facet faces dorsomedially with the elliptical postzygapophyseal facet facing ventrolaterally. The neural spine is oriented vertically in both D6 (Fig 10B) and D7 (Fig 11B and 11E), as expected in this region of the vertebral column as it transitions into the sacral vertebral series. The neural spine is transversely expanded, differing from the narrow condition in the more anterior dorsal vertebrae. The apices of the neural spines in both D6 and D7 are partially eroded.

The undivided centrodiapophyseal lamina in D7 is oriented anterodorsally and midway along the left centrodiapophyseal lamina, the centroparapophyseal and an accessory lamina branch from the centrodiapophyseal lamina (Fig 11B). The latter lamina is oriented in the same direction as the spinodiapophyseal lamina. However, this set of intercepting laminae is absent in D6 as the centrodiapophyseal lamina is wide with conjoined anterior and posterior laminae (Fig 10B). The centroprezygapophyseal lamina is undivided and faces anterolaterally. On both D6 and D7, there is a median lamina that courses from the dorsal margin of the neural canal and splits dorsolaterally as it reaches the ventral margin of the intraprezygapophyseal lamina (Fig 11A). This latter character is here considered an autapomorphy in *M*. *moyowamkia*. A similar lamina is seen in the Malagasy *R*. *krausei* [55]; however, this structure in *R*. *krausei* is undivided and weakly developed when compared to *M*. *moyowamkia*. The undivided centropostzygapophyseal lamina is tall (Fig 11D). The presence of the median lamina that connects the dorsal margin of the neural canal and interpostzygapophyseal lamina is similar to the anterior dorsal vertebrae. However in D6, the interpostzygapophyseal lamina is not present as the postspinal lamina and the median lamina is continuous, although the latter is reduced to a ridge in *M*. *moyowamkia* and considered an autapomorphy (Fig 10D). There is some ambiguity concerning the prespinal and the spinoprezygopophyseal laminae based on the quality of preservation. Two distinct laminae are present near the base (a left and a right) but it is unclear if it is the bilateral spinoprezygopophyseal laminae that are in close association or if the prespinal lamina is bifurcated at the base of the fossa (Fig 11A). A bifurcated prespinal lamina in the dorsal vertebrae is common amongst non-lithostrotian titanosauriformes [29]. The postspinal lamina in D7 is robust and represented by a low ridge within a shallow postspinal fossa (Fig 11D). The postzygodiapophyseal lamina is present in D6 and D7, differing from the derived condition of its absence in certain titanosaurians from the Late Cretaceous of Laurasia (e.g., *O*. *skarzynskii* [63]; *Lirainosaurus astibiae* [72]). The spinodiapophyseal lamina is undivided and is dorsally oriented (Fig 11B).

### Sacral vertebrae

**Fig 12:**
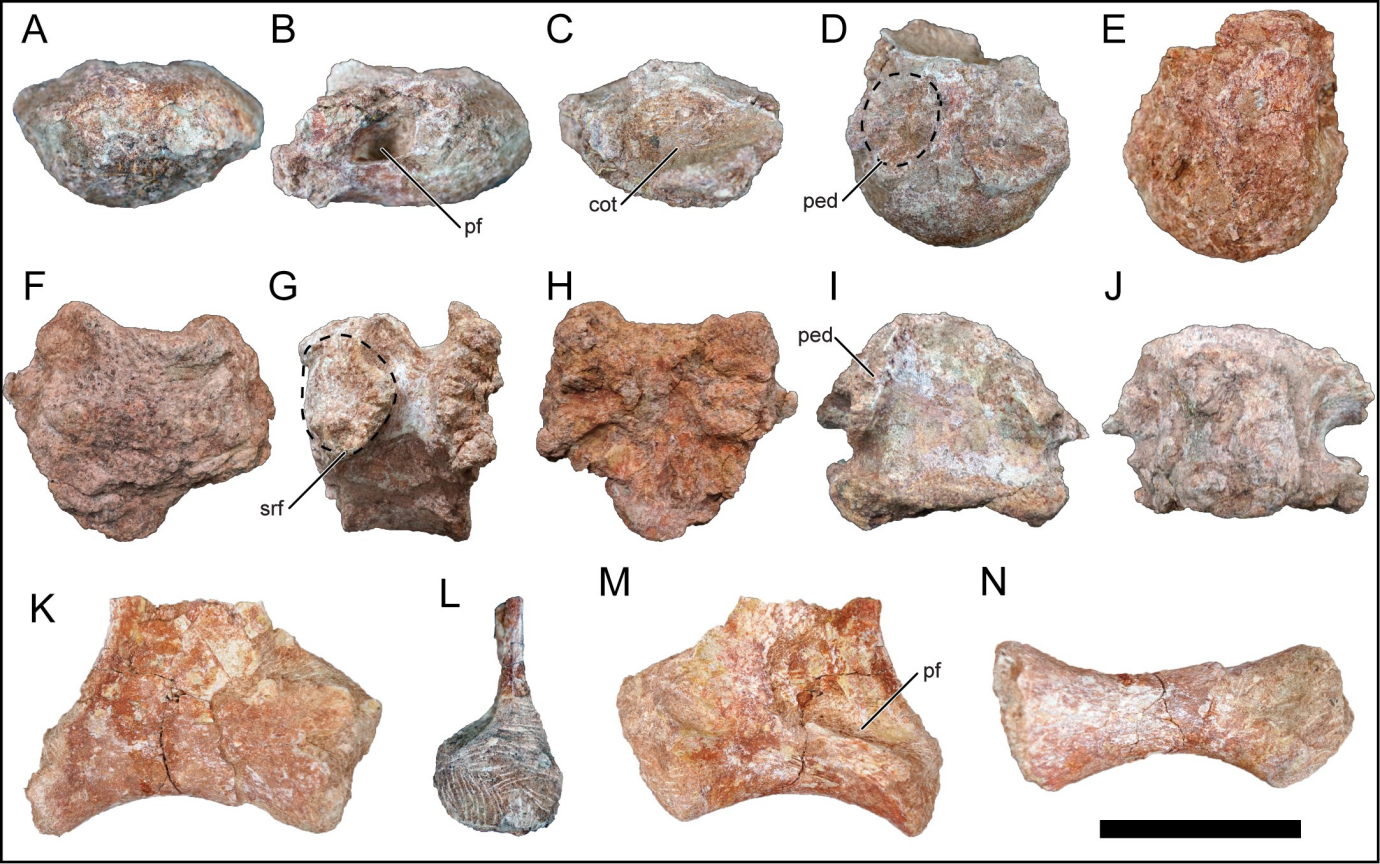
Sacral elements of *Mnyamawamtuka moyowamkia*. A–E, anterior sacral centrum; F–J, middle sacral centrum; and K–N, right sacral rib. A, F, M, anterior; B, G, left lateral; C, H, K, posterior; D, I, dorsal; E, J, N, ventral; and L, medial views. Anterior towards the top of the page in D, E, I, J, and N. Abbreviations: cot, cotyle; ped, site for fusion with pedicle; pf, pneumatic fossa; srf, sacral rib facet. Scale bar equals 10 cm.

Four partial sacral centra and three sacral ribs were recovered (Fig 12). It is currently unknown exactly how these elements relate to one another in position due to the state of preservation (e.g., if a specific sacral rib pertains to a specific sacral centrum and lack of neural arches). Some of the recovered centra exhibit camellate internal texturing along erosional surfaces (e.g., the first sacral centrum), a trait that is variably expressed in sacral vertebrae of titanosauriformes (33). The first sacral centrum is opisthocoelous (Fig 12A–12E) whereas the other sacral centra are amphiplatyan (Fig 12F–12J). In *R*. *krausei* and other titanosaurians including *Epachthosaurus sciuttoi* [73] and *Gondwanatitan faustoi* [74], the sacral centra progress anteroposteriorly from opisthocoelous–amphiplaty–procoelous conditions [55], essentially transitioning from opisthocoelous pre-sacral vertebrae to procoelous caudal vertebrae. In certain titanosaurians (e.g., *I*. *colberti* [60]; *Futalognkosaurus dukei* [75, 76]; *Overosaurus paradasorum* [77]), the articulation pattern within the sacrum is difficult to determine due to higher degrees of sacral vertebral fusion.

The anterior sacral centrum is dorsoventrally compressed with a smooth ventral surface whereas the other sacral vertebrae are dorsoventrally taller with a transversely compressed and keeled ventral surface (Fig 12H) (Table 1). An oval pneumatic fossa is present on both lateral surfaces near the margin of the cotyle on the first sacral vertebra (Fig 12B). The dorsal surface exhibits two enlarged subcircular areas for fusion with the sacral neural arch (Fig 12D). The remaining sacral centra are similar to the second and fourth sacral centra that were recovered for *R*. *krausei* [55], though the exact number of sacral vertebrae in *M*. *moyowamkia* is unknown. Titanosaurians typically exhibit six sacral vertebrae whereas the saltasaurid *Neuquensaurus australis* may have exhibited seven sacral vertebrae with the seventh being a transitional caudosacral vertebra [78]. The neural arch facets are placed within the anterior half of the centrum as are the facets for the sacral ribs. No pneumatic foramen is confidently identified on any centrum posterior to the first sacral.

Three right sacral ribs were recovered and are partially preserved. All three preserve the ventral portion of the element that articulates with the ilium and sacral centrum but not the dorsal portion that articulates with the neural arch. All three exhibit a similar morphology and will be described together unless noted otherwise (Fig 12K–12N). The rib is ventrally concave and the articular ends are widened relative to mid-length. In one of the sacral ribs, a shallow fossa is present on the anteromedial surface of the element (Fig 12M). The preserved dorsal edge of the sacral rib is a flat sheet of bone. The sheet is continuous with the posterior margin of the rib and is concave anteriorly onto the ventral portion of the rib. The dorsal portion of the bony sheet is limited to the medial half of the element. Overall, the sacral ribs of *M*. *moyowamkia* a similar with those preserved in *R*. *krausei* [55].

### Caudal vertebrae

**Fig 13:**
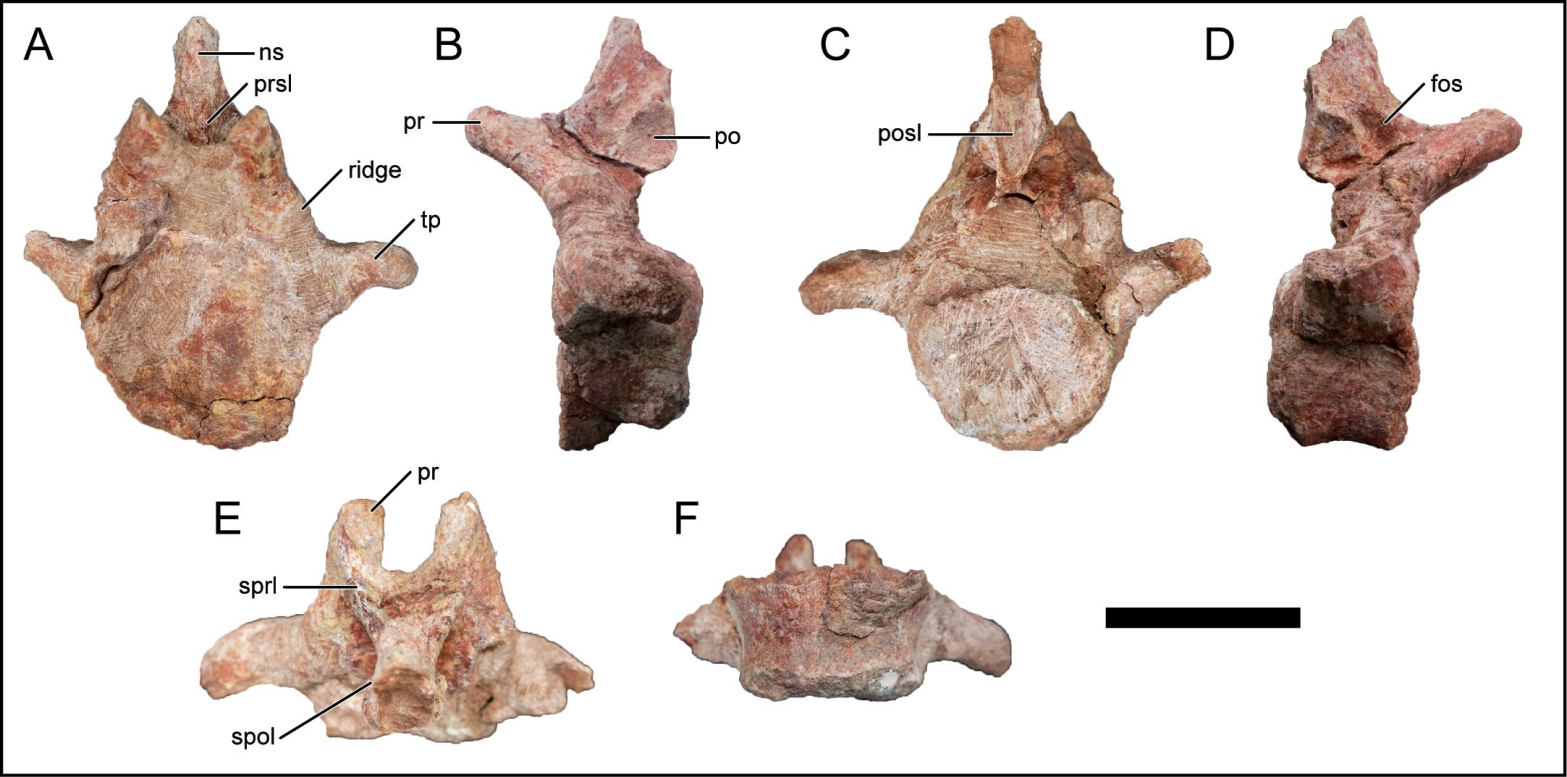
Anterior caudal vertebra of *Mnyamawamtuka moyowamkia*. A, anterior; B, left lateral; C, posterior; D, right lateral; E, dorsal; and F, ventral views. Anterior towards the top of page in E and F. Abbreviations: fos, fossa anterior to postzygapophysis; ns, neural spine; po, postzygapophysis; posl, postspinal lamina; pr, prezygapophysis; prsl, prespinal lamina; ridge, ridge along pedicle; spol, spinopostzygapophyseal lamina; sprl, spinoprezygapophyseal lamina; tp, transverse process. Scale bar equals 10 cm.

**Fig 14:**
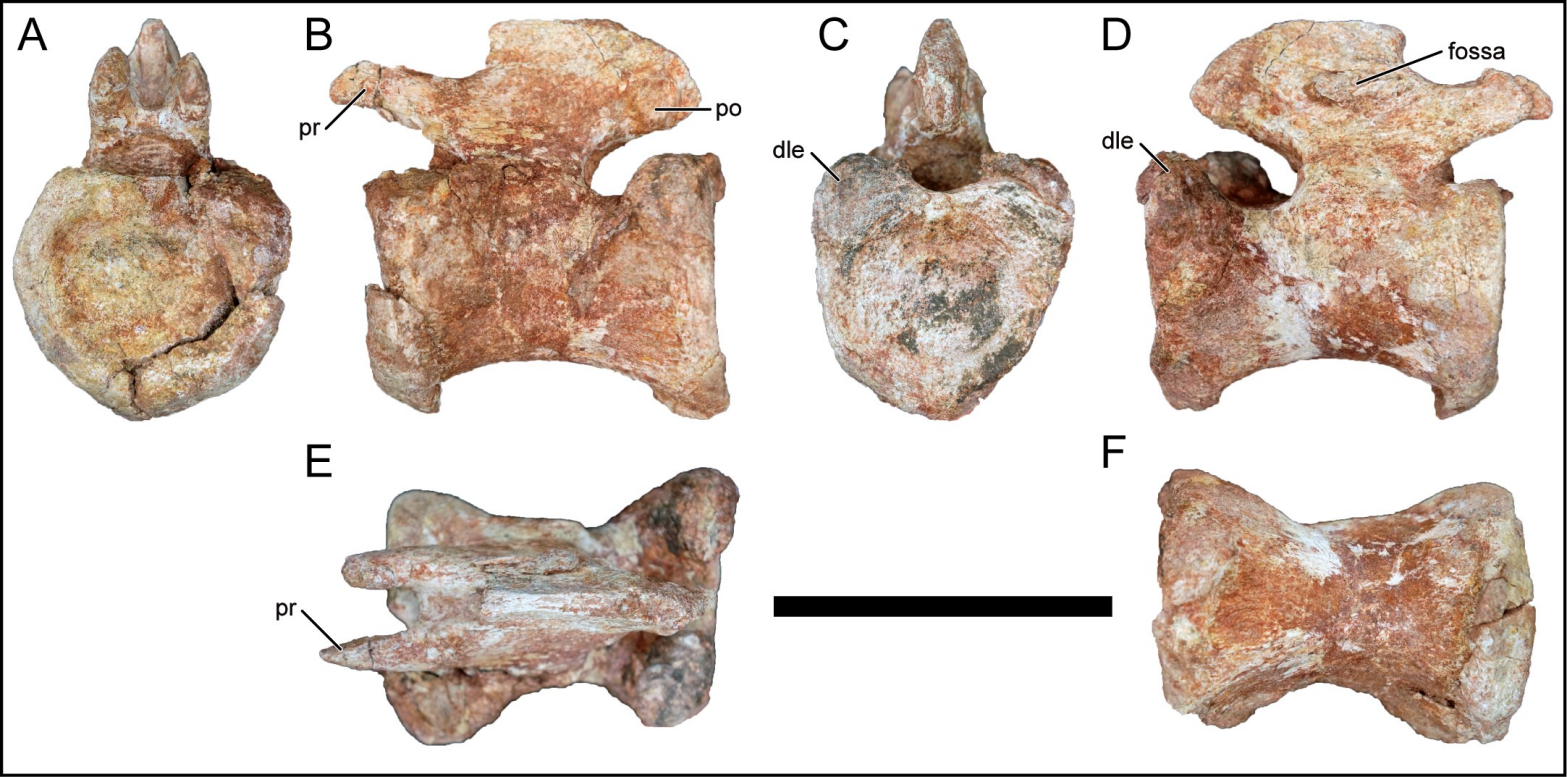
Middle-posterior caudal vertebra of *Mnyamawamtuka moyowamkia*. A, anterior; B, left lateral; C, posterior; D, right lateral; E, dorsal; and F, ventral views. Anterior towards the left in E and towards the right in F. Abbreviations: dle, dorsolateral expansion; fossa, unnamed neural arch fossa; po, postzygapophysis; pr, prezygapophysis. Scale bar equals 10 cm.

**Fig 15:**
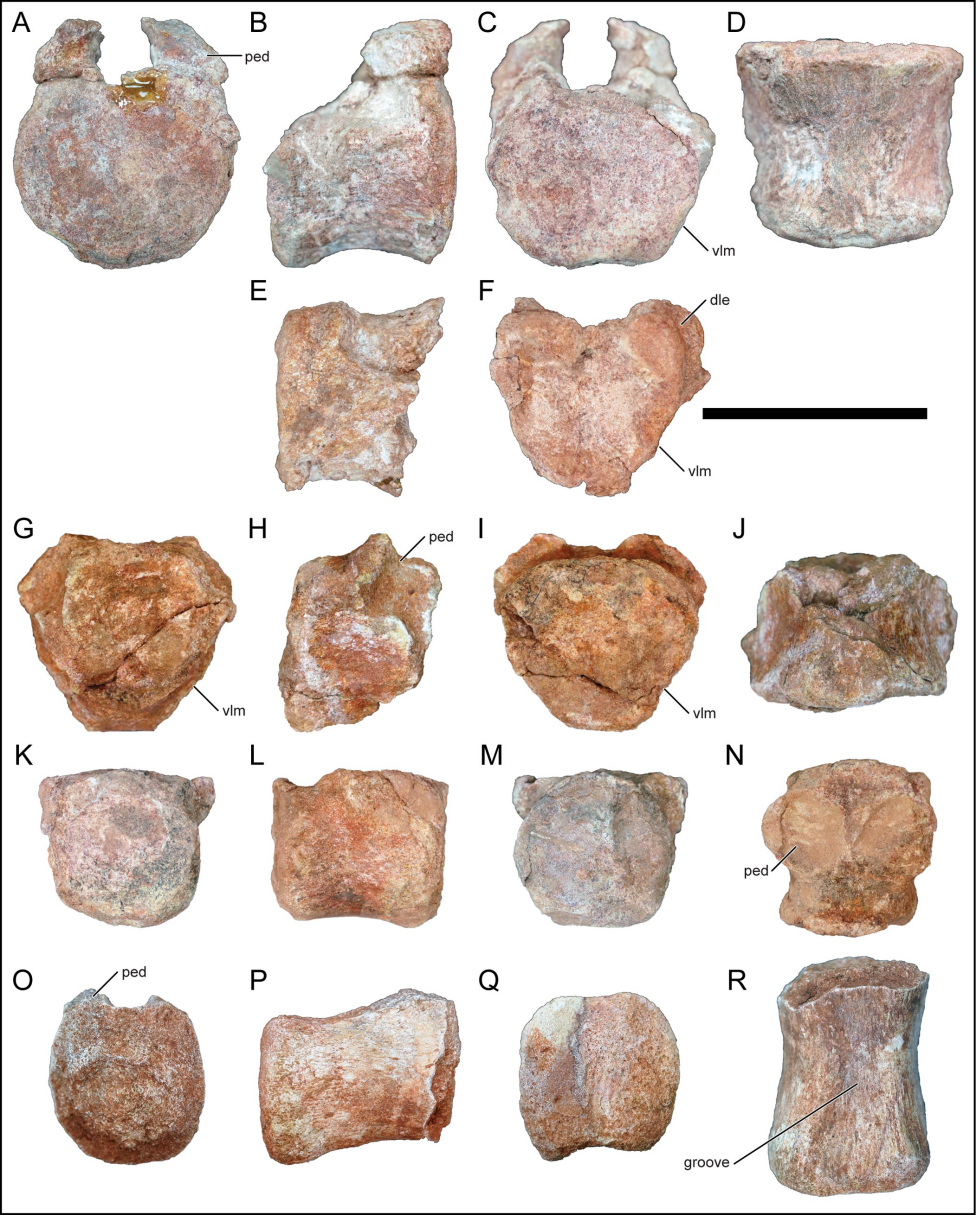
Middle and posterior caudal centra of *Mnyamawamtuka moyowamkia*. A–D and E–F, middle caudal centra, G–J and K–N, middle–distal caudal centra, and O–R, posterior caudal centrum. A, G, K, O, anterior; B, E, H, L, P, right lateral; C, F, I, M, Q, posterior; D, J, R, ventral; and N, dorsal views. Anterior towards the top in D, J, N, and R. Abbreviations: dle, dorsolateral expansion; groove, ventral groove; ped, pedicle; vlm, ventrolateral margin. Scale bar equals 10 cm.

**Fig 16:**
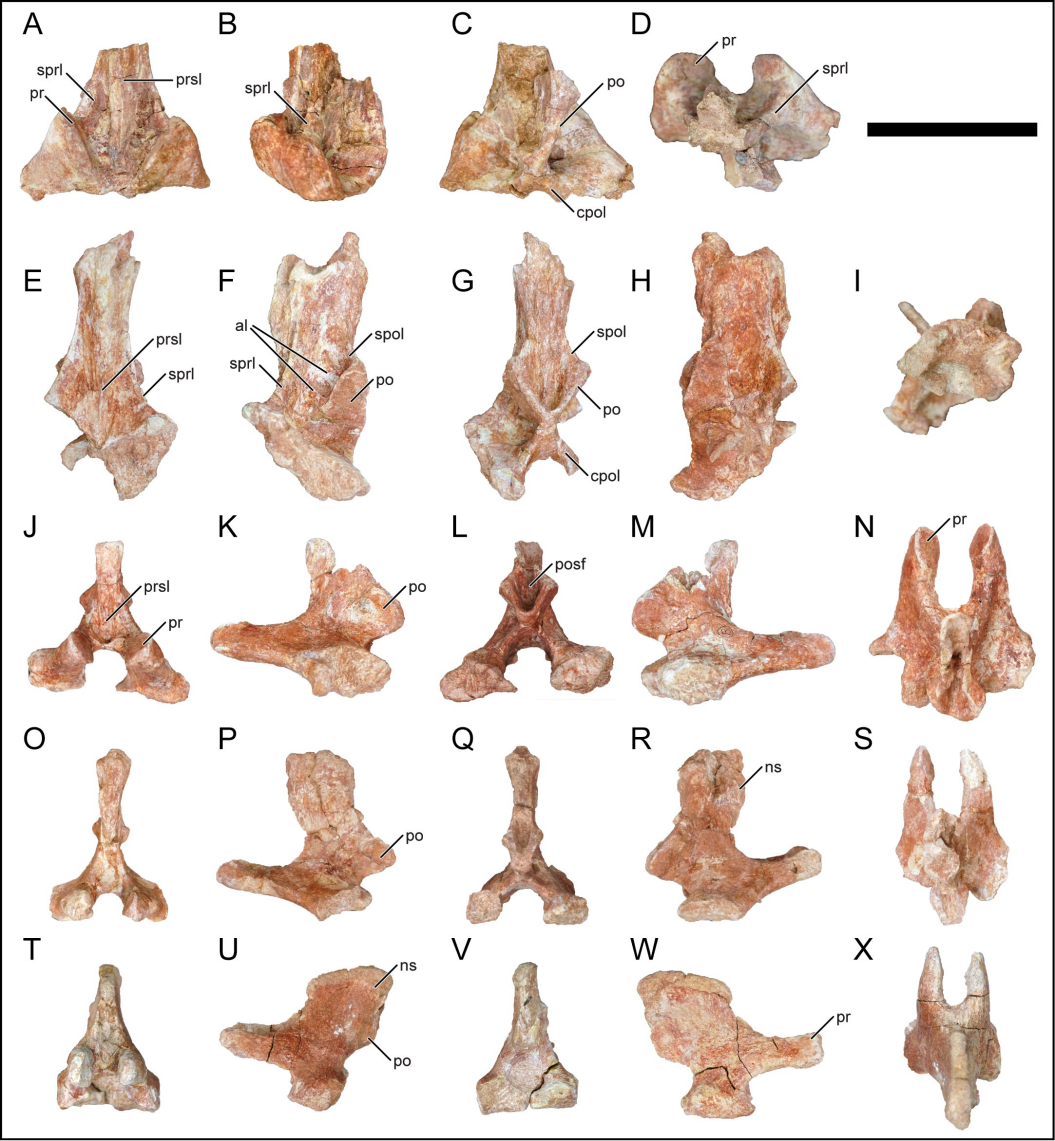
Caudal neural arches of *Mnyamawamtuka moyowamkia*. A–D, First caudal neural arch, E–I, anterior caudal neural arch, J–N and O–S, middle caudal neural arches, and T–X, posterior caudal neural arch. A, E, J, O, T, anterior; B, F, K, P, U, left lateral; C, G, L, Q, V, posterior; D, H, M, R, W, right lateral; and I, N, S, X dorsal views. Anterior towards the top in D, I, N, S, X. Abbreviations: al, accessary lamina; cpol, centropostzygapophyseal lamina; ns, neural spine; po, postzygapophysis; posf, postspinal fossa; pr, prezygapophysis; prsl, prespinal lamina; spol, spinopostzygapophyseal laminal; sprl, spinoprezygapophyseal lamina. Scale bar equals 10 cm.

The caudal vertebral skeleton is represented by a nearly complete anterior caudal (Fig 13), a middle-posterior caudal (Fig 14), and several variably preserved caudal centra (Fig 15) and neural arches (Fig 16). There are no signs of pneumatic features, external or internal, in any of the recovered caudal vertebrae. Besides the nearly complete anterior and middle-posterior caudal vertebrae, the recovered centra and neural arches were not completely fused to their respective counterpart nor were there any transverse processes recovered. The lack of fully or even partially fused caudal vertebrae, coupled with the lack of fusion in the cervical, dorsal, and sacral vertebrae, further suggests that *M*. *moyowamkia* had yet to attain skeletal maturity. For example, three middle–posterior caudal centra were recovered and exhibit large circular pits that act as suture sites with the neural arch. The order of fusion of the vertebral column for titanosaurians, let alone sauropods in general, is hardly understood and likely to be variable in the progression of ossification [79].

#### Anterior caudal vertebrae

The nearly complete anterior caudal vertebra is moderately preserved, with some erosion along the centrum and neural spine (Fig 13). The anterior centrum face is slightly concave and the posterior centrum face is eroded but looks to have been slightly convex given its preservational state. In *M*. *dixeyi*, *R*. *bisepultus*, *and Traukutitan eocaudata*, and to an extent in *Andesaurus delgadoi*, the anterior-most caudal vertebrae exhibit a strongly procoelous condition that transitions to an amphiplatyan condition in the remainder of the caudal series [3, 11, 80, 81]. The anterior caudal vertebra of *M*. *moyowamkia* is either from the posterior-most region of the anterior caudal series or exhibits a condition of mild procoely within the anterior caudal series as in the titanosaurian *A*. *delgadoi* and some non-titanosaurian titanosauriforms [29, 33, 81]. The exact pattern of procoely (i.e., mild procoely throughout the anterior caudal series vs. mild procoely due to the serial position within the posterior-most anterior caudal series) is unknown as no other positively identified anterior caudal centra have been recovered from the quarry. The centrum is subequal in height and width (Table 1). The ventral surface is generally smooth and moderately concave along the anteroposterior axis. There is no ventral longitudinal groove, ventrolateral ridge, or articular facet for the haemal arch (Fig 13F).

The transverse process projects laterally from the dorsal margin of the centrum and gently curves posteriorly but not past the posterior margin of the centrum as is the case in *A*. *delgadoi* and many other titanosaurians [33, 81]. The neural arch is attached to the anterior half of the centrum, as typical in titanosauriformes [29, 33, 48]. Moreover, the anterior margin of the neural arch is located at the anterior margin of the centrum. The neural canal is subcircular. A subtle longitudinal ridge is present on the lateral surface of the pedicle (Fig 13A). A more pronounced ridge is present in the Late Cretaceous titanosaurian *Baurutitan britoi* from the Late Cretaceous of Brazil [82], *R*. *bisepultus* [11], and is variably developed within titanosauriformes [29]. The prezygapophysis projects anterodorsally and the flat articular facet faces dorsomedially. The spinoprezygapophyseal lamina lacks the subtle tubercle on the dorsal surface as seen in the euhelopodids *Phuwiangosaurus sirindhornae* and *Tangvayosaurus hoffeti*, the brachiosaurid *Giraffatitan brancai*, *R*. *bisepultus*, and saltasaurid titanosaurians more generally [11, 29]. The elliptical and slightly concave postzygapophysis faces ventrolaterally. There is a weakly developed fossa on the lateral surface of the neural spine anterior to the postzygapophysis (Fig 13D), a feature that is present in the titanosaurians *R*. *bisepultus* [11] and *R*, *krausei* [55]. The neural spine is oriented mostly vertical. A prespinal lamina is present within the prespinal fossa whereas the postspinal lamina is less well developed within its respective postspinal fossa. The spinoprezygapophyseal lamina is reduced. The short spinopostzygapophyseal lamina buttresses the dorsal margin of the postzygapophysis (Fig 13E).

#### Middle–posterior caudal vertebra

**Fig 17:**
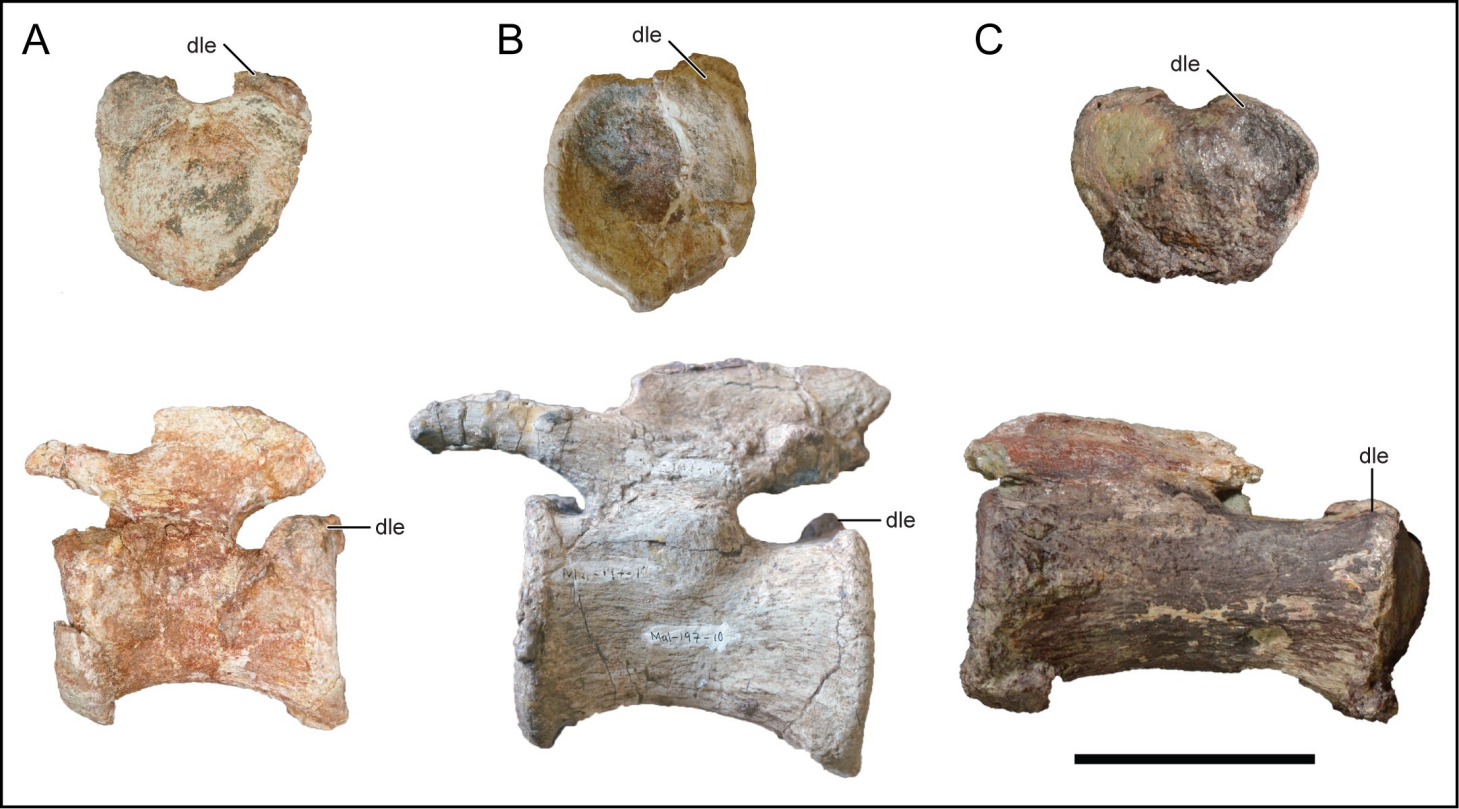
Comparison of caudal vertebrae of *Mnyamawamtuka moyowamkia*. *Mnyamawamtuka moyowamkia*, A, *Malawisaurus dixeyi* MAL-197-8, B, and *Lohuecotitan pandafilandi*, C, in posterior view. Abbreviations: dle, dorsolateral expansion. Scale bar equals 10 cm.

A complete middle–posterior caudal vertebra mentioned above was recovered and exhibits minimal erosional damage and distortion (Fig 14). The centrum is generally cylindrical with the ventral surface constricted transversely as in some of the other caudal vertebrae (Fig 14C). The anterior articular surface is slightly concave with a noticeable circumferential brim. The posterior articular surface is similar but is less concave and nearly flat. The rim of the posterior centrum exhibits a dorsolateral rounded expansion and ventral narrowing, conferring a heart-shaped outline (Fig 16C). The well-defined dorsolateral expansion of the posterior centrum articular surface is considered an autapomorphy for *M*. *moyowamkia*. In three middle caudal vertebrae attributed to *M*. *dixeyi* (MAL-197-8–10; Gorscak, pers. obvs., 2014) and distal caudal vertebrae of *Lohuecotitan pandafilandi* [83], a similar feature is present but is much less developed when compared to *M*. *moyowamkia* (Fig 17). In MAL-197-8 (Fig 17B), the subtle dorsolateral expansion is only present on the right side and in Mal-197-9–10 the expansion is minimally present as a low bump as is the condition in the distal caudal vertebra of *L*. *pandafilandi* (Fig 17C). The reduced and short prezygapophysis is oriented mostly horizontally and the articular facet faces medially. The small postzygapophysis is subcircular, located near the posterior margin of the neural spine, and faces laterally. The short neural spine is compressed transversely. The right lateral surface of the neural spine exhibits a shallow elliptical fossa (Fig 14D).

#### Caudal centra

Several caudal centra were recovered (Fig 15). All the recovered centra are slightly amphiplatyan/amphicoelous. The middle–posterior caudal centra are generally box-like (Fig 15K–15R), whereas some of the anterior–middle caudal centra tend to exhibit transversely compressed ventrolateral margins (Fig 15A–15J). The latter condition is similar to the non-titanosaurian titanosauriformes *Wintonotitan wattsi* from the Cenomanian of Australia [84] and *C*. *insignis* from the Cenomanian of Argentina [67], and some titanosaurians such as *G*. *faustoi* [74] and *N*. *australis* [78]. The ventral surface of the centrum is mildly concave and does not exhibit a ventrolateral ridge or posterior haemal arch articular facet; however, the recovered distal caudal centrum exhibits a faint longitudinal groove along the ventral surface (Fig 15R). One of the middle caudal centra exhibits a unique dorsolateral expansion of the posterior articular surface of the centrum, which is present in the mostly intact middle caudal vertebra described above (Fig 15F). The neural arch is located within the anterior half of the centrum, a trait of titanosauriformes [48]. The suture area for the neural arch on the centrum is an enlarged circular pit (Fig 15N). The lengths of the centra are generally consistent except for the distal centrum that is relatively elongate (Table 1).

#### Caudal neural arches

Seven caudal vertebral neural arches were recovered, representing the first, anterior, anterior–middle, and middle–posterior portions of the caudal region (Fig 16). The neural arches are described with the neural canal oriented horizontally. The first anterior caudal neural arch preserves part of the zygapophyseal region and the base of the neural spine (Fig 16A–16D). The prezygapophysis and postzygapophysis are obliquely oriented with strongly elliptical facets. The prezygapophysis does not extend anteriorly as is the case in the first caudal vertebra of the Late Cretaceous titanosaurians *Alamosaurus sanjuanensis* ([85]; E.G. pers. obvs., 2014), *B*. *britoi* [82], and *F*. *dukei* [75, 76]. The prespinal and spinoprezygapophyseal laminae are strongly developed as expected in the first and second caudal neural arch (Fig 16A). The postspinal fossa is bounded ventrally by the bilateral postzygapophyses and centropostzygapophyseal laminae in a distinct X-shape (Fig 16C). The anterior-most neural arch exhibits both well-defined laminae and fossae but both prezygapophyses and the dorsal portion of the neural spine are not preserved (Fig 16E–16I). The elliptical postzygapophysis faces ventrolaterally and meets the centropostzygapophyseal lamina at the midline to form an X-shape as in the first caudal neural arch (Fig 16G). However, the postzygopophysis is flat and based on the prezygapophyseal morphology of the other neural arches, the caudal vertebrae did not exhibit the unique hypantrum-hyposphene complex that is present in the titanosaurian *E*. *sciuttoi* from the early Late Cretaceous of South America [73]. The neural spine is oriented mostly vertically and the lateral surface exhibits numerous fossae divided by several accessory laminae spanning the postzygapophysis and neural spine (Fig 16F). A similar condition is exhibited in the anterior caudal vertebrae of the titanosaurian *Bonatitan reigi* from the Late Cretaceous of Argentina [86]. The well-developed prespinal lamina tapers proximally within the prespinal fossa. The spinoprezygapophyseal and spinopostzygapophyseal laminae are well defined and the latter buttresses the dorsal portion of the postzygapophysis with the neural spine. The postspinal lamina is thin and within a shallow postspinal fossa.

Four neural arches from the anterior–middle caudal vertebral region were recovered and are described together due to their overall similarity (Fig 16J–16S). The elongate prezygapophysis extends anteriorly and is slightly deflected dorsally. The small articular facet faces dorsomedially and matches the oval shape of the postzygopophysis. The postzygapophysis faces mostly laterally and the long axis of the postzygapophysis is oriented at an acute angle with respect to vertical. Additionally, the ventral margin of the postzygapophysis is offset by extending past the posterior margin of the pedicle and neural spine (Fig 16K and 16P). This offset postzygopophyseal condition is exhibited in the Late Cretaceous South American aeolosaurid titanosaurians of *Aeolosaurus maximus* [87] and *T*. *pricei* [68], and is not as developed in the non-titanosaurian titanosauriform *Tastavinsaurus sanzi* from the Aptian of Spain [88]. The postzygapophyses and the centropostzygapophyseal laminae also form a distinctive X-shape, lacking a well-defined hyposphenal ridge exhibited by lithostrotian titanosaurians (33). The prespinal lamina is weakly developed and within a reduced prespinal fossa, similar to the postspinal lamina and fossa. The neural spine is oriented vertically and in two of the anterior–middle neural arches, the neural spine is slightly anterodorsally inclined. A vertical anteriorly-inclined caudal neural spine is typically exhibited by aeolosaurid titanosaurians (e.g., *A*. *maximus*, *G*. *faustoi*, *R*. *krausei* [21, 48, 87]), though a vertical neural spine is exhibited in several other titanosaurians (e.g., *A*. *sanjuanensis*, *Dreadnoughtus schrani*: E.G. pers. obvs., 2014).

The recovered middle–posterior caudal vertebral neural arch is well preserved (Fig 16T–16X). The horizontal prezygapophysis is shorter than the other caudal vertebral prezygapophyses. The elliptical postzygapophysis faces ventrolaterally and is located at the posterior end of the neural arch. The neural spine is transversely compressed, dorsoventrally short, and oriented posterodorsally.

### Dorsal ribs

**Fig 18:**
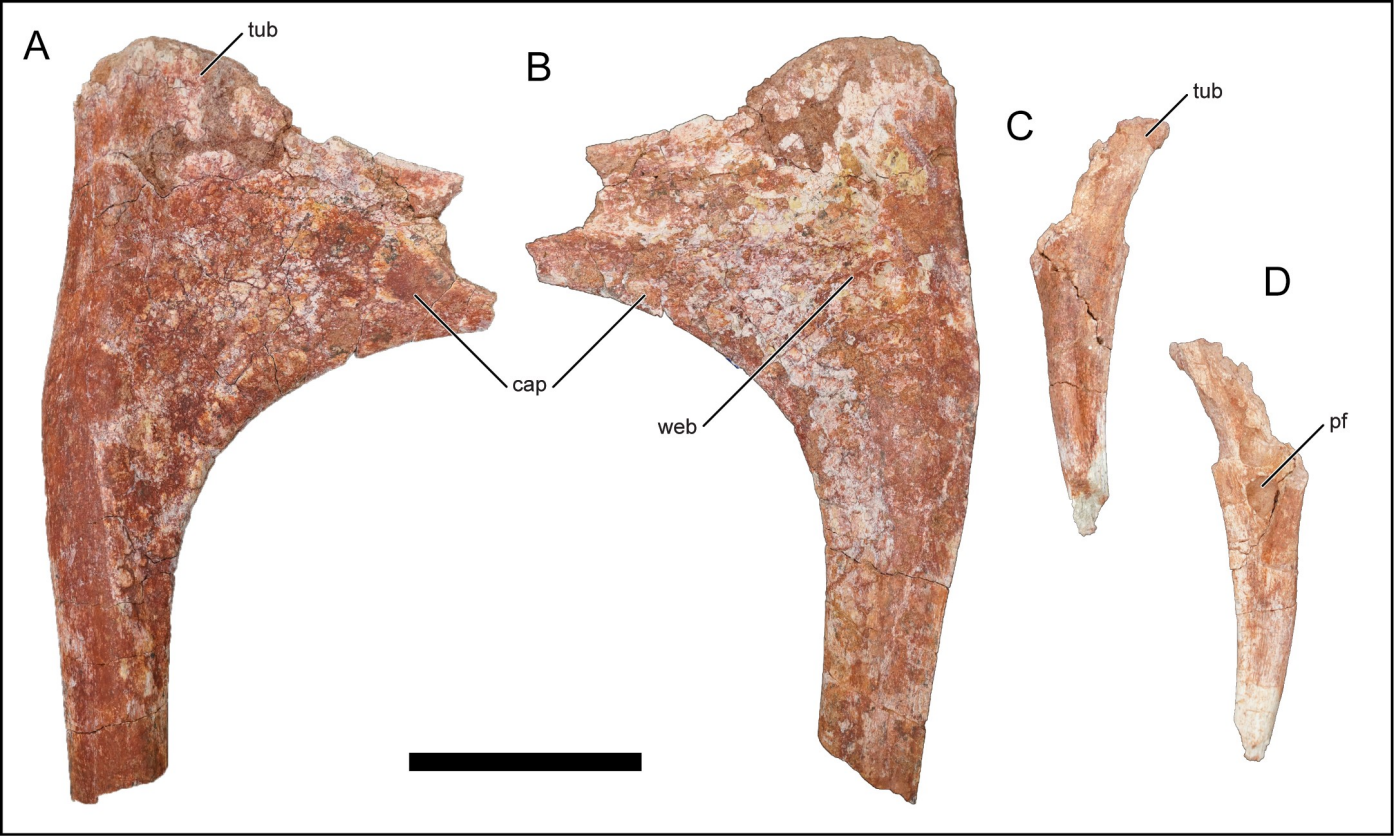
Dorsal ribs of *Mnyamawamtuka moyowamkia*. A–B, right anterior dorsal rib, and C–D, right posterior dorsal rib. A, C, anterior; and B, D, posterior views. Abbreviations: cap, capitulum; pf, pneumatic fossa; tub, tubercle; web, capitulotubercular webbing. Scale bar equals 10 cm.

Roughly 20 fragments of dorsal ribs were recovered with most representing pieces of rib shafts. The recovered rib shafts vary from a subcircular to transversely compressed (i.e., plank-like) cross sections that are typical of titanosauriforms [18]. One proximal anterior rib fragment exhibits a wide capitulotubercular region with weakly developed pneumatic webbing along the posterior surface (Fig 18B). A similar webbing morphology is exhibited in *R*. *bisepultus* although in *M*. *moyowamkia* this webbing is only weakly developed. The anterior rib capitulum is pronounced, elongated, and is widely separated from the low and rounded tuberculum. A proximal portion of a posterior dorsal rib exhibits a pneumatic fossa that pierces the posterior rib surface (Fig 18D), and is similar to a posterior dorsal rib recovered from *R*. *bisepultus*, some posterior dorsal ribs of *M*. *dixeyi*, and *D*. *matildae* from Australia. The recovered anterior dorsal ribs do not exhibit the characteristic anterior and posterior flanges that occur in the aeolosaurid titanosaurians *O*. *paradasorum* and *S*. *songwensis* [12, 77].

### Haemal arches

**Fig 19:**
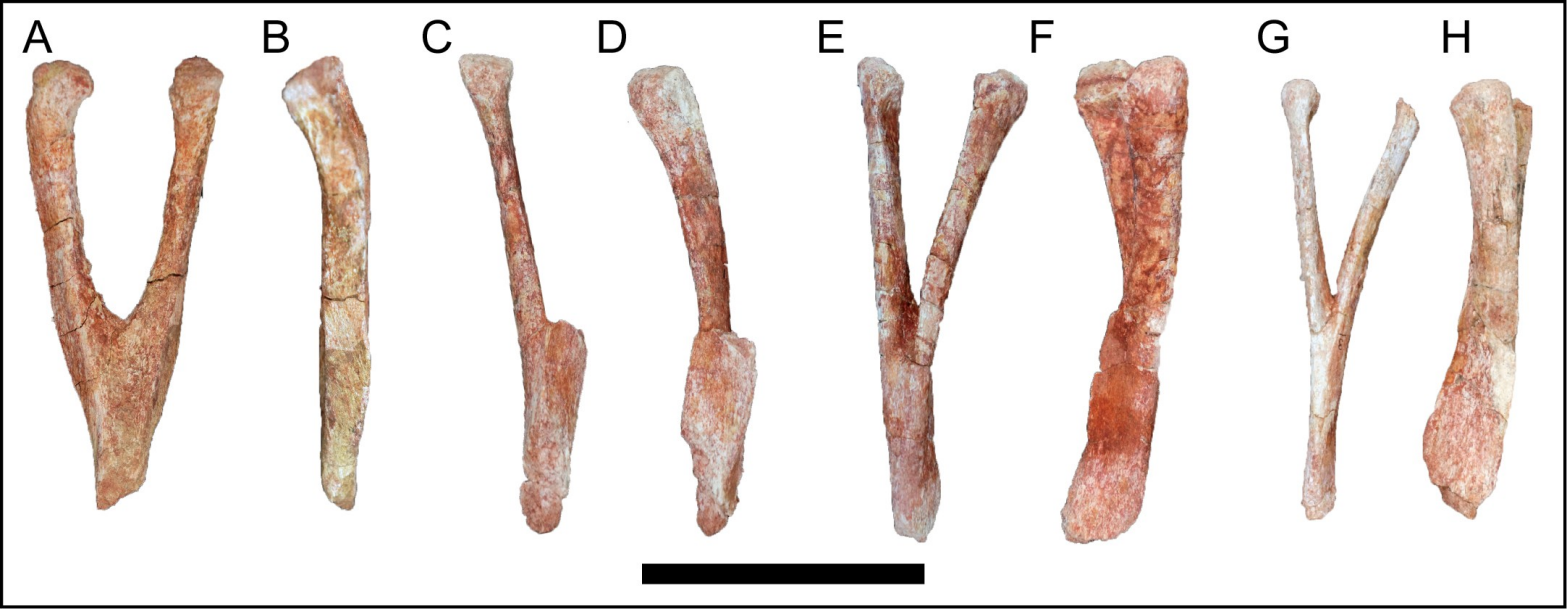
Haemal arches of *Mnyamawamtuka moyowamkia*. A–B, anterior haemal arch, and C–D, E–F, and G–H, middle haemal arches. A, C, E, G, anterior; and B, D, F, H, right lateral views. Scale bar equals 10 cm.

The four recovered haemal arches of *M*. *moyowamkia* do not significantly deviate from the typical titanosauriform condition (Fig 19). The depth of the haemal canal is at least 50% of the element length (Fig 19A, 19C, 19E and 19G). The articular facet is not divided as in euhelopodid titanosauriformes [29], nor is the facet doubled as in aeolosaurian titanosaurians [87] and some haemal arches attributed to *M*. *dixeyi (*Gorscak pers. obvs. 2014, 2015). The anterior-most and largest haemal arch does not preserve the distal blade and the proximal articular head is angled posteriorly off the arm of the haemal arch (Fig 19B).

### Scapula

**Fig 20:**
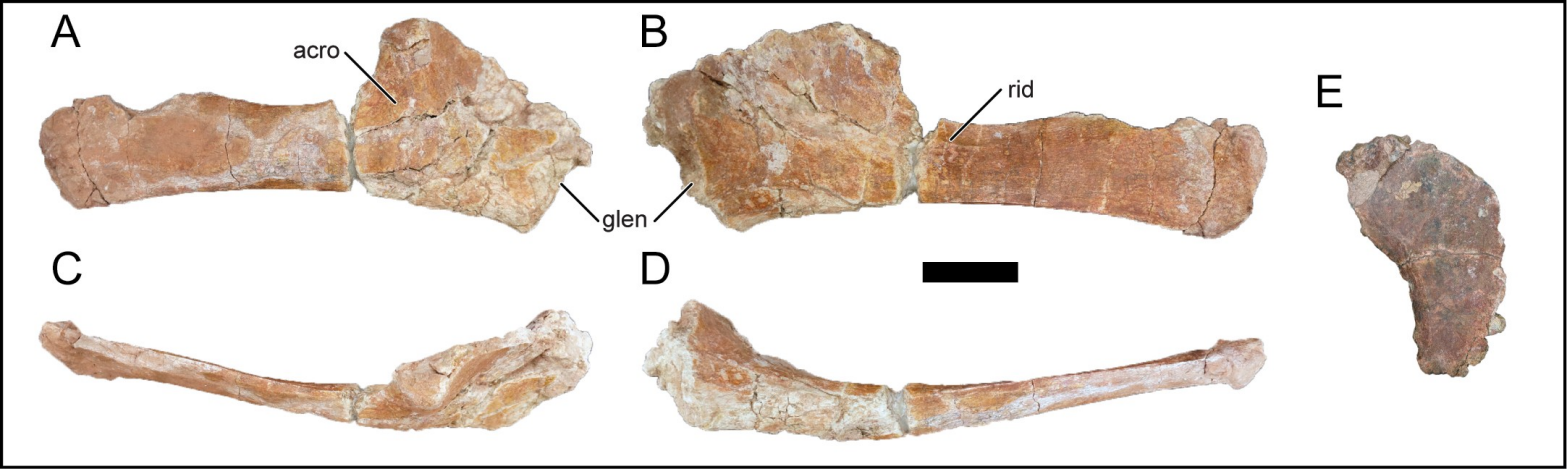
Pectoral elements of *Mnyamawamtuka moyowamkia*. A–D, right scapula, and E, right sternal plate. A, lateral; B, medial; C, dorsal; D, ventral; and E, external/ventral views. Abbreviations: acro, acromion; ant, anterior; art, articular facet for coracoid; glen, glenoid fossa; lat, lateral; med, medial; pos, posterior; rid, dorsomedial ridge of the scapular blade. Scale bar equals 10 cm.

A nearly complete right scapula was recovered; however, the anterodorsal portion, including articular surfaces for the coracoid and glenoid, is not preserved (Fig 20A–20D). The scapula will be described with the long axis oriented horizontally. The terminal end of the scapular blade is rugose on both medial and lateral surfaces. The scapular blade mildly expands at the distal end, more so along the dorsal than ventral margin (Table 2). This blade expansion occurs near mid-length and appears shelf-like along the dorsal margin whereas the ventral margin gradually expands. A shelf-like expansion of the scapular blade is variably expressed within titanosauriforms and includes forms such as *C*. *insignis* [67] and *A*. *adamastor* [89], and the titanosaurian *D*. *matildae* [64, 65]. *R*. *bisepultus* and *M*. *shahinae* both do not exhibit this dorsal shelf-like expansion, further distinguishing these African forms [11, 16]. Most of the blade is nearly flat in cross-section, which is typical in most titanosauriforms [29], but the blade is D-shaped proximally with an external convexity and internal concavity. There is no ventral process near the proximal end of the blade, a condition shared with *R*. *krausei* [55] and *L*. *astibiae* [90] and unlike its presence seen in the Egyptian titanosaurs *P*. *stromeri* [2] and *M*. *shahinae* [16]. The presence of a ventral process has been proposed to be a titanosauriform synapomorphy [29], being present in the non-titanosaurian titanosauriformes *A*. *adamastor* [89] and *C*. *insignis* [67], and most titanosaurians. Unique to *M*. *moyowamkia* is a curved ridge at the proximomedial region of the medial scapular blade (Fig 20B). A tubercle, rather than a ridge, is present at this location in several titanosaurians including *L*. *astibiae* [90], *M*. *shahinae* [16], *P*. *stromeri* [2], and *S*. *loricatus* [58]. The acromion rises dorsally from the scapular blade at a nearly perpendicular angle and is approximately twice the dorsoventral height of the proximal scapular blade. The acromion ridge runs anteroventrally from the posterodorsal corner to near the middle of the proximal region (Fig 20A). The glenoid faces anteroventrally yet the anterior margin is not well-preserved so interpretation on the degree of mediolateral orientation of the articular surface is unclear.

**Table 2:** Select measurements of *Mnyamawamtuka moyowamkia* appendicular skeleton.

	**TL**	**BL**	**PBH**	**DBH**	**AH**
**Scapula**	622	377	91	128	198
	**TL**	**PW**	**MW**	**DW**	
**Sternal Plate**	249	-	154	-	
**Humerus**	569*	215*	110	201	
**Radius**	-	-	-	59	
**Metacarpal I**	184	48	31	54	
**Metacarpal III**	236	51	37	67	
**Femur**	720*	214	119	193	
**Tibia**	474	117	43	139*	
**Fibula**	498	63	43	77	
**Metatarsal I**	95	73	50	61	
**Metatarsal II**	109	69	44	59	
**Metatarsal III**	129	75	31	51	
**Metatarsal IV**	122	31	21	33	
**Metatarsal V**	82	69	49	40	
**Pedal Phalanx**	51	47	44	-	
**Ungual I**	89	54	-	-	
**Ungual III/IV?**	48	33	-	-	

Abbreviations: AH, acromial portion of scapula dorsoventral height; BL, scapular blade length; DBH, distal scapular blade dorsoventral height; DW, distal width; MW, midpoint width; PBH, proximal scapular blade dorsoventral height; PW, proximal width; TL, total element length.

*denotes measurement of incomplete area of fossil. Measurements are in mm.

### Sternal plate

A right sternal plate was recovered and preserves much of the morphology except for minor damages along the edges (Fig 20E). The sternal plate is crescentic like most titanosauriformes [18, 29, 33, 48]. The sternal plate is thickest along the lateral border and mildly rugose along the medial margin for articulation with the contralateral sternal plate. The articular area for the coracoid is weakly developed and lacks an anterolateral ridge or projection that is present in most titanosaurians [72]. The ratio of the maximum length of the sternal plate to the recovered partial humerus does not meet the proposed threshold for lithostrotian titanosaurians (0.75 [91]; >0.70 [29]; >0.65 [33]), as present in *M*. *shahinae* [16], for example. Rather, the sternal plate is unusually small: the ratio with the partial humerus is at most 0.42 whereas non-lithostrotian titanosauriforms typically exhibit a ratio around 0.50–0.65 [29, 33, 91] (Table 2). The unusually small sternal plate when compared to the humerus is here considered an autapomorphy for *M*. *moyowamkia*.

### Humerus

**Fig 21:**
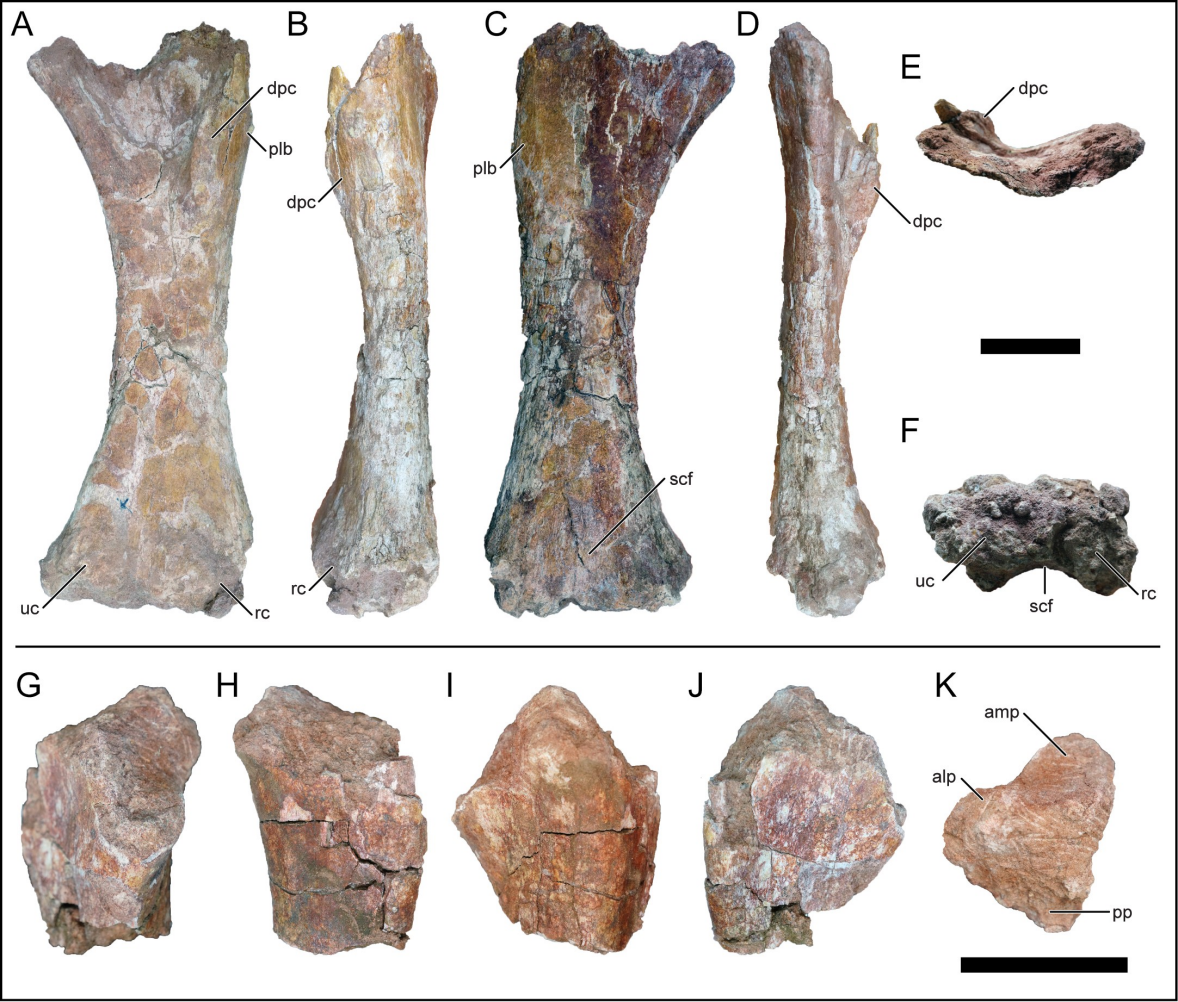
Forelimb elements of *Mnyamawamtuka moyowamkia*. A–F, left humerus, and G–K, partial left ulna. A, J, anterior; B, lateral; C, I, posterior; D, H, medial; E, proximal; F, distal; G, anteromedial; and K, cross section views. Anterior towards the top of the page in E, F, and K. Abbreviations: alp, anterolateral process; amp, anteromedial process; dpc, deltopectoral crest; plb, posterolateral bulge; pp, posterior process; rc, radial condyle; scf, supracondylar fossa; uc, ulnar condyle. Scale bars equals 10 cm.

Both a partial left and distal right humeri were recovered. The left humerus does not preserve the proximal portion (e.g., humeral head, proximal coracobrachialis fossa) or distal margins of both condyles (Fig 21), however a distal right humerus preserves the condyles. Based on what is preserved, the coracobrachialis fossa is wide and shallow, differing from the deep condition in *R*. *bisepultus* [11] but similar to *S*. *songwensis* [12] and *M*. *dixeyi* [3]. The attachment for the coracobrachialis muscle is a reduced tuberosity within this fossa. The gently rounded apex of the deltopectoral crest is near the level of the distal portion of the coracobrachialis fossa and is directed anteriorly with a minor medial deflection. The rounded apex of the deltopectoral crest differs from the slight laterally deflected apex of the deltopectoral crest in *R*. *bisepultus* [11]. The base of the deltopectoral crest is slightly expanded transversely though not to the extent exhibited in several titanosaurians such as *S*. *loricatus* and *N*. *australis* from the Late Cretaceous of South America [18, 29, 33]. A posterolateral bulge is present near the level of the deltopectoral crest (Fig 21A and 21C) and is similar to *R*. *bisepultus* [11], *M*. *dixeyi* (MAL-221, E.G., pers. obs., 2014, 2015), and other saltasaurid titanosaurians [29, 33]. The posterior surface of the humerus is gently convex at the midshaft. The medial margin is concave whereas the lateral surface is nearly straight along the long axis of the element (Fig 21A and 21C), a condition similar to *A*. *adamastor* [89]. The humeral midshaft is elliptical and differs from the subquadrangular cross-section of *R*. *bisepultus* [11]. The supracondylar fossa of the distal portion of the posterior surface is relatively shallow with a medial and lateral ridge bounding the fossa but are not as well developed as in *P*. *stromeri* [2]. Both radial and ulnar condyles are only slightly expanded anteriorly but not posteriorly (Fig 21B) and the radial condyle is not as well developed as in *P*. *stromeri* [2]. The radial condyle is not divided on the anterior surface (i.e., a notch is absent), a trait proposed to be a synapomorphy for the clade of Titanosauria and *C*. *insignis* [29], or lithostrotian titanosaurians [33]. Although eroded on the left humerus, the distal margin is leveled with poorly demarcated condyles, unlike in saltasaurid titanosaurians where the condyles are prominently developed and divided [18, 29].

### Ulna

A partial left ulna is represented by the proximal midshaft but does not preserve either proximal or distal articular ends (Fig 21G–21K). The cross section is tri-lobate with the anteromedial process projecting furthest from the center (Fig 21K), a condition exhibited in most titanosauriformes [18, 29]. The surface between the anteromedial and anterolateral processes is concave to accommodate the radius whereas the posteromedial (spanning the anteromedial and posterior processes) and posterolateral (spanning the anterolateral and posterior processes) surfaces are less concave and nearly flat.

### Metacarpals

**Fig 22:**
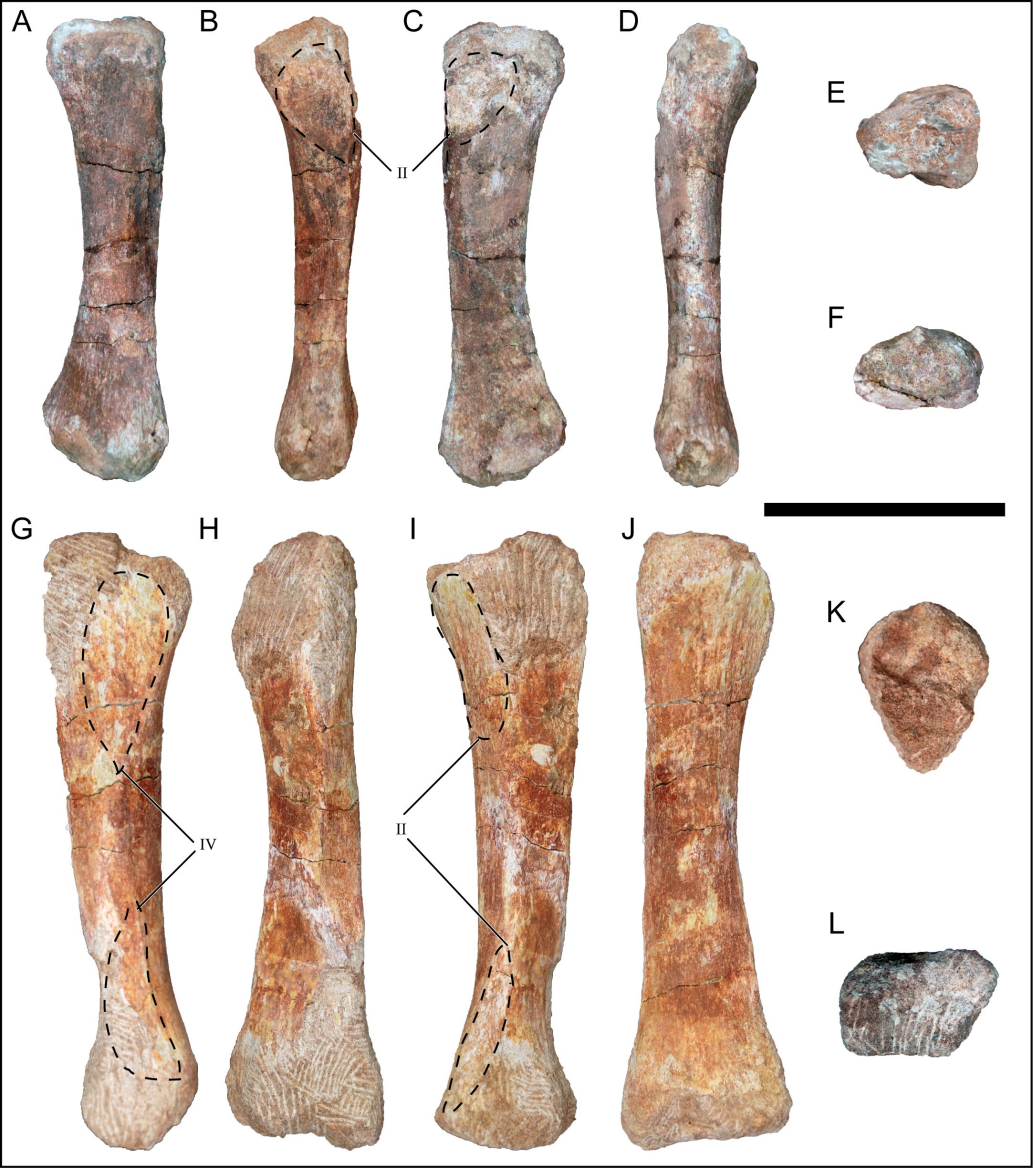
Metacarpals of *Mnyamawamtuka moyowamkia*. A–F, left metacarpal I, and G–L, left metacarpal III. A, G, lateral; B, H, anterior; C, I, medial; D, J, posterior, E, K, proximal; and F, L, distal views. Anterior towards top of page in E, F, K, and L. Abbreviations: II, articular facets for metacarpal II; IV, articular facets for metacarpal IV. Scale bar equals 10 cm.

A complete right metacarpal I (Fig 22A–22F) and nearly complete left metacarpal III (Fig 22G–22L) were recovered. As in most sauropods, metacarpal III is longer than metacarpal I whereas the opposite is true for the derived condition in the Late Cretaceous saltasaurid titanosaurians *A*. *sanjuanensis* [85], *E*. *sciuttoi* [73], and *O*. *skarzynskii* [63] (Table 2). In metacarpal I, the proximal portion of the element is slightly deflected posteriorly (Fig 22B). The proximal anterior surface exhibits an ovoid-to-subtriangular rugose area for articulation with metacarpal II (Fig 22B and 22C) in contrast to the strong proximoposterior orientation of this face in the titanosauriform *W*. *wattsi* from the Cenomanian of Australia [84]. The shaft is nearly straight with a slight medial bow. The distal end is beveled like other early titanosaurians such as *D*. *matildae* [65], *R*. *krausei* [55], and *E*. *sciuttoi* [73], but unlike few titanosaurians that exhibit a deflected distal third of metacarpal I (e.g., *A*. *delgadoi* [81]; *Argyrosaurus superbus* [92]). Considering *M*. *dixeyi* (MAL-208-2), Gomani [3] identified a metacarpal II but the morphology is more consistent with the element as metacarpal I (Gorscak pers. obvs., 2014; [84]). The posterior surface of the element is convex whereas the anterior surface is nearly flat to accommodate metacarpal II. The undivided distal condyles are restricted to the distal margin as exhibited in titanosauriform sauropods generally [18, 29, 93].

The proximal articular surface of metacarpal III, although incomplete, is triangular in cross section with a posterior apex and convex anterior margin (Fig 22K). The posterior surface is transversely compressed and continues as a ridge distally. The distal end is subrectangular in cross section with a transversely oriented long axis and a slight medial projection from the anteromedial corner (Fig 22L). The posteromedial surface gently bows medially whereas the posterolateral surface maintains a nearly straight profile until it expands at the distal end. There is an ovoid posteromedial articular surface along the proximal and distal surfaces for articulation with proximal and distal ends of metacarpal II (Fig 22J), and similar articular surfaces on the posterolateral surface for metacarpal IV (Fig 22H). The distal condyles are rounded along the anterior and distal margins, as exhibited in titanosauriformes [29]. The distal margin of metacarpal III is weakly deflected medially.

### Pubis

**Fig 23:**
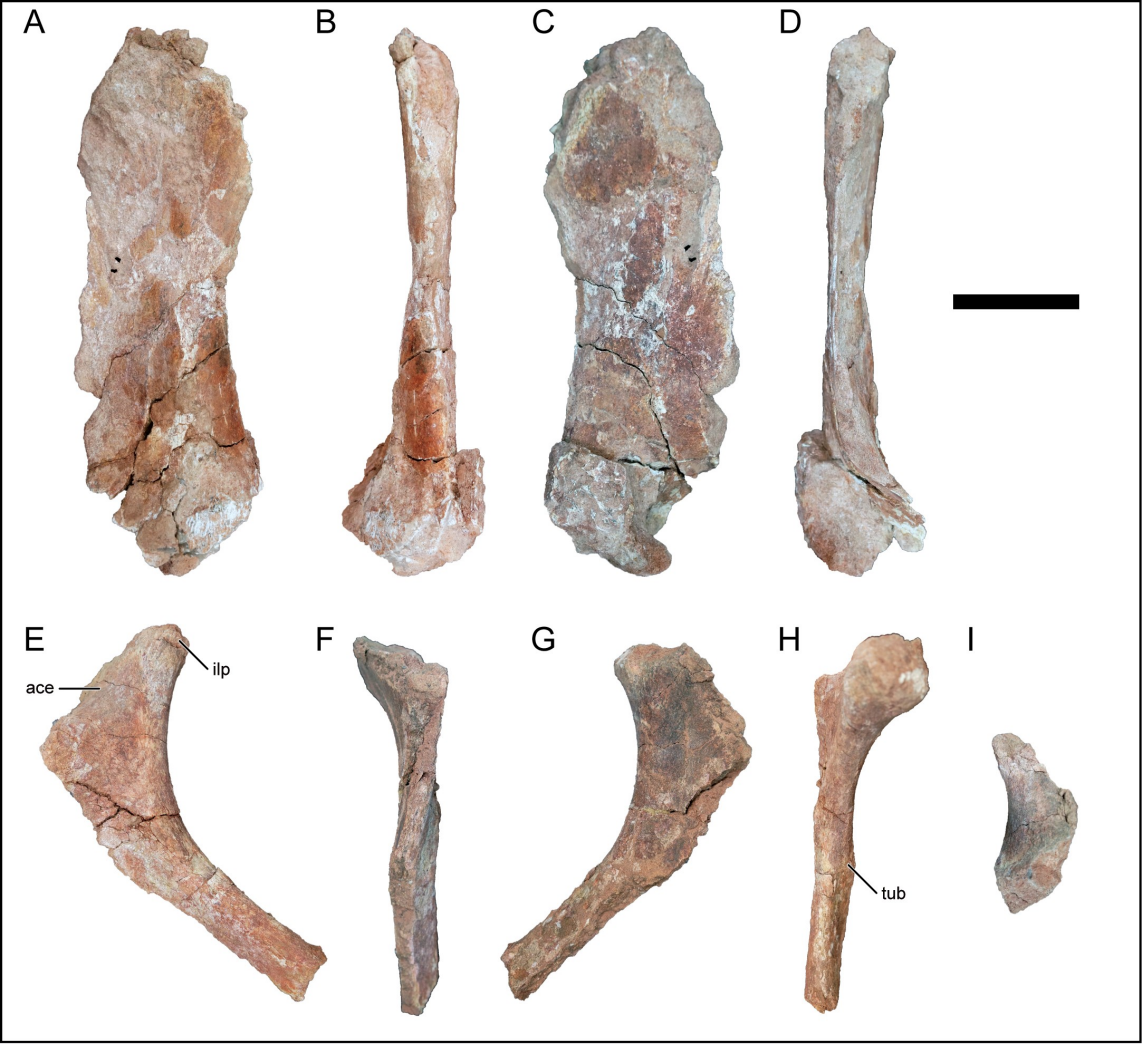
Pelvic elements of *Mnyamawamtuka moyowamkia*. A–D, right pubis, and E–I, right ischium. A, G, lateral/external; B, F, anterior; C, E, medial/internal; D, H, posterior; and I, proximal views. Anterior towards top in I. Abbreviations: ace, acetabulum contribution; ilp, iliac peduncle; tub, tubercle. Scale bar equals 10cm.

A partial right pubis and a left pubic midshaft were recovered and the description will focus on the more complete right pubis (Fig 23A–23D). The proximal portion of the element is not preserved and precludes statements on the ischial and ilia articular surfaces and morphology related to the size and position of the obturator foramen. The pubic blade is thin posteriorly and is thickened along the anterior margin. There is no longitudinal ridge on the lateral/external surface as in *F*. *dukei* [75, 76], and *A*. *delgadoi* [81]. The pubic blade is concave along the anterior and posterior margins. Judging from the partial right ischium, the pubis is likely the longer of the two elements but cannot be determined as currently preserved. A longer pubis relative to the ischium is a condition exhibited in all titanosaurians [48, 29].

### Ischium

The partial right ischium does not preserve much of the distal blade and proximal portions such as the pubic peduncle (Fig 23E–23I). The short iliac peduncle is rounded along the lateral margin but the medial portion is not preserved. The acetabular surface appears broadly concave. In most titanosaurians, the iliac peduncle is elongated and more anterodorsally directed thereby decreasing the angle of the ischial contribution to the acetabulum [29, 33]. On the proximolateral portion of the ischial blade surface, there is a small pointed tubercle for attachment of the flexor tibialis internus that is exhibited broadly in titanosauriform sauropods [29].

### Femora

**Fig 24:**
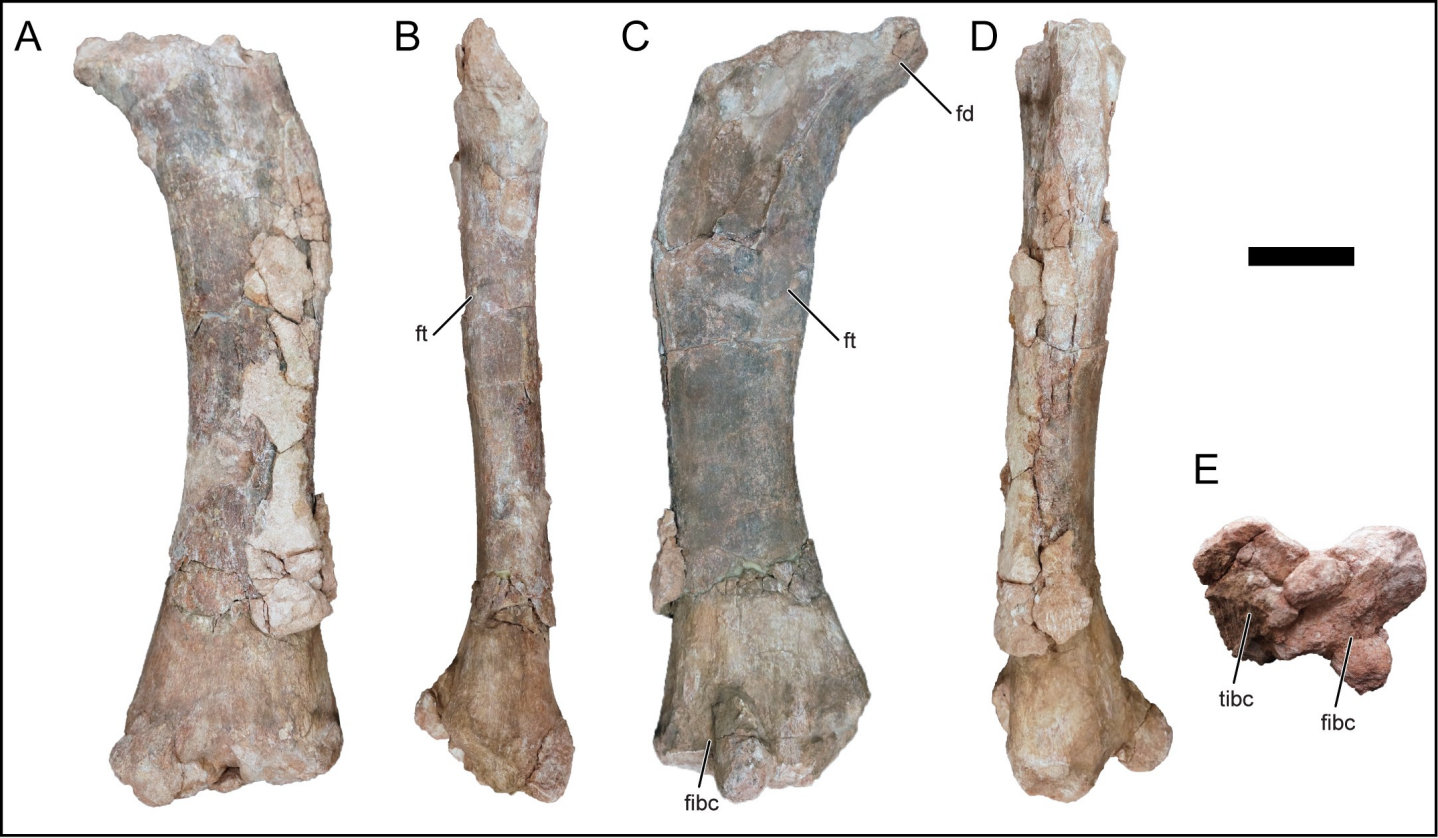
Left femur of *Mnyamawamtuka moyowamkia*. A, anterior; B, medial; C, posterior; D, lateral; E, distal views. Anterior towards top of page in E. Abbreviations: fibc, fibular condyle; ft, fourth trochanter; tibc, tibial condyle. Scale bar equals 10 cm.

A right femoral midshaft and a nearly complete left femur were recovered (Fig 24). Concerning the left femur, some of the proximal end is eroded whereas the anterolateral margin of the midshaft exposes delicately preserved cortical bone. The proximal femur exhibits the traditional titanosauriform trait of a proximolateral bulge [18, 29, 33, 48]. The weakly convex anterior surface exhibits a faint median groove along the proximal half and lacks the longitudinal ridge that is present in saltasaurian titanosaurians [29, 94]. The medial margin is concave and the proximal medial surface is flat with lightly rugose anterior and posterior ridges, similar to the condition in the Late Cretaceous Asian titanosaurian *O*. *skarzynskii* [63]. The lateral margin of the femur distal to the proximolateral bulge is nearly straight. Proximal to the posterior midshaft, the caudofemoralis longus muscle attachment site (fourth trochanter) is a small and weakly-developed rugose ridge (Fig 24B and 24C), as exhibited in most somphospondylian titanosauriforms [29]. The midshaft is elliptical in cross-section with a transverse-to-anteroposterior length ratio of 1.56 and is below the proposed saltasaurid ratio of 1.85 but well within the range for most titanosauriformes [18, 95]. The distal end is transversely expanded relative to the midshaft with an anterior groove between the distal condyles (Fig 24E). The fibular condyle is expanded posteriorly whereas the tibial condyle is poorly preserved (Fig 24E). The distal portions of the condyles are not well preserved.

### Tibia

**Fig 25:**
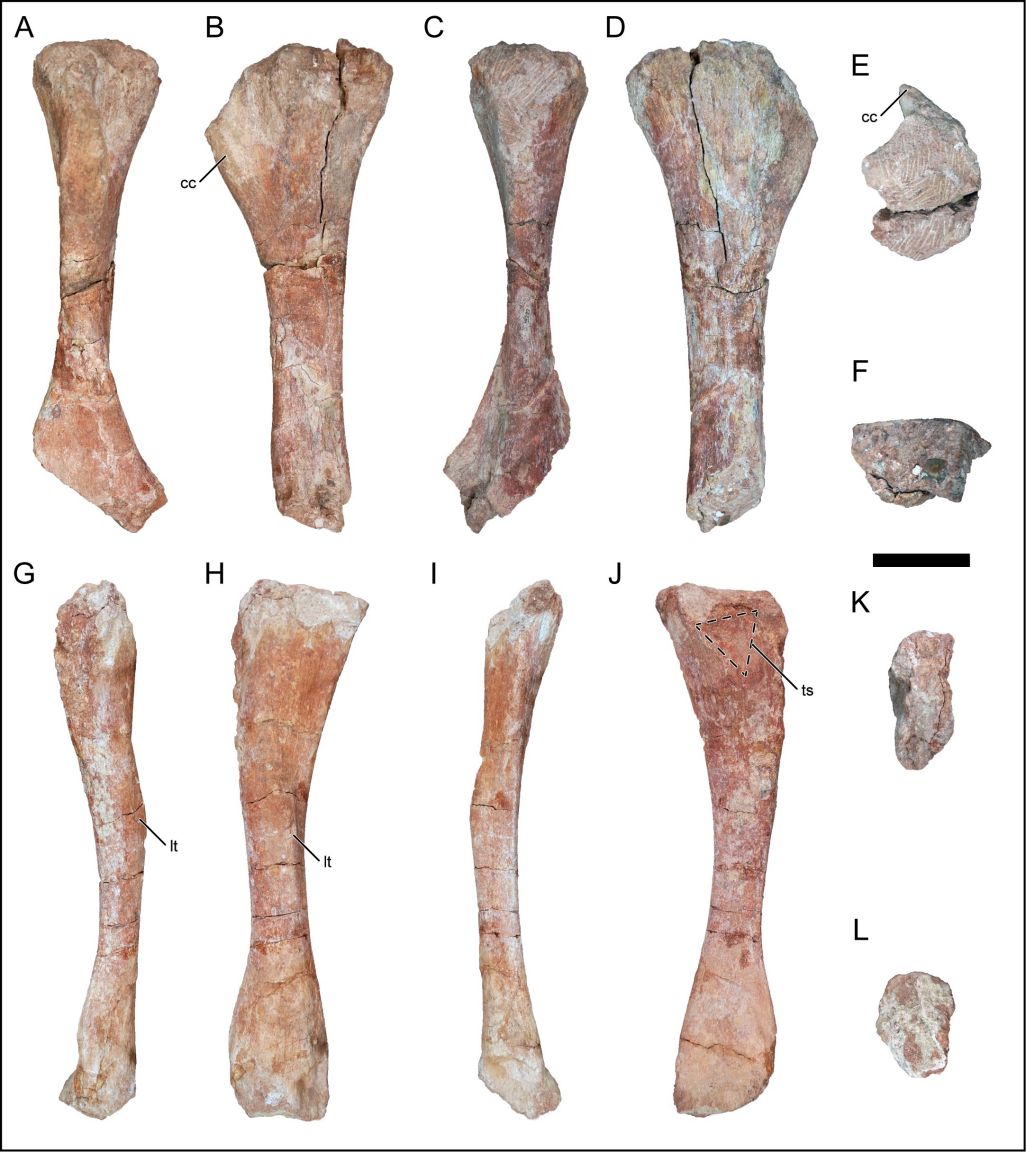
Lower hind limb elements of *Mnyamawamtuka moyowamkia*. A–F, left tibia and G–L, left fibula. A, G, anterior; B, H, lateral; C, I, posterior; D, J, medial; E, K, proximal; and F, L, distal views. Anterior towards top of page in E, F, K, and L. Abbreviations: cc, cnemial crest; lt, lateral trochanter; ts, triangular scar area. Scale bar equals 10 cm.

The left tibia is incomplete along the proximal and distal ends (Fig 25A–25F), and a partial midshaft of the right tibia was recovered. The proximal articular end is subequal in transverse and anteroposterior dimensions to the exclusion of the cnemial crest (Fig 25E) (Table 2). The cnemial crest is directed anteriorly with a slight lateral curve (Fig 25E), as is exhibited in other titanosauriforms like the euhelopodid *P*. *sirindhornae*, the titanosauriform *T*. *sanzi*, and the titanosaurians *R*. *krausei* and *M*. *dixeyi* [33]. The cnemial crest is concave along its lateral surface to accommodate the proximal fibula and there is no second cnemial crest (i.e., an additional articulating ridge) as in some somphospondylian titanosauriforms [31, 33]. The anterior margin of the cnemial crest is rugose along its medial edge. The tibia is anteroposteriorly elongated proximally and shortens towards midshaft. The midshaft cross section is anteroposteriorly elliptical. The distal end twists and significantly expands along the mediolateral axis. The mediolateral width of the preserved distal end is nearly twice the anteroposterior length of the midshaft (Table 2), a proposed trait for lithostrotian titanosaurians [33]. The anterior face of the distal end is nearly flat. From the proximal to distal end, the posterior surface is transversely narrow and ridge-like (Fig 25C).

### Fibula

A complete left fibula was recovered (Fig 25G–25L). The slender fibula is weakly sigmoidal unlike the strongly sigmoidal condition exhibited in most titanosaurians and several titanosauriformes such as *T*. *sanzi* [29, 33, 88]. The shaft of the fibula is straight and not twisted along the midshaft as in the Late Cretaceous titanosaurian *Uberabatitan ribeiroi* from Brazil [96]. The proximal end is anteroposteriorly elongate with the anteromedial process narrowed and medially deflected to articulate with the tibial cnemial crest (Fig 25K). The posterior corner of the proximal fibula forms a sharp angle, a trait that differs from the rounded condition seen in *R*. *krausei* [55]. The medial surface of the proximal end exhibits a weakly rugose triangular scar that is proximodistally longest along the anterior margin (Fig 25J). A triangular scarred area is absent in most titanosauriform sauropods [29] but is present and more pronounced within the Australian titanosaurian *D*. *matildae* [65]. The fibula bows laterally with the apex occurring near the lateral tuberosity (Fig 25G and 25H). The lateral tuberosity is a low rounded ridge with a second and subtler anterior ridge that is exhibited in most titanosaurians and some titanosauriforms [33]. The lateral trochanter as a prominent ridge is a derived state that is exhibited by some Late Cretaceous South American titanosaurians like *U*. *ribeiroi* [96], *N*. *australis* [97], and *D*. *schrani* [98]. The distal end is slightly expanded anteroposteriorly and exhibits a subrectangular cross section (Fig 25L). The medial surface of the distal end exhibits a shallow fossa just proximal to a medially expanded lip (Fig 25G). The distal end faces ventrolaterally which is shared with *T*. *sanzi* [88]. The anterior margin of the distal end is slightly crest-like. Overall, the fibula is similar to the fibula referred to *M*. *dixeyi* from the neighboring Dinosaur Beds of Malawi (MAL-189; [3]).

### Metatarsals

**Fig 26:**
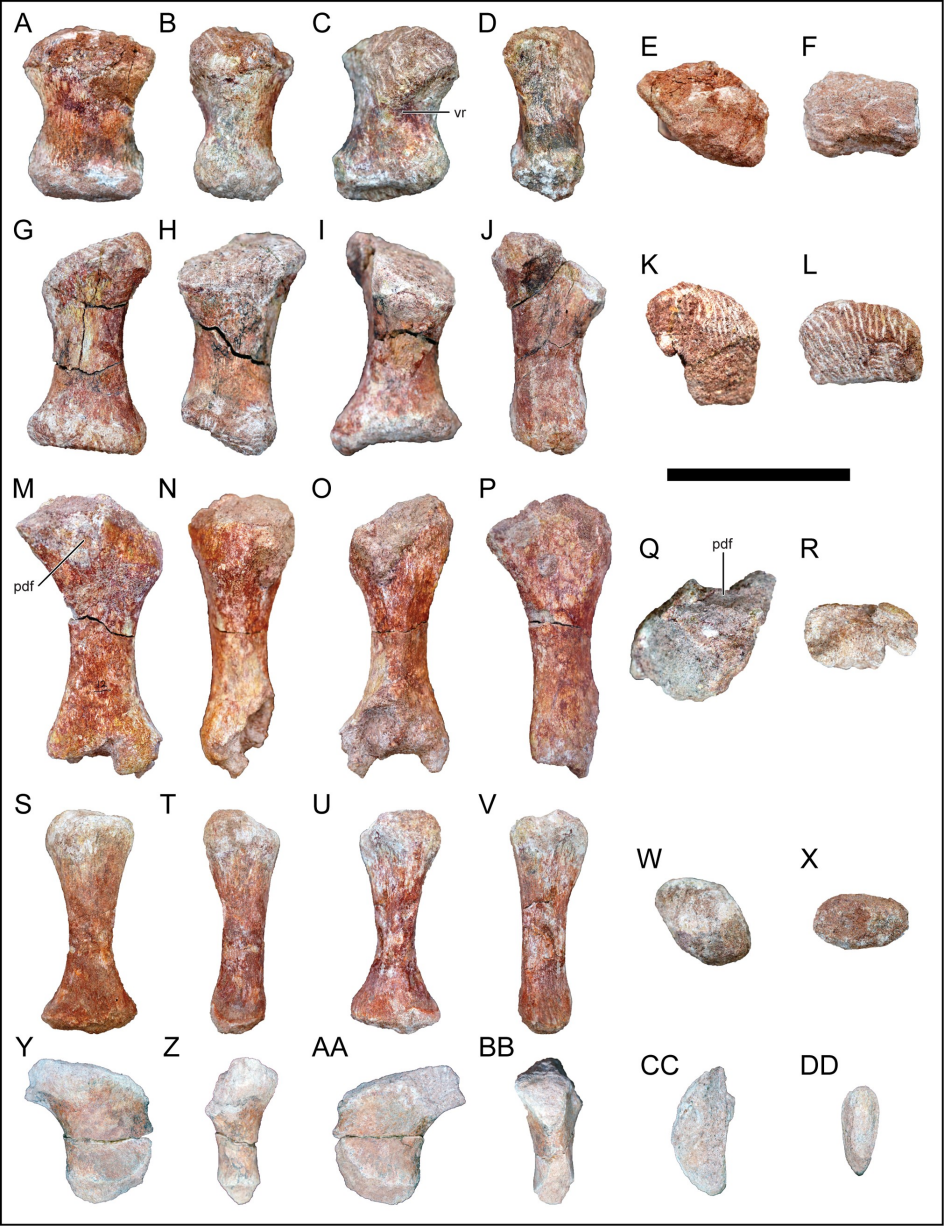
Metatarsal elements of *Mnyamawamtuka moyowamkia*. A–F, left metatarsal I; G–L, left metatarsal II; M–R, right metatarsal III; S–X, metatarsal IV; Y–DD. A, G, M, S, Y, dorsal; B, H, N, T, Z, lateral; C, I, O, U, AA, plantar; D, J, P, V, BB, medial; E, K, Q, W, CC, proximal; and F, L, R, X, DD, distal views. Anterior towards top of the page in E, F, K, L, Q, R, W, X, CC, and DD. Abbreviations: pdf, proximodorsal fossa; vr, ventral ridge. Scale bar equals 10 cm.

Left metatarsal I is a stout element with transversely expanded proximal and distal ends (Fig 26A–26F). Metatarsal I is the shortest element of the metatarsus (Table 2). The proximal end is not significantly more expanded transversely than the distal end as in *Ligabuesaurus leanzai* from the Aptian of Argentina [99], *R*. *krausei* from the Late Cretaceous of Madagascar [55], and *T*. *sanzi* [88]. The element is slightly twisted around the long axis. The medial margin is shorter than the lateral margin, as the distal surface is beveled slightly medially (Fig 26C). The element is mildly concave along the medial and lateral margins. The proximoventral surface exhibits a short ventral ridge for articulation with metatarsal II (Fig 26C). The distal condyle is subrectangular in cross section and the medial condyle is slightly more expanded than the lateral condyle; however, these two condyles are barely distinguishable and not well-developed (Fig 26F). The element compares favorably with *Notocolossus gonzalezparejasi* from the Late Cretaceous of Argentina [100], *E*. *sciuttoi* [73], and the *T*. *sanzi* [88].

Left metatarsal II is mostly complete and only missing portions of the proximal end (Fig 26G–26L). Similar to metatarsal I, the element is stout with a medially beveled distal end (Fig 26H). The element exhibits a similar degree of twisting along the long axis as seen in metatarsal I. Both proximal and distal ends are transversely expanded (Fig 26G). The outline of the proximal end is subrectangular with a strongly convex dorsal margin for articulation with the lateral margin of metatarsal I (Fig 26K). The ventral surface is strongly ridge-like for articulation with metatarsal I medially and metatarsal III laterally. The distal end is similar to metatarsal I, exhibiting a subrectangular outline with a slightly more expanded medial margin and barely discernible condyles (Fig 26L). Metatarsal II is similar to metatarsal II of *R*. *krausei* and *N*. *gonzalezparejasi* but not as transversely expanded in the latter.

Right metatarsal III is relatively more slender at midshaft when compared to both metatarsal I and II but with transversely expanded proximal and distal ends (Fig 26M–26R). The dorsomedial margin is markedly expanded with a proximodorsal fossa (Fig 26M). The twist along the long axis is not as strong as in metatarsal I and II and exhibits subequal lengths along the medial and lateral margins. There is a narrow ridge along the ventral surface for articulation with metatarsal II medially and IV laterally. The distal end is incomplete, but would have appeared to have a better developed condyles relative to the other metatarsals. The element compares favorably with metatarsal III of *R*. *krausei* [55].

Left metatarsal IV is the most slender metatarsal with only minimal twisting along the long axis (Fig 26S–26X). The length of metatarsal IV is subequal to that of metatarsal III. The distal end is more expanded relative to the proximal end, a condition exhibited in *Bonitasaura salgadoi* from the Santonian of Argentina [101]. In *R*. *krausei*, metatarsal IV exhibits a more expanded proximal end than distal end [55], and in *N*. *gonzalezparejasi*, both ends are subequal in length [100]. Both proximal and distal ends are subovoid in outline (Fig 26W and 26X). The ventral surface bears a less-pronounced ridge than the other metatarsals. The distal condyles are not discernible.

Left metatarsal V is nearly complete with some erosional surfaces along the proximal and distal ends (Fig 26Y–26DD). The proximal end is expanded dorsally as the remainder of the element is nearly the same width until the blunt and rounded distal end. The element is mostly flat and is mildly concave along the medial margin where it abuts metatarsal IV. Furthermore, the element is gently convex along the lateral margin. Overall, both metatarsal I and V are roughly subequal in length.

### Pedal phalanx

**Fig 27:**
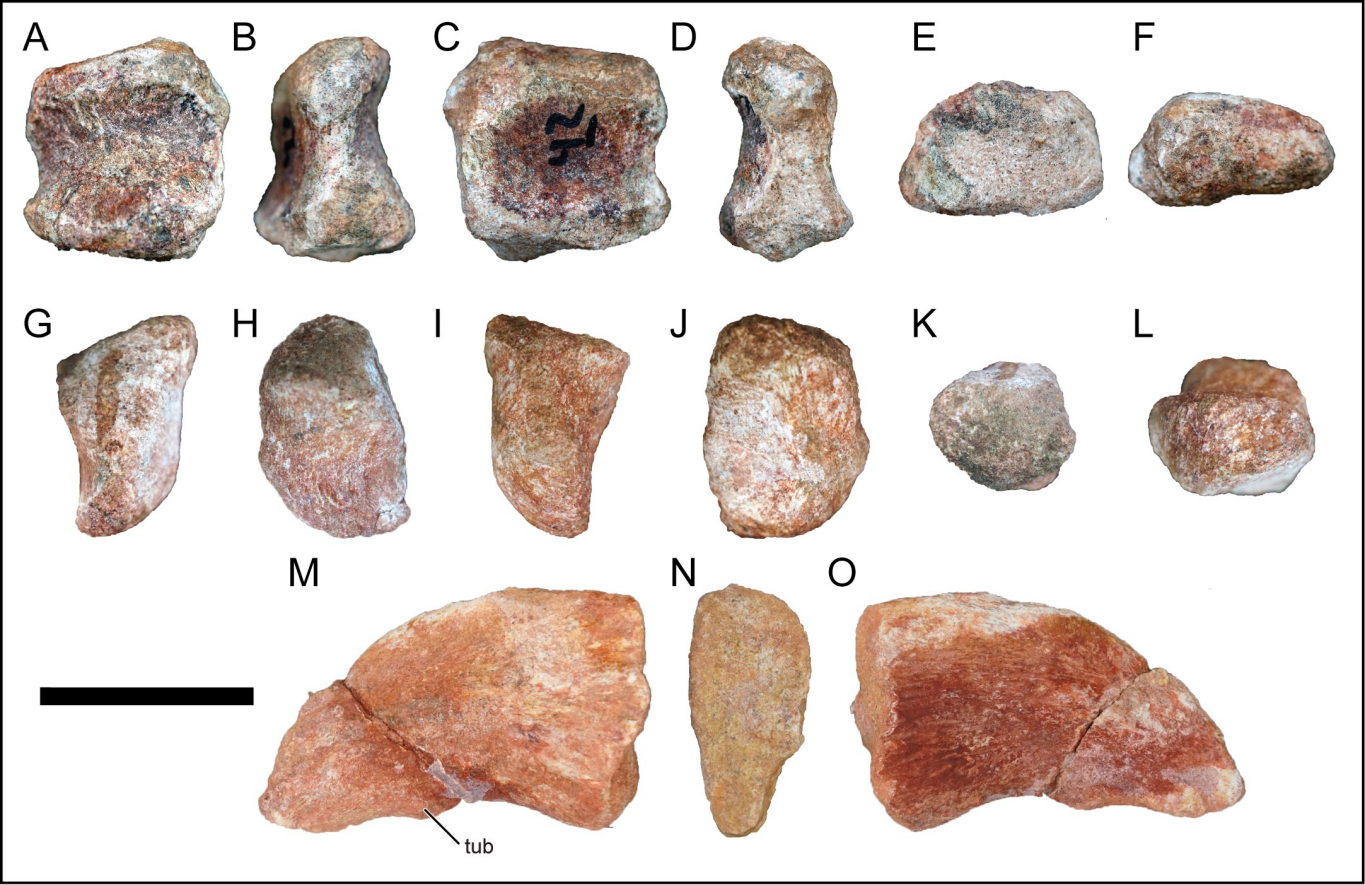
Pedal elements of *Mnyamawamtuka moyowamkia*. A–F, left pedal phalanx, and G–L, and M–O, left pedal unguals. A, G, M, dorsal; B, H, medial; C, I, plantar; D, J, O, lateral; E, K, proximal; F, L, distal views. Anterior t owards top of page in E, F, K, and L. Abbreviations: tub, tubercle. Scale bar equals 5 cm.

A left phalanx was recovered (Fig 27A–27F). The position within the pes is unknown due to the lack of other phalanges. The proximal surface is D-shaped (Fig 27E), with a flat ventral margin and convex dorsal margin. The medial margin is longer than the lateral margin as the distal end is beveled laterally (Fig 27A). The distal articular surface is smooth for articular with another ungual and exhibits a similar D-shape outline as the proximal surface (Fig 27F).

### Unguals

Two unguals were recovered (Fig 27G–27O). Left ungual I is sickle-shaped (Fig 27M–27O). The ungual is nearly twice as long as it is wide. The lateral margin is gently concave and the medial margin is strongly convex. Along the medial curvature there is a small tuberosity near mid-length (Fig 26M), as exhibited in the titanosauriform *T*. *sanzi* [88], several euhelopodids [33] and in to the titanosaurian *M*. *dixeyi* (MAL-211; Gorscak pers. obvs., 2014). Weakly developed grooves follow the curvature of the element along the proximoventral surface, similar to that described in unguals of the Naashoibito specimen [102]. The other ungual pertains either to the third or fourth digit (Fig 26G–26L). This ungual is significantly smaller and is stouter. It is only weakly sickle shaped with a wide proximal articular surface.

## Phylogenetics

The latest iteration of the Gorscak and O’Connor [17] dataset was used to assess the phylogenetic affinities of *M*. *moyowamkia* under both parsimony and Bayesian analytical regimes. The data set includes the recently erected African titanosaurians *S*. *songwensis* from the Namba Member of the Galula Formation [12], *M*. *shahinae* from the Campanian Dakhla Oasis of Egypt [16], *N*. *gonzalezparejasi* [100], *Patagotitan mayorum* [103], and revised scorings for several taxa in addition to several new characters (S1 File). A total of 55 taxa and 532 morphological characters, composed of variable and autapomorphic characters (272 and 260 respectively) are included in the data set. The autapomorphic characters are utilized in the Bayesian analyses to inform the rate of morphological change along terminal branches, crucial information in tip-dating, and contribute to the calculation of model likelihood scores via the morphological models of evolution [11, 104–106]. Furthermore, revised stratigraphic ranges/tip dates for *M*. *moyowamkia*, *R*. *bisepultus*, and *S*. *songwensis* were utilized based on recent paleomagnetic stratigraphy data that has placed new age constraints on both the Mtuka and Namba members of the Galula Formation [50]. Here, the Mtuka Member is constrained to Aptian–Cenomanian and the Namba Member is constrained to the preferred hypothesis of late Campanian (Chron c32) according to Widlansky et al. [50]. All characters are assumed to be independent and unordered. All nexus files and tree files are available as supplemental information (S2–S8 Files). For the parsimony analysis, the autapomorphic characters were omitted as they are uninformative in this analytical paradigm. The parsimony analysis was conducted in TNT v1.1 [107] using random taxon addition and tree-bisection-reconnection options under the heuristic search. The results of the parsimony analysis yielded 684 most parsimonious trees with a tree score of 900 steps. Additionally, the uncalibrated Bayesian analyses followed the protocol in Gorscak et al. [11, 12] and Gorscak and O’Connor [17]. The Mk likelihood model was assumed for the evolution of morphological characters [104]. Two models were tested: (1) equal rates of character state change model; and (2) variable rates of character state change model (sampled from a gamma-distribution). The Markov chain Monte Carlo (MCMC) ran for 20 million generations with one hot and one cold chain. The chains sampled tree-space every one thousand generations and the first 25% of the posterior distribution was discarded to eliminate the initial climbing phase. The models were compared using the Bayes Factor, which is calculated by twice the difference of the harmonic mean log likelihood of each model [108]. The harmonic mean log likelihood of the equal-rates model was -4688.140, whereas the harmonic mean log likelihood of the variable-rates model was -4527.737, resulting in a Bayes Factor of 320.806 and suggesting the variable-rates model is strongly preferred over the equal-rates model. Finally, tip-dating Bayesian phylogenetic analyses were conducted to jointly estimate phylogenetic relationships with estimated divergence dates and branch lengths based on the additional data of stratigraphic information [16, 17, 109, 110]. The R package BEASTmasteR [111] produced the XML files for BEAST 2.2.4 [112]. The assumed tree model for this set of analyses was the birth-death-skyline-serial-sampling as it allows both the birth (origination) and death (extinction) rates to vary across time [113]. A relaxed clock was assumed under a lognormal distribution of sampled rates. Both rates of character change were tested under equal and variable (with an assumed gamma-distribution) assumptions, similar to the uncalibrated Bayesian analysis set. The MCMC persisted for 20 million generations with sampling of tree-space occurring every 1,000 generations and the first 25% the sample was discarded. Each stratigraphic range of each taxon was sampled under a uniform distribution to account for stratigraphic uncertainty (see S1 File). The model with the variable rates of character evolution (log-likelihood of -4392.279) was strongly preferred over the model that assumed equal rates of character change (log-likelihood of -4466.479) for a resulting Bayes Factor of 148.400.

**Fig 28:**
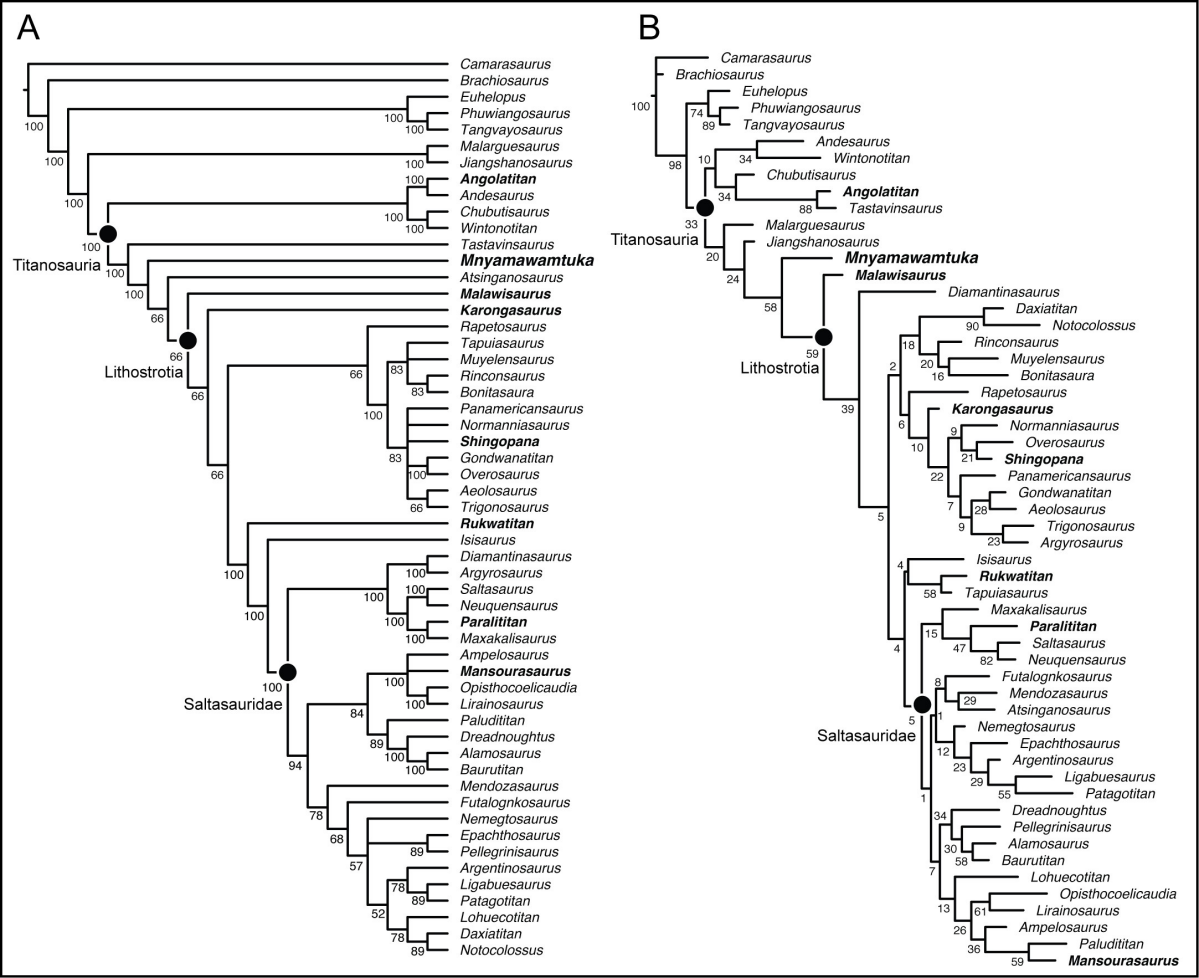
Results of parsimony and uncalibrated Bayesian phylogenetic analyses. Results of the majority-rule consensus tree of the parsimony phylogenetic analysis, A, and maximum clade credibility tree of the variable-rates Bayesian phylogenetic analysis, B. Numbers at the node represent frequency of recovered clades in A, and posterior probabilities in B.

The majority rule consensus tree from the parsimony analysis (Fig 28A) broadly resembles the previous parsimony iterations of this matrix (e.g., [12, 16]). Concerning the Galula Formation titanosaurians from Tanzania, *M*. *moyowamkia* was recovered as non-lithostrotian titanosaurian, *R*. *bisepultus* as the sister taxon to the lithostrotian clade that includes the *I*. *colberti*, *N*. *mongoliensis*, and Saltasauridae, and *S*. *songwensis* within Rinconsauria with this group as the sister clade to aeolosaurid titanosaurians from South America (e.g., *G*. *faustoi*, *T*. *pricei*), and the Albian *Normanniasaurus genceyi* from France. The titanosaurians *M*. *dixeyi* and *K*. *gittelmani* from the Aptian Dinosaur Beds of Malawi were recovered as successive sister taxa to the unity of the saltasaur- (those titanosaurians more closely related to *S*. *loricatus* than *A*. *maximus*) and aeolosaur-lineages (those titanosaurians more closely related to *A*. *maximus* than *S*. *loricatus*). The Cenomanian *P*. *stromeri* from Egypt was recovered within the saltasauridae with a sister relationship to *M*. *topai* from the Upper Cretaceous of South America. *A*. *adamastor* was recovered as the sister taxon to *A*. *delgadoi* within the larger Andesauroidea clade (e.g., *C*. *insignis*, *W*. *wattsi*). The topological placement of *M*. *moyowamkia* within Titanosauria is supported by several characters: the presence of cylindrical and slender teeth (excluding *M*. *dixeyi*, *A*. *atacis*, and *L*. *leanzai*); simple and/or undivided pleurocoels of the cervical vertebrae (excluding *S*. *loricatus*); narrowing of the neural canal in the cervical vertebrae; and posterolateral bulge near the deltopectoral crest on the humerus (excluding *A*. *superbus* and *D*. *matildae*). Overall, the results of the parsimony analysis suggest that Africa was an important area for the early evolution of titanosaurians and more specifically, early lithostrotian evolutionary history (which is somewhat self-evident as Lithostrotia is partly defined by *M*. *dixeyi*; [19]). This is further supported with a couple of European titanosaurians (*T*. *sanzi* and *Atsinganosaurus velauciensis*) interspaced with the African titanosaurians along the lithostrotian stem that is consistent with an Early Cretaceous Eurogondwanan model [114], with a similar pattern later during the Late Cretaceous with *Mansourasaurus* nested with Eurasian titanosaurians. *K*. *gittelmani* (Malawi), *M*. *dixeyi* (Malawi), and *M*. *moyowamkia* (Tanzania), are outgroups to the node uniting the aeolosaur-lineage and the saltasaur-lineage whereas *P*. *stromeri*, *S*. *songwensis* and *R*. *bisepultus* are recovered within Lithostrotia. Although naive of temporal information, the parsimony results is consistent with the new paleomagnetic dates of the Galula Formation, with *P*. *stromeri*, *S*. *songwensis*, and *R*. *bisepultus* more closely related to Late Cretaceous lithostrotian titanosaurians than with other Early Cretaceous members (e.g., *M*. *dixeyi*, *T*. *sanzi*).

Concerning the variable-rates model for the uncalibrated Bayesian phylogenetic results Fig 28B, it should be noted that posterior probabilities in all the Bayesian analyses vary widely and are likely a result of the degree of missing data, lack of overlapping characters amongst the taxa, and the effects of the different model parameters summarize the lack of precision but not necessarily its accuracy (e.g., [115]). Concerning African titanosaurians, their general placement is not considerably different from the results of the parsimony analysis: *M*. *dixeyi* and *M*. *moyowamkia* were recovered along the Lithostrotia lineage; *R*. *bisepultus* was recovered in a subclade with *T*. *macedoi* and *I*. *colberti* as the sister clade to Saltasauridae; *P*. *stromeri* was recovered in a subclade (*M*. *topai*, *S*. *loricatus*, *N*. *australis*) that is sister to the union of Lognkosaurians, Afro-Eurasian titanosaurians, and those that bear a biconvex first caudal vertebra (e.g., *A*. *sanjuanensis*, *B*. *britoi*); *S*. *songwensis* is recovered as the sister taxon to *O*. *paradasorum* from the Late Cretaceous of Argentina within Late Cretaceous aeolosaurids of South America; *K*. *gittelmani* was recovered as the sister taxon to the aeolosaurids with *R*. *krausei* as the next outgroup-taxon; *A*. *adamastor* is recovered within Andesauroidea and sister taxon to *T*. *sanzi;* and *M*. *shahinae* clustering with Eurasian titanosaurians with close relationship to *A*. *atacis* and *P*. *nalatzensis* [16]. Similar character support for *M*. *moyowamkia* in the parsimony analysis is applicable here including the presence of a median infrapostzygapophyseal lamina in the anterior dorsal vertebrae (although not present in several nested titanosaurians such as *A*. *huinculensis*, *L*. *leanzai*, and *D*. *schrani*).

**Fig 29:**
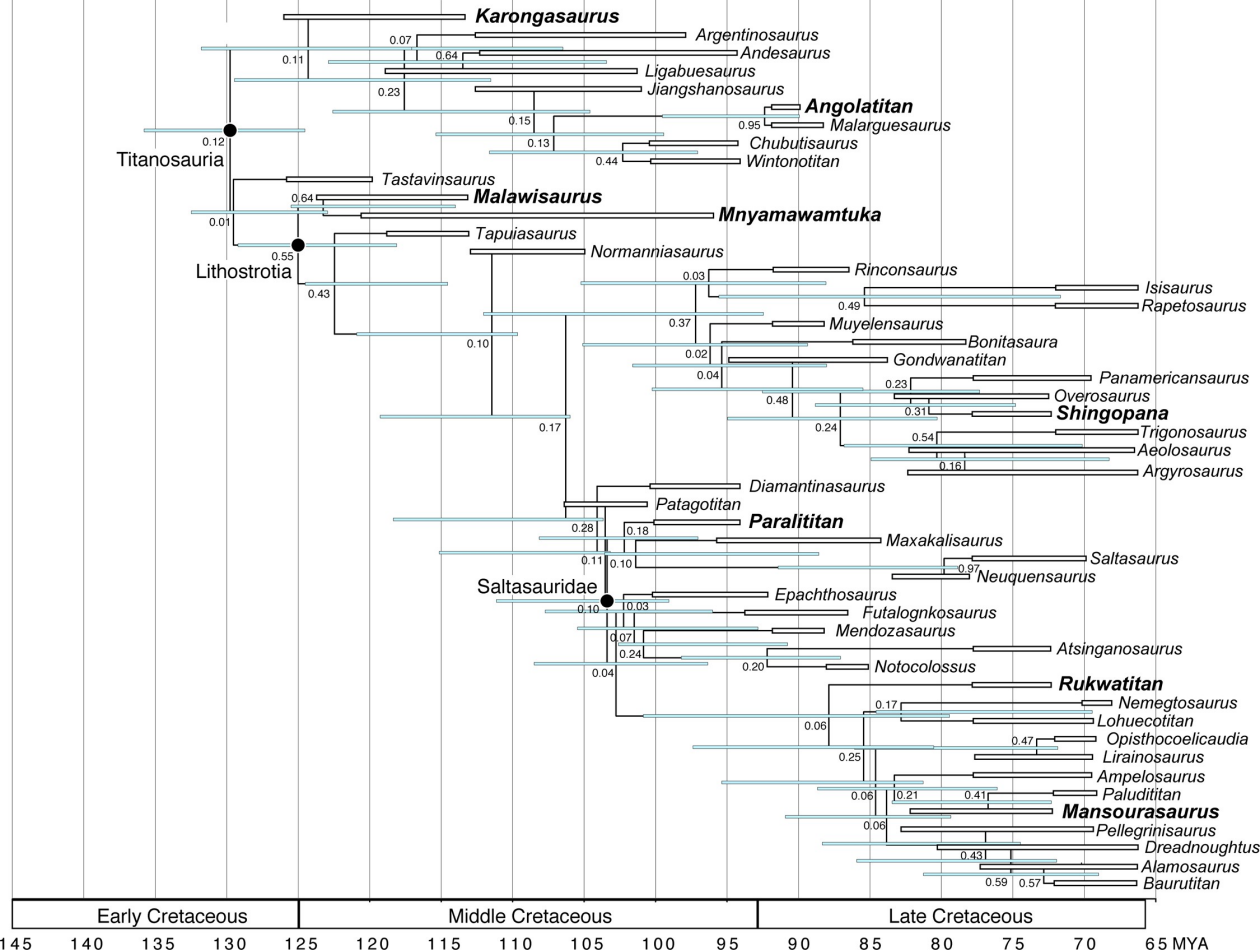
Results of tip-dating phylogenetic analysis. Maximum clade credibility tree for the variable-rates tip-dating Bayesian analysis. Numbers at the nodes represent posterior probabilities, blue bars at each node represent the 95% highest posterior density of each node age, and white bars at the tips represent the 95% highest posterior density of the sampled stratigraphic range of each terminal taxon. Abbreviations: MYA, millions of years ago.

Finally, tip-dating analyses that incorporate temporal/stratigraphic data produced a generally similar topology as the parsimony and uncalibrated Bayesian analyses and yielded consistent placement for the African titanosaurians (Fig 29). *M*. *moyowamkia* was recovered as the sister taxon to *M*. *dixeyi* (posterior probability of 0.64), and is slightly different from the successive outgroup relationships in the parsimony and uncalibrated Bayesian analyses. This result provides the first evidence of a sister relationship between members of the Galula Formation and Dinosaur Beds of Malawi under the current dataset (see [43]). Several characters support this placement of *M*. *moyowamkia* within Titanosauria: cervical parapophysis at least half the functional length of the centrum (excluding *M*. *pecheni*, *O*. *paradasorum*, *P*. *mayorum*, and *M*. *shahinae*); narrowing of the cervical neural canal; median infrapostzygapophyseal lamina; absence of hypantrum/hyposphene complex in the dorsal vertebrae (excluding *E*. *sciuttoi* and *P*. *mayorum*); and the presence of a posterolateral bugle near the deltopectoral crest of the humerus (excluding *A*. *superbus* and *D*. *matildae*). The members of the younger Namba Member clustered with other Late Cretaceous titanosaurians: *S*. *songwensis* nested within aeolosaurid titanosaurians from the Late Cretaceous of South America and *R*. *bisepultus* as the sister taxon to the clade of Late Cretaceous titanosaurians from Africa (e.g., *M*. *shahinae*), Asia (e.g., *O*. *skarzynskii*), Europe (e.g., *A*. *atacis*), North America (e.g., *A*. *sanjuanensis*), and South America (e.g., *B*. *britoi*). Elsewhere, *K*. *gittelmani* was recovered within Andesauroidea as the sister to all other members of this clade (e.g., *A*. *delgadoi*, *Jiangshanosaurus lixianensis*, *W*. *wattsi*) including *A*. *adamastor*, and, finally, *P*. *stromeri* was recovered in a subclade consisting of *M*. *topai*, *N*. *australis*, and *S*. *loricatus* as this clade in turn acting as the sister clade to the rest of Saltasauridae (clades of Lognkosauria, Afro-Eurasian titanosaurians, and titanosaurians that exhibit a biconvex first caudal vertebra). Overall, across each set of phylogenetic analyses, the African titanosaurians in the dataset were not recovered as monophyletic which is evidence that counters the idea of isolated African faunas for most of the Cretaceous but rather a complex and reticulated relationships with faunas from surrounding landmasses throughout most of the Cretaceous. Our results suggest a more prominent role of Africa than previously recognized during the early evolution of titanosaurians in the Early–mid Cretaceous, with African members in the Andesauroidea (e.g., *A*. *adamastor*), along the Lithostrotia lineage (e.g., *M*. *moyowamkia*, *M*. *dixeyi*), and nested within Lithostrotia (e.g., *P*. *stomeri*).

## Discussion and conclusions

*M*. *moyowamkia* provides a glimpse into the Mtuka Member (Galula Formation) fauna and significantly expands titanosaurian fossil record of Africa. Currently, the Galula Formation represents the most diverse titanosaurian assemblage for all of Africa in terms of named taxa (*R*. *bisepultus*, *S*. *songwensis*, and now *M*. *moyowamkia*). *Malawisaurus dixeyi* and *M*. *moyowamkia* represent two of the best known titanosaurians from Africa based on skeletal completeness when compared to other African titanosaurians. Outside of Africa, other putative early titanosaurians or closely related non-titanosaurian titanosauriformes are not as well represented anatomically and their descriptions are usually based on a certain skeletal region or exhibit a less complete skeleton compared to *M*. *moyowamkia*.

Concerning the Mtuka Member, *M*. *moyowamkia* represents the first formally described tetrapod from this unit. The Mtuka Member is comparatively less known than the Namba Member with a fauna represented by remains of lungfish, turtles, crocodyliforms, theropod dinosaurs, and additional sauropod remains [44]. Although the two members are superficially similar in faunal composition at higher taxonomic rankings, *M*. *moyowamkia* is decisively distinct from both *R*. *bisepultus* and *S*. *songwensis* based on anatomical comparisons of currently known common elements. The cervical vertebrae of *M*. *moyowamkia* lack the autapomorphies described for *R*. *bisepultus* (e.g., carotid processes, accessory tubercles near the capituloparapophyseal suture, deep fossa along the ventral surface of the diapophysis; [11]). The caudal vertebrae of *M*. *moyowamkia* and *R*. *bisepultus* differ in several regards: (1) the anterior caudal vertebrae in *R*. *bisepultus* are strongly procoelous whereas the anterior caudal vertebra are weakly procoelous in *M*. *moyowamkia*; (2) *R*. *bisepultus* exhibits a tubercle near the prezygapophysis on the spinoprezygapophyseal lamina; (3) the neural spine in *M*. *moyowamkia* is comparatively narrower and directed dorsally rather than posterodorsally in *R*. *bisepultus*; (4) *R*. *bisepultus* lacks the dorsolateral expansion of the posterior face of the middle caudal centrum that is unique in *M*. *moyowamkia*; (5) the postzygapophysis in *M*. *moyowamkia* is oriented obliquely and offset from the neural spine; and (6) the distal caudal vertebra is transversely narrower in *M*. *moyowamkia*. Additionally, none of the numerous recovered dorsal ribs of *M*. *moyowamkia* and *R*. *bisepultus* exhibit the anterior and posterior flanges that are characteristic of *S*. *songwensis*. The *M*. *moyowamkia* humerus is more anteroposteriorly expanded than seen in *S*. *songwensis* and lacks the autapomorphies seen in *R*. *bisepultus* [12]. Otherwise the *M*. *moyowamkia* and *S*. *songwensis* humeri are incomplete in such a way to prevent more confident comparisons (e.g., the *S*. *songwensis* humerus does not preserve the deltopectoral crest but this feature is preserved in *M*. *moyowamkia*). Finally, since *M*. *moyowamkia* is recovered in a phylogenetically distant position from both *S*. *songwensis* and *R*. *bisepultus*, which are recovered in positions more closely related to titanosaurians of the Late Cretaceous, the phylogenetic placement is consistent with the Mtuka Member as a distinct and older unit from the Namba Member [44, 50].

*M*. *moyowamkia* represents one of the most complete early titanosaurian sauropod skeletons known with elements representing dental and all major regions of the postcranial skeleton. Additionally, other South American titanosaurians (e.g., *A*. *delgadoi* and *L*. *leanzai*) from the middle Cretaceous (Aptian–Cenomanian) are comparatively less known in skeletal representation. *T*. *macedoi* preserves a nearly complete skull and associated post-cranial skeleton but this taxon has only been partially described as preparation and further studies on the specimen continue [35, 36]. With a fairly well-represented skeleton *M*. *moyowamkia* exhibits several previously proposed non-lithostrotian titanosauriform traits (e.g., plesiomorphic traits with respect to Lithostrotia): weakly procoelous anterior caudal vertebra and non-procoelous middle and distal caudal vertebrae [18, 19, 29, 33, 48]; caudal vertebrae lacking a ventral hollow [18, 29]; caudal vertebrae lack ventrolateral ridge [33]; a small sternal plate relative to the length of the humerus [29, 33]; and a seemingly broad ischial margin of the acetabulum [29]. On the other hand, *M*. *moyowamkia* also exhibits several previously proposed derived traits within Lithostrotia and/or more exclusive clades: neural canal narrows in the cervical vertebrae [29]; middle and posterior cervical vertebrae with elongate parapophysis [29, 33]; middle and posterior dorsal vertebrae lack hyposphene-hypantrum articulations [18, 29, 48]; and a bulge on the posterolateral aspect of the humerus [29]. The presence of such traits in *M*. *moyowamkia* suggests that some of these features were likely more widespread than previously recognized. For example, the narrowing of the neural canal in the cervical vertebrae was considered an autapomorphy in *R*. *krausei* [56], a potential synapomorphy for saltasaurids [29], and recently observed in *B*. *salgadoi* [101] and *S*. *songwensis* [12]. Since *M*. *moyowamkia* also exhibits this condition, the narrowing of the cervical neural canal may be indicative of a synapomorphy for most of Titanosauria and may be overlooked given variability in both style of preservation (e.g., the trait is only visible when the neural canal is exposed) and what is actually in the preserved anatomy (e.g., complete cervical series are rare within Titanosauria).

*R*. *bisepultus* and *S*. *songwensis* have been recovered as distantly related to, and morphologically distinct from, *M*. *dixeyi* [11, 12]. Now *M*. *moyowamkia* provides another critical opportunity to compare the titanosaurians recovered from the Galula Formation with those from the Dinosaur Beds of Malawi. Concerning *K*. *gittelmani*, *M*. *moyowamkia* does not preserve a dentary but the tooth morphs are potentially similar. Both the tooth Morph C from the *M*. *moyowamkia* quarry and the teeth described by Gomani [3] in the *K*. *gittelmani* dentary and referred ones are similar in that they are peg-like and have high angled wear facets. However, the teeth in the *K*. *gittelmani* dentary are unworn, minimally erupted, and at least apically, appear to be peg-like and not broad or spatulate (personal observation; E.G., 2014). More significantly, components of the skeleton of *M*. *moyowamkia* differ from those referred to *M*. *dixeyi* in numerous ways: (1) anterior cervical vertebrae in *M*. *moyowamkia* exhibit a small knob-like epipophysis dorsal to the postzygapophysis that is absent in *M*. *dixeyi*; (2) anterior cervical vertebrae in *M*. *moyowamkia* exhibit a dorsally pronounced neural spine whereas in *M*. *dixeyi* the anterior cervical neural spine is low and oriented posterodorsally (e.g., MAL-243, MAL-278-1 [3]; E.G. pers. obvs. 2014, 2015); (3) the transverse process on anterior dorsal vertebrae are oriented horizontally in *M*. *moyowamkia* rather than dorsolaterally as in *M*. *dixeyi* (MAL-238 [3]); (4) the articular surfaces of the prezygapophysis and postzygapophysis in dorsal vertebrae of *M*. *dixeyi* are significantly more pronounced than those in the dorsal vertebrae of *M*. *moyowamkia* (e.g., MAL-283, MAL-239); (5) the neural spine of the middle dorsal vertebrae in *M*. *moyowamkia* is significantly inclined posteriorly than those in *M*. *dixeyi*; (6) the neural spine in the middle dorsal vertebrae is more elongated in *M*. *dixeyi* than in *M*. *moyowamkia*; (7) anterior caudal vertebrae are weakly procoelous in *M*. *moyowamkia* whereas the anterior caudal vertebrae are strongly procoelous in *M*. *dixeyi*; (8); the caudal vertebrae lack a posterior haemal arch facet in *M*. *moyowamkia*; (9) the anterior caudal vertebrae exhibit a ventrolateral ridge in *M*. *dixeyi* that is absent in the recovered caudal vertebrae of *M*. *moyowamkia*; (10) the anterior caudal neural spines are relatively anteroposteriorly shorter in *M*. *moyowamkia* than those exhibited in *M*. *dixeyi*; (11) the middle–distal caudal vertebra in *M*. *moyowamkia* exhibits a uniquely pronounced dorsolateral expansion of the centrum whereas in *M*. *dixeyi* it is only slightly developed in MAL-197 8–10 (Fig 17); (12) the middle-distal caudal prezygapophysis is shorter (i.e., it barely extends farther anteriorly than the anterior face of the centrum) in *M*. *moyowamkia* than in *M*. *dixeyi* (i.e., it extends farther anteriorly than the anterior face of the centrum); (12) *M*. *moyowamkia* ischium exhibits a broad acetabular region rather than the nearly right angle condition that is exhibited in the ischium of *M*. *dixeyi* (MAL-183-1); (13) the head of the femur in *M*. *moyowamkia* is displaced father medially than the femur of *M*. *dixeyi* (MAL-201 [3]); (14) a more distinct cnemial crest is present on the tibia of *M*. *moyowamkia* than the tibia attributed to *M*. *dixeyi* (MAL-207); and (15) a thicker tibial midshaft in *M*. *moyowamkia* than in the tibia MAL-207 of *M*. *dixeyi*. Furthermore, there do not appear to be osteoderms associated with *M*. *moyowamkia* given the extent of the material recovered from the quarry, a peculiar anatomical feature present in *M*. *dixeyi* and other members within Lithostrotia (e.g., *M*. *shahinae* from the Late Cretaceous of Egypt [16]). However, there appear to be several characters that support the close relationship between *M*. *moyowamkia* and *M*. *dixeyi* as suggested in the tip-dating analysis: similar tooth morphology (e.g., tooth morph B of *M*. *moyowamkia*); spinoprezygapophyseal lamina of the anterior cervical vertebrae are kinked; presence of a median lamina from the interpostzygapophyseal lamina and the neural canal of the anterior dorsal vertebrae (but also present in *R*. *krausei*, *M*. *pecheni*, and few other titanosaurs); the presence of the posterolateral expansion of the middle-distal caudal centra (but also weakly present in the distal caudal vertebrae of the Late Cretaceous *L*. *pandafilandi*); and the distal width of the tibia near twice the midshaft width (but also present in *D*. *matildae* and several Late Cretaceous titanosaurians).

Despite these morphological differences, the phylogenetic distance among *M*. *moyowamkia*, *S*. *songwensis*, and *R*. *bisepultus* to *K*. *gittelmani* and *M*. *dixeyi* further supports the notion that the Dinosaur Beds of Malawi and the Galula Formation may only be partially coeval units, and as a consequence, that they would only share limited faunal similarity as had been previously postulated [4]. This may, in part, be a temporal bias as the Aptian assignment for the Dinosaur Beds is based on two fragmentary ostracodes and relatively coarse vertebrate biostratigraphy [34, 116]. This is clearly in need of rigorous re-evaluation and testing to determine if more precise ages can be obtained for direct correlation with the Galula Formation. As it stands, it is not surprising that *R*. *bisepultus* and *S*. *songwensis* are distinct from *M*. *dixeyi*, as these Namba Member titanosaurians are significantly younger in age, but the potential remains that *M*. *moyowamkia* and *M*. *dixeyi* were either sister-taxa or serially paraphyletic as the age of the Mtuka Member is best-constrained to the Aptian–Cenomanian [50]. Generally, both the Galula Formation (encompassing both Mtuka and Namba Members) and the Dinosaur Beds share similar higher order taxonomic overlap that includes titanosaurian sauropods (e.g., *M*. *dixeyi*, *R*. *bisepultus*), notosuchian crocodyliforms (e.g., *Malawisuchus mwakasyungutiensis*, *Pakasuchus kapilimai*), theropod dinosaurs, turtles, and osteichthyian fish. Yet, this is only part of the picture: such a faunal composition does not necessarily indicate that the two depositional units are similar, as these faunal components are also present in other Gondwanan faunas (e.g., northern Africa). In a recent mesoeucrocodyliform analysis [13], *P*. *kapilimai* (Namba Member) and *M*. *mwakasyungutiensis* were recovered as a paraphyletic grade to more exclusive ziphosuchians, and *Rukwasuchus yajabalijekundu* (Namba Member) was recovered with the circum-Saharan African peirosaurids *Hamadasuchus rebouli* and *Stolokrosuchus lapparenti*. The findings here and in the Sertich and O’Connor [13] study suggests that the relationship of sub-Saharan African faunas with adjacent regional faunas may be more complex and exhibit different signals that are clade dependent. The sub-Saharan African titanosaurians suggest a closer relationship with South American forms, with subclades within Notosuchia suggesting close relationship within sub-Saharan and South American forms (e.g., *Pakasuchus*, *Malawisuchus*) or with pan-African forms (e.g., *Rukwasuchus*, *Hamadasuchus*). Taken together, the recent discoveries from the Galula Formation of Tanzania suggest a potential mosaic of paleobiogeographic signals and interpretations for Cretaceous African faunas, signals that will no doubt continue to come into focus with the recovery of additional fossils from undersampled units.

## Supporting information

S1 File Supporting information. Supporting information containing the data used for phylogenetic analyses (taxa, stratigraphic ages, morphological character list and states, character modifications, and references).
(DOCX)

S2 File Bayesian nexus file. (NEX)

S3 File Parsimony nexus file. (NEX)

S4 File Bayesian equal rates tree file. (TRE)

S5 File Bayesian variable rates tree file. (TRE)

S6 File Tip dating equal rates tree file. (TREE)

S7 File Tip dating variable rates tree file. (TREE)

S8 File Parsimony tree file. (TRE)
