# Supplementary material for: A new African Titanosaurian Sauropod Dinosaur from the middle Cretaceous Galula Formation (Mtuka Member), Rukwa Rift Basin, Southwestern Tanzania
Source: PLoS One. 2019 Feb 13;14(2):e0211412. doi: 10.1371/journal.pone.0211412 (PMC6374010; doi:10.1371/journal.pone.0211412)
Supplement: S1 File — Supporting information containing the data used for phylogenetic analyses (taxa, stratigraphic ages, morphological character list and states, character modifications, and references). (DOCX) [file pone.0211412.s001.docx]

A new African Titanosaurian Sauropod Dinosaur from the middle Cretaceous Mtuka Member of the Galula Formation, Rukwa Rift Basin, Southwestern Tanzania

Eric Gorscak^1,2,3,4,5*^ and Patrick M. O’Connor^4,5^

^1^Department of Anatomy, Midwestern University, Downers Grove, Illinois, USA

^2^Integrative Research Center, Field Museum of Natural History, Chicago, Illinois, USA

^3^Department of Biological Sciences, Ohio University, Athens, Ohio 45701 U.S.A.

^4^Department of Biomedical Sciences, Ohio University Heritage College of Osteopathic Medicine, Athens, Ohio 45701 U.S.A.

^5^Ohio Center for Ecology and Evolutionary Studies, Ohio University, Athens, Ohio 45701 U.S.A.

*Corresponding Author

E-mail: eric.gorscak@gmail.com (EG)

S1 File: Supporting information

Sauropod data and methods are based from Gorscak and O’Connor (2016) and includes modifications and additions from Gorscak et al. (2017) and Sallam et al. (2018).

1. Taxon and Stratigraphic Information

2. Morphological Characters

3. Taxon/Character Modifications

4. References

1. Taxon and Stratigraphic Information. Taxon and Stratigraphic Information, in millions of years (Gradstein et al., 2012)

| *Taxon* | Upper | Lower | Reference |
| --- | --- | --- | --- |
| *Brachiosaurus* | 145 | 157.3 | Riggs, 1903; Wilson, 2002 |
| *Camarasaurus* | 145 | 157.3 | Cope, 1877; Wilson, 2002 |
| *Euhelopus* | 113 | 130.8 | Wiman, 1929; Wilson and Upchurch, 2009 |
| *Phuwiangosaurus* | 113 | 130.8 | Martin et al., 1994; Suteethorn et al., 2009, 2010 |
| *Malawisaurus* | 113 | 126.3 | Haughton, 1928; Jacobs et al., 1993; Gomani, 2005 |
| *Tapuiasaurus* | 113 | 126.3 | Zaher et al., 2011 ; Wilson et al., 2016 |
| *Tangvayosaurus* | 100.5 | 126.3 | Allain et al., 1999 |
| *Normanniasaurus* | 104.7 | 113 | Le Loeuff et al., 2013 |
| *Ligabuesaurus* | 100.5 | 119.7 | Bonaparte et al., 2006; D’Emic, 2012 |
| *Rukwatitan* | 72.1 | 77.9 | Gorscak et al., 2014 |
| *Andesaurus* | 97.2 | 113 | Calvo and Bonaparte, 1991; Mannion and Calvo, 2011 |
| *Argentinosaurus* | 93.9 | 113 | Bonaparte and Coria, 1993 |
| *Diamantinasaurus* | 93.9 | 100.5 | Hocknull et al., 2009; Poropat et al., 2015b |
| *Chubutisaurus* | 93.9 | 100.5 | del Corro, 1975; Carballido et al., 2011 |
| *Paralititan* | 93.9 | 100.5 | Smith et al. 2001 |
| *Epachthosaurus* | 91.9 | 100.5 | Powell, 1990; Martinez et al., 2004 |
| *Angolatitan* | 89.8 | 91.9 | Mateus et al., 2011 |
| *Futalognkosaurus* | 86.3 | 93.9 | Calvo et al., 2007b, c |
| *Mendozasaurus* | 88.1 | 91.9 | González Riga, 2003, 2005 |
| *Muyelensaurus* | 88.1 | 91.9 | Calvo et al., 2007a |
| *Rinconsaurus* | 86.3 | 91.9 | Calvo and Gonzalez Riga, 2003 |
| *Gondwanatitan* | 83.6 | 95.9 | Kellner and de Azevedo, 1999 |
| *Maxakalisaurus* | 83.6 | 95.9 | Kellner et al., 2006 |
| *Neuquensaurus* | 77.9 | 83.6 | Powell, 1986; Otero, 2010; D’Emic and Wilson, 2011 |
| *Overosaurus* | 72.1 | 83.6 | Coria et al., 2013 |
| *Aeolosaurus* | 66 | 83.6 | Santucci and de Arruda–Campos, 2011 |
| *Argyrosaurus* | 66 | 83.6 | Lydekker, 1893; Mannion and Otero, 2012 |
| *Panamericansaurus* | 69.1 | 77.9 | Calvo and Porfiri, 2010 |
| *Alamosaurus* | 66 | 77.9 | Gilmore 1922, 1946; D’Emic, 2012 |
| *Lirainosaurus* | 69.1 | 77.9 | Sanz et al., 1999;  Díez Díaz et al., 2011, 2012, 2013a, b |
| *Saltasaurus* | 69.1 | 77.9 | Bonaparte and Powell, 1980 |
| *Opisthocoelicaudia* | 69.1 | 72.1 | Borsuk–Bialynicka, 1977 |
| *Paludititan* | 69.1 | 72.1 | Csiki et al., 2010 |
| *Baurutitan* | 66 | 72.1 | Kellner et al., 2005 |
| *Isisaurus* | 66 | 72.1 | Jain & Bandyopadhyay, 1997;  Wilson and Upchurch, 2003 |
| *Rapetosaurus* | 66 | 72.1 | Curry Rogers & Forster, 2001; Rogers, 2004, 2009 |
| *Trigonosaurus* | 65.5 | 72.1 | Campos et al., 2005 |
| *Pellegrinisaurus* | 69.1 | 83.6 | Salgado, 1996 |
| *Dreadnoughtus* | 66 | 83.6 | Lacovara et al., 2014 |
| *Nemegtosaurus* | 68 | 70.1 | Nowinski, 1971; Wilson, 2005 |
| *Wintonotitan* | 93.9 | 100.5 | Hocknull et al., 2009; Poropat et al., 2015a |
| *Tastavinsaurus* | 119.7 | 126.3 | Canudo et al., 2008; Royo-Torres et al., 2009 |
| *Malarguesaurus* | 88.1 | 91.9 | González Riga et al., 2009 |
| *Ampelosaurus* | 69.1 | 77.9 | Le Loeuff, 1995; 2005 |
| *Bonitasaura* | 77.9 | 86.3 | Apesteguía, 2004; Gallina and Apesteguía, 2011, 2015 |
| *Jiangshanosaurus* | 100.5 | 113 | Tang et al., 2001 |
| *Karongasaurus* | 113 | 126.3 | Gomani, 2005 |
| *Daxiatitan* | 113 | 126.3 | You et al., 2008 |
| *Shingopana* | 72.1 | 77.9 | Gorscak et al., 2017 |
| *Atsinganosaurus* | 72.1 | 77.9 | Garcia et al., 2010 |
| *Lohuecotitan* | 69.1 | 77.9 | Díez Díaz et al., 2016 |
| *Mansourasaurus* | 72.1 | 83.6 | Sallam et al., 2018 |
| *Mnyamawamtuka* | 93.9 | 126.3 | Current Study |
| *Notocolossus* | 85.0 | 88.1 | González Riga, et al., 2016 |
| *Patagotitan* | 100.5 | 106.8 | Carballido et al., 2017 |

2. Morphological Characters

1. Snout shape: short, and deep (0); long and short (1). (modified from Upchurch, 1998; Curry Rogers, 2005; Gallina and Apesteguia, 2011)
2. Posterolateral and lateral processes of premaxilla and lateral process of maxilla, shape: without midline contact (0); with midline contact forming marked narial depression, subnarial foramen not visible laterally (1). (Wilson, 2002)
3. Premaxillary anterior margin shape: with step (0); without step (1). (polarity reversed; Wilson, 2002)
4. Maxilla, foramen anterior to the preantorbital fenestra: absent (0); present (1). (Zaher et al., 2011)
5. Jugal–maxillary process structure: jugal bluntly overlaps posterior border of maxilla (0); jugal has tongue-in-groove articulation with elongate maxillary process (1). (Curry Rogers, 2005)
6. Preantorbital fenestra, deep and large anteroposteriorly oriented fossa: opening directly on the lateral side of the maxilla or recessed in a small and shallow fossa (0); recessed into a deep and large anteroposteriorly oriented fossa (1). (Zaher et al., 2011)
7. Antorbital fenestra: much shorter than (0); or subequal to orbital maximum diameter (1). (Wilson, 2002)
8. External nares, configuration of lateral margin: Lacrimal excluded (0); lacrimal included (1). (Salgado et al., 1997)
9. Lacrimal, anterior process: absent (0); present (1). (polarity reversed; Wilson, 2002)
10. Lacrimal, anterior process: short, less than 50% of the length of the ventral process (0): long, at least 75% of the length of the ventral process (1). (Zaher et al., 2011)
11. Jugal, contribution to antorbital fenestra: very reduced or absent (0); large, bordering approximately one-third its perimeter (1). (Wilson, 2002)
12. Prefrontal, anterior process: absent (0); present (1). (Curry Rogers, 2005)
13. Prefrontal, width at the level of the frontal contact: large, equal or longer than the anteroposterior length of the prefrontal (0); narrow, less than half the anteroposterior length of the prefrontal (1). (Zaher et al., 2011)
14. Postorbital, posterior margin articulating with the squamosal: with tapering posterior process (0); with a deep posterior process (1). (Zaher et al., 2011)
15. Frontal, contribution to supratemporal fossa: absent (0); present (1). (Wilson and Sereno, 1998; Wilson, 2002)
16. Frontals, midline contact (symphysis): sutured (0); fused (1). (Salgado and and Calvo,1992; Upchurch, 1998; Wilson, 1998; Curry Rogers, 2005)
17. Frontal, medial convexity: absent (0); present (1). (Curry Rogers, 2005)
18. Parietal occipital process, dorsoventral height: deep, nearly twice the diameter of the foramen magnum (0); short, less than the diameter of the foramen magnum (1). (polarity reversed; Wilson, 2002)
19. Parietal, elongate lateral process: absent (0); present (1). (Curry Rogers, 2005)
20. Parietal, contribution to post-temporal fenestra: present (0); absent (1). (Wilson, 2002)
21. Parietal, distance separating supratemporal fenestrae: less than (0); or twice the long axis of supratemporal fenestra (1). (Wilson, 2002)
22. Postparietal foramen: absent (0); present (1). (Wilson ,1998; Curry Rogers, 2005)
23. Supratemporal fenestra: faces dorsally or dorsolaterally (0); faces anterodorsally (1). (Modified from Salgado and Calvo 1992; Upchurch 1998; Curry Rogers, 2005).
24. Squamosal, participation in supratemporal fenestra: includes (0); excludes (1). (Curry Rogers, 2005)
25. Quadratojugal, anterior process length: short, anterior process shorter than dorsal process (0); long, anterior process more than twice as long as dorsal process (1). (Wilson, 2002)
26. Ventral edge of anterior surface of the quadratojugal: straight, not expanded ventrally (0); concave due to a ventral expansion of the anterior region (1). (Upchurch et al., 2004)
27. Quadrate fossa, orientation: posterior (0); posterolateral (1). (Wilson, 2002)
28. Palatobasal contact, rocker-like surface for basipterygoid articulations: absent (0); present (1). (Modified from Wilson, 2002)
29. Pterygoid, sutural contact with ectopterygoid: on the lateral surface of the ectopterygoid (0); on the medial surface of the ectopterygoid (1). (Zaher et al., 2011)
30. Pterygoid, sutural contact with ectopterygoid: broad, along the medial or lateral surface (0); narrow, restricted to the anterior tip of the ectopterygoid (1). (Zaher et al., 2011)
31. Pterygoid, quadrate flange size: large, palatobasal and quadrate articulations well separated (0); small, palatobasal and quadrate articulations approach (1). (Wilson, 2002)
32. Paroccipital process shape and orientation: anteroposteriorly deep and posteroventrally oriented (0); anteroposteriorly shallow and laterally oriented (1). (Wilson ,1998; Curry Rogers, 2005)
33. Occipital condyle, orientation: posteroventrally (0); ventrally orientated (1). (Curry Rogers, 2005)
34. Basipterygoid process: short, approximately twice basal diameter (0); long, approximately four times basal diameter (1). (Wilson and Sereno, 1998; Curry Rogers, 2005)
35. Basipterygoid processes, angle of divergence: wide, approximately 45° or more (0); narrow, less than 30° (1). (Modified from Wilson, 2002)
36. Basipterygoid processes, orientation: perpendicular to (0); or angled approximately 45° to skull roof (1). (Wilson, 2002)
37. Basal tubera, anteroposterior depth: approximately half dorsoventral height (0); sheet-like, 20% dorsoventral height (1). (Wilson, 2002)
38. Basal tubera, angle of divergence: wide, greater than or equal to 60􏰁 degrees (0); narrow, less than 45􏰁 degrees (1). (Curry Rogers, 2005)
39. Basal tubera, position of divergence: occurs approximately level with occipital condyle or slightly below (0); occurs well below occipital condyle (1). (Curry Rogers, 2005)
40. Basioccipital depression between foramen magnum and basal tubera: absent (0); present (1). (Wilson, 2002)
41. Basisphenoid, sagittal ridge between basipterygoid processes: absent (0); present (1). (Zaher et al., 2011)
42. Basisphenoid/basipterygoid recess: present (0); absent (1). (Wilson, 2002)
43. Basisphenoid/quadrate contact: absent (0); present (1). (Wilson, 2002)
44. Supraoccipital, height: twice (0); or subequal or less than height of foramen magnum (1). (Wilson, 2002)
45. Supraoccipital, longitudinal groove: absent (0); present (1). (Curry Rogers, 2005; Gallina and Apesteguia, 2011)
46. Paroccipital process, ventral non-articular process: absent (0); present (1). (Wilson, 2002)
47. Mandible shape: U-shaped, curves towards symphysis (0); L-Shaped, dentary forms a right angle towards symphysis, rectangular (1). (Gallina and Apesteguia, 2011)
48. Dentary, depth of anterior end of ramus: more than depth of mid-length, 150% minimum depth (0); slightly less than that of dentary at mid-length (1). (Wilson, 2002; Curry Rogers, 2005)
49. Dentary symphysis, orientation: angled 45° or more anteriorly to (0); or perpendicular to axis of jaw ramus (1). (Wilson, 2002; Curry Rogers, 2005)
50. Dentary, alveoli/tooth count: 12 or more (0); 11 or less (1).
51. Dentary tooth rows, distribution relative to dentary length: at half or more (0); restricted to within anterior half, approaching roughly a third of length (1). (Curry Rogers, 2005)
52. Coronoid, size: reduced, not extending dorsal to splenial or absent (0); extending to dorsal margin of jaw (1). (Modified and polarity reversed, Wilson, 2002)
53. Tooth occlusal pattern: interlocking, V-shaped facets (0); high-angled planar facets (1). (Wilson, 2002)
54. Tooth crowns, orientation: aligned along jaw axis, crowns do not overlap (0); aligned slightly anterolingually, tooth crowns overlap (1). (Wilson, 2002)
55. Tooth crowns, slenderness index values (apicobasal height length of tooth crown divided by maximum mesiodistal width): less than (0); greater than (1) 4.0. (Upchurch 1998; Upchurch et al., 2004; Mannion et al., 2013)
56. Tooth crowns, cross-sectional shape at mid-crown: D-shaped, wide (0); cylindrical (1). (polarity changed, Wilson, 2002)
57. Tooth crowns, distinct mesial and distal carinae/ridges: absent (0); present (1). (Mannion et al., 2013)
58. Tooth serrations/denticles: present (0); absent (1). (Wilson, 2002; Upchurch et al., 2004; D’Emic, 2012; Mannion et al., 2013)
59. Enamel surface texture: wrinkled (0); smooth (1). (polarity reversed, Wilson, 2002)
60. Teeth, orientation: perpendicular (0); or oriented anteriorly relative to jaw margin (1). (Wilson, 2002; Curry Rogers ,2005)
61. Presacral bone texture: solid (0); spongy, with large open internal cells, camellate (1). (Wilson, 2002)
62. Number of cervical vertebrae: 13 or less (0); greater than 13 (1). (modified from Wilson, 2002)
63. Cervical pneumatopores (pleurocoels), shape: complex, divided by bony septa (0); simple, undivided (1). (polarity reversed, Wilson, 2002)
64. Axis: more than (0); less than (1) twice as long as tall. (D’Emic, 2012)
65. Postaxial cervical vertebrae, ventral keel: present (0); absent (1). (Curry Rogers, 2005; Mannion et al., 2013)
66. Cervical vertebrae, pendant parapophyses: absent (0); present (1). (D’Emic, 2012)
67. Cervical neural arches, ‘pre-epipophysis’ on prezygapophysis: absent, or weakly developed (0); present, well-developed, projecting anteriorly (1). (Mannion et al., 2013)
68. Middle and posterior cervical vertebrae, parapophysis: less than half functional length of centrum (0); half or more functional length of centrum (1). (D’Emic, 2012)
69. Shape of neural canal of cervical vertebrae: consistent (0); narrows mid-length (1). (Curry Rogers, 2005; D’Emic 2012)
70. Cervical neural arch lamination: well developed, with well defined laminae and coels (0); rudimentary, diapophyseal laminae only feebly developed if present (1). (Wilson, 2002)
71. Cervical vertebral epipophysis of postzygapophysis: absent or not well-developed (0); long, posteriorly projecting prongs (1). (D’Emic, 2012)
72. Anterior cervical centra, height:width ratio of anterior cervical centra: is roughly 1.0 or less (0); is approximately 1.25 (1). (Wilson, 2002)
73. Anterior cervical neural spines shape: single (0); bifid (1). (Wilson, 2002)
74. Mid cervical centra, anteroposterior length / height of posterior face: less than 4.0 (0); more than 4.0 (1). (Wilson, 2002)
75. Middle and posterior cervical neural arches, centroprezygapophyseal laminae (cprl), shape: single (0); divided (1). (Wilson, 2002)
76. Middle and posterior cervical vertebrae, postzygopophysis overhang: posterior margin of centrum (0); past the posterior margin of the centrum (1). (Powell, 1992)
77. Middle to posterior cervical vertebrae, neural spine progresses from posteriorly inclined to vertically oriented distinct projection atop the confluence of the spinoprezygopophyseal and spinopostzygopopyhseal laminae: absent (0); present (1).
78. Cervical vertebrae, ‘kinked’ intrapostzygapophyseal lamina: absent (0); present (1). (D’Emic, 2012)
79. Posterior cervical and anterior dorsal vertebrae with neural spines: longer (0); or shorter than centrum height (1). (D’Emic, 2012)
80. Posterior cervical and anterior dorsal vertebrae, separation between neural canal and interprezygapophyseal lamina: present, high neural arch (0); minimal-to-absent, low neural arch (1). (Modified from D’Emic, 2012; Bonaparte et al., 2006)
81. Posterior cervical and anterior dorsal neural spines shape: single (0); bifid (1). (Wilson, 2002)
82. Posterior cervical and anterior dorsal bifid neural spines, median tubercle: absent (0); present (1). (Wilson, 2002)
83. Posterior cervical and anterior dorsal axial region, neural spines, ‘laminar’ lateral expansion between spinoprezygo- and spinopostzygopopyhseal laminae, potentially an expansion of the spinodiapophyseal lamina: absent (0); present (1). (Modified from Calvo et al., 2007)
84. Posterior cervical and anterior dorsal axial region, neural spines, ‘bulbous’ lateral expansion: absent (0); present (1). (Campos et al., 2005)
85. Posterior cervical and anterior dorsal axial region, shape of the neural spine: taper along the length (0); expand distally and end in a rounded anteroposteriorly thin blade, ‘paddle-shaped’ (1). (D’Emic, 2012).
86. Posterior cervical vertebrae, prespinal lamina: absent or incipient (0); present (1). (Modified from Salgado et al., 1997; D’Emic, 2012; Mannion et al., 2013)
87. Number of dorsal vertebrae: more than 10 (0); 10 or fewer (1). (Modified from Wilson, 2002)
88. Anterior dorsal transverse process: project laterally (0); angled dorsolaterally (1). (Upchurch, 1998; Mannion et al., 2013)
89. Anterior dorsal neural arches, median infra-postzygapophyseal lamina or ridge: absent (0); present (1). (Curry Rogers, 2005)
90. Anterior dorsal vertebrae, prespinal lamina: absent (0); present (1). (Modified from Curry Rogers, 2005; D’Emic, 2012; Mannion et al., 2013)
91. Anterior dorsal vertebrae, prespinal lamina: partially present (0); persists entire length of neural spine (1). (Modified from Curry Rogers, 2005)
92. Anterior dorsal vertebrae, neural spines orientation: mostly vertical (0); posteriorly inclined (1). (Curry Rogers, 2005; Gallina and Apesteguia, 2011)
93. Anterior dorsal vertebrae, neural spine height: tall, exceeding height of transverse process and zygapophyses (0); short, barely taller or shorter than the transverse process and zygapophyses (1). (Modified from Curry Rogers, 2005)
94. Middle and posterior dorsal vertebral centra with ventral keel: absent (0); present (1). (D’Emic, 2012)
95. Middle dorsal vertebrae, posterior centrodiapophyseal lamina (pcdl): single (0); double, with low relief (1). (D’Emic, 2012)
96. Middle dorsal vertebrae, postzygapophyseal-diapophyseal lamina (podl): present (0); absent (1). (D’Emic, 2012)
97. Middle and posterior dorsal neural arches, centropostzygapophyseal lamina (cpol), shape: simple, undivided (0); divided (1). (Wilson, 2002)
98. Middle and posterior dorsal neural arches, posterior centroparapophyseal lamina (pcpl): absent (0); present (1). (Wilson, 2002)
99. Middle and posterior dorsal vertebrae, fossa dorsal neural canal and ventral to postzygapophysis: present (0); absent (1). (Curry Rogers, 2005)
100. Middle and posterior dorsal vertebrae, median vertical lamina below intraprezygapophyseal lamina; absent (0); present (1). (Curry Rogers, 2005)
101. Middle and posterior dorsal vertebrae, median vertical lamina below infrapostzygapophyseal lamina; absent (0); present (1). (Curry Rogers, 2005)
102. Middle and posterior dorsal vertebrae, diapophysis, distal end: dorsal surface grades smoothly towards midline (0); dorsal surface flat and set off from the rest of the diapophysis by a lip (1). (Sanz et al., 1999; D’Emic, 2012; Mannion et al., 2013)
103. Middle and posterior dorsal vertebrae, prespinal laminae: bifurcates proximally (0); single (1). (D’Emic, 2012)
104. Middle and posterior dorsal vertebrae, postspinal lamina: absent (0); present (1). (Modified from Curry Rogers, 2005)
105. Middle and posterior dorsal vertebrae, postspinal lamina: absent or partially present (0); present to entire length of the neural spine (1). (Modified from Curry Rogers, 2005)
106. Middle and posterior dorsal neural arches spinopostzygopophyseal lamina (spol) shape: divided (0); single (1). (polarity reversed, Wilson, 2002)
107. Middle and posterior dorsal neural spines, height (measured from prezygapophysis): greater than 50% total height of vertebra (0); less than or subequal to 50% total height of vertebra (1). (Modified from Curry Rogers, 2005)
108. Middle and posterior dorsal neural spines, shape: tapering or rounded (0); flared distally, with pendant, triangular lateral processes or ‘aliform’ processes (1). (Wilson, 2002)
109. Middle and posterior dorsal neural spines orientation: vertical or slightly posterodorsally inclined (0); strongly inclined posterodorsally, neural spine may approach level of diapophyses of the subsequent vertebra, ‘overlaps’ (1). (Wilson, 2002)
110. Posterior dorsal vertebral centrum: circular, subequal height and width (0); markedly wide, may approach twice the width as high (1).
111. Posterior dorsal transverse processes/diapophysis project: angled dorsally (0); angled horizontally (1). (Modified from Upchurch, 1998; Mannion et al., 2013)
112. Posterior dorsal vertebrae, diapophysis on transverse process positioned dorsal to parapophysis: absent, positioned posterior or posterodorsal to transverse process (0); present, transverse process vertically aligned with parapophysis (1). (Upchurch, 1998; Curry Rogers, 2005)
113. Posterior dorsal neural arches, postzygapophyseal-diapophyseal lamina (podl): present (0); absent (1). (Salgado et al., 1997)
114. Dorsal neural arches, hyposphene-hypantrum articulations: present (0); absent (1). (Wilson, 2002)
115. Sacral vertebrae, number: 5 (0); 6 or more (1). (Wilson, 2002)
116. Sacral vertebrae contributing to acetabulum: numbers 1–3 (0); numbers 2+ (1). (Wilson, 2002)
117. Sacral centra, lateral pleurocoel or very deep fossa: absent (0); present (1). (Upchurch, 1998; Mannion et al., 2013)
118. Last sacral vertebra, posterior articulation shape: flat or concave (0); convex (1).
119. Sacral ribs, dorsoventral length: high, extending beyond dorsal margin of ilium (0); low, not projecting beyond dorsal margin of ilium (1). (polarity reversed, Wilson, 2002)
120. Caudal vertebrae, number: more than 35 (0); 35 or fewer (1). (Wilson, 2002)
121. Caudal vertebrae, centrum shape: cylindrical (0); squared, ventral and lateral margins relatively flat (1). (Modified from Wilson, 2002; Curry Rogers, 2005)
122. Apex of convexity of posterior articulation in anterior caudal vertebrae: absent or concentric (0); displaced dorsally (1). (Modified from Powell, 1986; Santucci Arruda-Campos, 2011)
123. Restricted articular condyle of procoelous vertebrae of the anterior-middle and onward caudal vertebrae: absent or condyle merges into outer margins (0); present, outer margin distinct from condyle (1). (Modified from Sanz et al., 1999)
124. Anterior and middle caudal centra, ventral surface bounded by ridges connecting the areas for anterior and posterior haemal arch articulations (i.e., ventrolateral ridges): weak or absent absent (0); developed and present (1). (Modified from Wilson, 2002)
125. Caudal transverse processes: continue posteriorly through caudal 10 (0); disappear by or at caudal 10 (1). (Modified from Wilson, 2002)
126. Caudal transverse process, placement upon vertebra: on the centrum, at or below dorsal margin (0); above the dorsal margin of the centrum, on level of the neural canal (1).
127. Caudal transverse process, curvature: weak, laterally oriented (0); strong, curves posterolaterally (1). (Mannion and Calvo, 2011; Mannion et al., 2013)
128. Caudal transverse process: extends prior to the posterior margin of the centrum, excluding the condyle, if present (0); extends at or beyond the posterior margin of the centrum, excluding the condyle, if present (1). (Mannion and Calvo, 2011; Mannion et al., 2013)
129. Anterior and middle caudal centrum: dorsoventrally elongated/taller than wide or subequal height/widths (0); markedly compressed dorsoventrally, wider than tall (1). (Salgado et al., 1997; Upchurch et al., 2004)
130. Anterior caudal and middle caudal vertebrae (excluding anteriormost), ventral longitudinal groove: absent (0); present (1). (McIntosh, 1990; Upchurch, 1995, 1998; Wilson, 2002; Mannion et al., 2013)
131. Anterior and middle caudal vertebrae, neural arches, location: over the midpoint of the centrum with approximately subequal amounts of the centrum exposed at either end (0); within the anterior half of the centrum (1). (Modified from Salgado et al., 1997; Upchurch et al., 2004.)
132. Articular facets of the prezygapophyses on anterior and middle caudal vertebrae: (0) normal, not expanded; (1) wide, with a dorsal and a ventral expansion or protuberance. (Powell, 1986; Santucci and Campos, 2011)
133. Postzygapophyses located above the anterior half of the centrum on anterior and middle caudal vertebrae: located above posterior half (0); present (1). (Salgado et al., 1997; Santucci and Campos, 2011)
134. Anterior and middle caudal postzygapophysis articular surface, curvature: mainly straight or weakly curved (0); markedly curved (1).
135. Anterior and middle caudal vertebrae, postzygapophysis in relation to neural spine: mostly placed on the neural spine (0); projecting off the neural spine posteriorly, significantly past the posterior margin of the neural spine (1).
136. Anterior and middle caudal vertebrae, anterior extension of the anterodorsal corner of the neural spine: absent (0); present, distal end anteroposteriorly elongated relative to proximal portion of spine (1).
137. First caudal centrum, biconvex: no (0); yes (1). (Modified from Wilson, 2002)
138. Anterior caudal centra (excluding the first), anterior articulation: nearly flat or convex (0); concave (1). (Modified from Wilson, 2002)
139. Anterior caudal vertebral centra, ventrolaterally facing surfaces: absent, maintain a cylindrical or box-like shape (0); present, constricting the ventral surface (1). (Modified from Salgado and Garcia, 2002; D’Emic and Wilson, 2011)
140. Anterior caudal centra (excluding the first), posterior articulations: nearly flat or concave (0); convex, condyle (1). (Modified from Wilson, 2002)
141. Anterior margin of the anterior caudal vertebral centrum: vertical (0); inclined forward (1). (Santucci and Arruda-Campos, 2011)
142. Anterior caudal vertebrae, location of neural arch: at or within the anterior half (0); strongly present anteriorly, flush or nearly flush with the anterior margin of the centrum (1).
143. Anterior caudal vertebrae, dorsal tuberosity/ridge placed along posterolateral surface of the pedicle/between transverse process and neural arch: absent (0); present (1). (Kellner et al., 2005)
144. Prezygapophyses curved downward on anterior caudal vertebrae: absent, straight (0); present (1). (Santucci and Arruda-Campos, 2011)
145. Anterior caudal vertebrae (excluding the anteriormost), prezygapophysis project: anteriorly (0); anterodorsally (1). (Curry Rogers, 2005)
146. Anterior and middle caudal vertebrae, tubercle or subtle blade-like process on spinoprezygapophyseal lamina near prezygapophysis: absent (0); present (1). (D’Emic, 2012)
147. Anterior caudal vertebral neural arch, hyposphenal ridge: present (0); absent (1). (Upchurch, 1998; Mannion et al., 2013)
148. Anterior caudal neural arches, postspinal fossa: absent (0); present (1). (Wilson, 2002)
149. Anterior caudal neural spine inclination: posterodorsally (0); vertical or anteriorly inclined (1). (modified from Gallina and Apesteguia, 2011)
150. Shape of the distal neural spines relative to proximal portion of anterior caudal vertebrae: do not expand transversely, less than 50% anteroposterior width (0); markedly expanded transversely, at or more than 50% anteroposterior length (1). (Modified from Wilson, 2002; Calvo et al. 2007; Gallina and Apestguia, 2011)
151. Anterior caudal vertebrae, prominently rugose and developed prespinal and postspinal laminae: absent or weakly developed (0); present (1).
152. Thin, longitudinal laminae along neural spine diverging from anterior median lamina (prespinal lamina) and postspinal laminae in anterior caudal vertebrae: absent (0); present (1). (Gallina et al., 2015; previously an autapomorphy for *Bonitasaura* but has since been recognized in *Patagotitan*).
153. Apex of convexity of posterior articulation in middle caudal vertebrae: absent or concentric (0); displaced dorsally (1). (Modified from Powell, 1986; Santucci and Arruda-Campos., 2011)
154. Middle caudal vertebra, lateral ridge at neurocentral juncture: absent (0); present (1). (Salgado et al., 1997)
155. Middle caudal vertebrae, neural arch location: within the anterior half (0); strong presence on anterior margin (1).
156. Middle–distal caudal centrum, posterolateral projections: absent (0); present, weakly-strongly developed (1) (Current study: note *Mnyamawamtuka* has strongly developed projections whereas *Malawisaurus* and *Lohuecotitan* have weakly developed projections)
157. Length proportions of the prezygapophyses with respect to the centrum length in middle caudal vertebrae: less than 50% (0); roughly at or more than 50% (1). (Santucci and Arruda-Campos, 2011)
158. Well developed interprezygapophyseal lamina in middle caudal vertebrae: weakly developed, absent (0); developed and anteriorly extended, present (1) (Calvo et al. 2007)
159. Postzygapophyseal process in middle caudal vertebra: absent (0); present (1) (Calvo et al. 2007; Gallina and Apestguia, 2011)
160. Middle caudal neural spines, orientation: angled posterodorsally (0); vertical or anterodorsally (1). (Modified from Wilson, 2002)
161. Middle caudal centra, procoelous: absent, weakly developed (0); present (1). (Modified form Wilson, 2002)
162. Distal caudal centra, procoelous: absent, weakly developed (0); present (1). (Modified from Wilson ,2002)
163. Middle to distal caudal centra, shape: subequal height and width or tall (0); dorsoventrally flattened, breadth up to twice height (1). (Wilson, 2002)
164. Cervical ribs, anterior projection extension: short, approximately the contact of the condyle and centrum, not extending towards the anterior margin of the condyle (0); long, extend at or past the anterior margin of the condyle (1).
165. Cervical ribs, shaft length: overlapping as many as three subsequent vertebrae (0); longer than centrum but not overlapping subsequent vertebrae(1). (polarity reversed, Wilson, 2002)
166. Dorsal ribs, proximal pneumatopores: absent (0); present (1). (Wilson, 2002)
167. Laminar projection on the anterior and posterior border of the anterior dorsal ribs. (Coria et al., 2013; Previously proposed as an autapomorphy for *Overosaurus*)
168. Forked chevrons with anterior and posterior projections: present (0); absent (1). (polarity reversed, Wilson, 2002)
169. Chevrons with double articular facets set in a concave posterodorsal surface: absent (0); present (1). (Santucci and Arruda-Campos, 2011)
170. Anterior and middle caudal vertebral chevrons, proximal articular facets: undivided (0); divided into anterior and posterior segments (1). (D’Emic, 2012)
171. Chevron haemal canal, depth: short, approximately 25% (0); or long, approximately 50% or more chevron length (1). (Wilson, 2002)
172. Posterior chevrons, distal contact: fused (0); unfused (open) (1). (Wilson, 2002)
173. Scapular acromion process, dorsoventral length to minimum scapular blade length (scapula orientation: the long axis of the blade is oriented perpendicular to the ground, coracoid articulation facing the ground): broad, more than 150% (0); narrow, less than 150% (1). (polarity reversed and modified from Wilson and Sereno, 1998; Wilson, 2002)
174. Scapula, posterior margin of the acromion: straight and oriented vertically or sloping and facing posterodorsally (0); concave, posterodorsal corner of acromion overhangs the dorsal surface of the scapular blade (1). (Rauhut et al., 2005; Mannion 2009; Mannion et al., 2013)
175. Scapular spine: perpendicular to long axis of scapula (0); gently curved, not perpendicular to long axis of scapula (1). (Curry Rogers, 2005)
176. Scapular blade, orientation respect to coracoid articulation: At or nearly perpendicular (0); forming a 45° angle (1). (Wilson, 2002; Curry Rogers, 2005)
177. Scapular blade, shelf-like extension along dorsal (acromion) margin: present (0); absent, either maintains subequal width or gently expands (1). (Modified from Wilson, 2002)
178. Scapular blade, distal end: flared and expanded relative to proximal end (0); subequal or narrower than proximal end (1). (Modified from Curry Rogers, 2005)
179. Scapulocoracoid suture: suture ends before dorsal margin of acromion (0); suture extends to dorsal margin of acromion and coracoid (1). (D’Emic, 2012)
180. Dorsal margin of scapula and coracoid: embayed, clear distinction between the two (0); flush, dorsal margins continuous (1). (Modified from D’Emic, 2012)
181. Scapula, process(es) on ventral margin near proximal base of blade: absent (0); present (1). (modified from D’Emic, 2012).
182. Tubercle present on the dorsomedial surface of the proximal scapular blade, posteroventral to the acromion process: absent (0); present (1). (Modified from Salgado et al., 1997; Sanz et al., 1999)
183. Scapula, medial surface of blade, ventral ridge or rugosity: absent (0); present (1). (Sanz et al., 1999)
184. Scapular glenoid orientation: anteroventrally or laterally facing (0); beveled medially (1). (Wilson, 2002)
185. Scapular blade, cross-sectional shape at base: flat or rectangular (0); D-shaped (1). (Wilson, 2002)
186. Glenoid, relative contribution from scapula and coracoid: subequal or coracoid contributes more (0); scapula contribute more (1). (Modified from Curry Rogers, 2005)
187. Coracoid, proximodistal length: subequal to or less than the length of scapular articulation (0); greater or approximately twice the length of scapular articulation (1). (Wilson, 2002)
188. Coracoid, anteroventral margin shape: rounded (0); rectangular (1). (Wilson, 2002)
189. Coracoid, anterodorsal margin shape: smoothly merges, rounded (0); rectangular (1). (Upchurch, 1998; Mannion et al., 2013)
190. Coracoid, infraglenoid lip: absent (0); present, concave ‘notch’ between glenoid and anterior margin (1). (Wilson, 2002)
191. Position of coracoid foramen: deeply inset into coracoid body, fully enclosed within coracoid (0); at margin of coracoid body, patent with margin and scapulocoracoid suture (1). (Modified from Curry Rogers, 2005)
192. Sternal plate, shape: oval (0); crescentic (1). (Wilson, 2002)
193. Sternal plate relative to humerus: 50% length (0); 70% length (1). (D’Emic, 2012)
194. Sternal plate, anteroventral ridge/projection near coracoid articulation: absent (0); present (1). (Sanz et al., 1999; Mannion et al., 2013)
195. Humeral proximomedial corner: straight or slightly curved (0); sigmoidal, proximomedial corner displaced dorsally relative to proximolateral corner. (Modified from Gonzalez Riga, 2003).
196. Humeral proximolateral corner, shape: rounded (0); square (1). (Wilson, 2002)
197. Humerus, posterolateral bulge at level of deltapectoral crest: absent (0); present (1). (D’Emic, 2012)
198. Humeral deltopectoral crest, shape: relatively narrow throughout length (0); markedly expanded distally (1). (Wilson, 2002)
199. Humerus, deltopectoral crest length along element: less than halfway (0); at or more than half (1). (Curry Rogers, 2005)
200. Humerus, deltopectoral crest: extends anteriorly (0); extends anteromedially (1). (Upchurch et al., 2004).
201. Humerus, lateral margin of diaphysis (approximately the middle third of the humerus): concave (0); straight (1) (Curry Rogers, 2005; Mannion et al., 2013).
202. Humerus supracondylar fossa: flat or shallow (0); deep and bounded by medial and lateral condylar ridges (1). (Upchurch et al., 2004; Mannion et al., 2013)
203. Humeral medial (ulnar) distal condyle, articular surface shape: restricted to distal portion of humerus (0); developed, exposed on anterior and posterior portion of humeral shaft (1). (Wilson, 2002)
204. Humeral lateral (radial) distal condyle, articular surface shape: restricted to distal portion of humerus (0); developed, exposed on anterior and posterior portion of humeral shaft (1). (Modified from Wilson, 2002)
205. Humerus, lateral (radial) condyle: divided (0); undivided (1). (D’Emic, 2012)
206. Humeral distal condyles, shape: flat (0); divided (1). (polarity reversed, Wilson, 2002)
207. Ulnar proximal condylar processes: subequal (0); unequal, anteromedial arm longer (1). (Wilson and Sereno, 1998; Curry Rogers, 2005)
208. Ulnar olecranon process, development: rudimentary, level with proximal articulation (0); prominent, projecting above proximal articulation (1). (polarity reversed, Wilson, 2002)
209. Ulna, articular surface of anteromedial process: flat (0); concave along its length (1). (Upchurch, 1995, 1998; Mannion et al., 2013)
210. Ulna, length-to-proximal breadth ratio: gracile, ratio of proximal width to ulnar length around 25% (0); stout, ratio of proximal width to ulnar length around 33% (1). (Wilson, 2002; Curry Rogers, 2005)
211. Ulna, distal end: prominently expanded posteriorly (0); unexpended (1). (D’Emic, 2012; Mannion et al., 2013)
212. Radius, proximal articular surface shape: subequal or transversely expanded (0); triangular, anteroposteriorly elongated (1). (Modified from Curry Rogers, 2005)
213. Radius, well-defined interosseous ridge: absent or weakly developed (0); present (1). (Curry Rogers, 2005)
214. Radius, distal breadth: slightly larger than midshaft breadth (0); approximately twice midshaft breadth (1). (Wilson, 2002)
215. Radius, distal condyle orientation: perpendicular to long axis of shaft (0); beveled approximately 20° proximolaterally, relative to long axis of shaft (1). (Wilson, 2002)
216. Carpal bones, number: 3 or more (0); 2 or fewer (1). (Wilson, 2002)
217. Metacarpals, relative lengths: MTC I is shorter than MTC III (0); MTC I is equal to or longer than MTC III (1). (Wilson, 2002; D’Emic, 2012)
218. Metacarpal I, distal third of the element: nearly straight (0); bowed medially (1). (Mannion et al., 2011)
219. Metacarpal I, distal condyle shape: divided (0); undivided (1). (Wilson, 2002)
220. Metacarpal I distal condyle, transverse axis orientation: beveled approximately 20° respect to axis of shaft (0); proximodistally or perpendicular with respect to axis of shaft (1). (Wilson, 2002)
221. Metacarpal V proximal end relative size to metacarpal I: subequal (0); smaller (1). (polarity reversed, D’Emic, 2012)
222. Phalanges: present (0); absent or unossified (1). (Wilson, 2002)
223. Manual phalanx I, shape: rectangular (0); wedge-shaped (1). (Wilson, 2002)
224. Ilium internal texturing, features camellate texturing: absent (0); present (1). (Wilson and Upchurch, 2009; Mannion et al., 2013)
225. Iliac preacetabular process, orientation: anterolateral to body axis (0); perpendicular to body axis (1). (Wilson, 2002)
226. Iliac preacetabular process, shape: pointed, arching ventrally (0); semicircular, with posteroventral excursion of cartilage cap (1). (Wilson, 2002)
227. Ilium, ventral preacetablum margin with kink: absent (0); present (1). (D’Emic, 2012)
228. Ilium, pubic peduncle projection with respect to sacral axis: acute angle (0); perpendicular (1). (Salgado et al., 1997; Curry Rogers, 2005)
229. Ilium, pubic peduncle, shape: anteroposterior and transverse dimensions subequal (0); transversely expanded (1). (D’Emic, 2012)
230. Ilium, pubic peduncle medial margin: present (0); acute, anterior and posterior margins converge, triangular (1).
231. Ilium, pubic peduncle contribution to acetabulum relative to ischial peduncle: equal or less than half (0); more than half (1). (Curry Rogers, 2005)
232. Pubis, acetabular and ischial surfaces: meet roughly perpendicular (0); obtuse (1). (Curry Rogers, 2005)
233. Pubis, puboischial contact length: one-half total length (0); markedly less than half, may approach approximately one-third total length (1). (polarity reversed and modified from Wilson, 2002)
234. Pubis, obturator foramen, shape: circular (0); elliptical (1). (Mannion et al., 2013)
235. External surface of pubis, longitudinal ridge: absent (0); present (1). (Calvo et al., 2007)
236. Pubis total length relative to ischium total length: equal or shorter (0); longer (1). (Salgado et al., 1997)
237. Pubis, distal end relative to midshaft: unexpanded (0); expanded (1). (Modified from Curry Rogers, 2005)
238. Ischium, iliac pedicle articular dimensions: transversely narrow or subequal (0); transversely wide and developed (1) (Modified from Calvo and González Riga 2003; Gallina and Apesteguia, 2011).
239. Ischial margin of acetabulum: obtuse angle (0); right or acute angle (1). (Modified from D’Emic, 2012; character divided into two)
240. Ischium, iliac peduncle general shape: short, robust (0); elongated, slender (1). (Modified from Calvo and González Riga, 2003; D’Emic, 2012; character divided into two)
241. Ischium, pubic peduncle: restricted, minor anterior presence (0); prominent, extended anteriorly, increased acetabular contribution (1). (Modified from Wilson, 2002; D’Emic, 2012)
242. Ischium, general blade shape: elongate and slender (0); short and blunt (1).
243. Ischial blade, emargination distal to pubic peduncle: present (0); absent (1). (Salgado et al., 1997; Curry Rogers, 2005)
244. Femur, proximolateral margin: angular corner, pronounced greater trochanter (0); gently curved corner into the lateral bulge, reduced greater trochanter (1).
245. Femur, femoral head in relation to medial margin: extends medially, angular with respect to medial margin (0); extends dorsomedially, wide angle with respect to medial margin (1).
246. Femoral midshaft, transverse diameter: 125-185% anteroposterior diameter (0); at least 185% anteroposterior diameter (1). (Modified from Wilson, 2002)
247. Femur, distinct longitudinal ridge on anterior surface: absent (0); present (1). (Otero, 2010; D’Emic, 2012)
248. Femoral shaft, lateral margin shape: straight (0); proximal one-third deflected medially (1). (Wilson, 2002)
249. Femoral distal condyles, relative transverse breadth: tibial much broader than fibular (0); tibial narrower or subequal to fibular (1). (polarity reversed, Wilson, 2002)
250. Femoral distal condyles, orientation: perpendicular or slightly beveled dorsolaterally (0); or beveled dorsomedially approximately 10° relative to femoral shaft (1). (Wilson, 2002)
251. Femoral distal condyles, articular surface shape: restricted to distal portion of femur (0); expanded onto anterior portion of femoral shaft (1). (Wilson, 2002)
252. Tibial proximal condyle, shape: expanded transversely or subcircular (0); narrow, long axis anteroposterior (1). (polarity reversed, Wilson, 2002)
253. Tibial cnemial crest, orientation: projecting anterior-anterolaterally; (0); laterally (1). (Wilson, 2002; Curry Rogers, 2005; Mannion et al., 2013)
254. Tibia, distal breadth: approximately 125% (0); near or more than twice midshaft breadth (1). (Wilson, 2002)
255. Fibula, shaft: straight or mildly sigmoidal (0); markedly sigmoidal, proximal portion displaced anteriorly relative to distal end (1). (Canudo et al., 2008; Royo-Torres, 2009; Mannion et al., 2013)
256. Fibula lateral trochanter: absent or weakly developed (0); prominent (1). (Powell, 1992; Upchurch, 1998; Mannion et al., 2013)
257. Fibula, muscular scar about mid-length of lateral surface: absent or oval (0); ridge- or crest-like and subparallel to long axis of element (1). (Curry Rogers, 2005)
258. Fibula distal condyle, relative width to midshaft: subequal (0); expanded transversely, greater than midshaft (1). (Wilson and Sereno, 1998; Curry Rogers, 2005)
259. Astragalus, posterior fossa shape: divided (0); undivided (1). (polarity reversed, Wilson, 2002)
260. Astragalus, posterior end of proximal face, tubercle: present (0); absent (1). (D’Emic, 2012)
261. Astragalus, transverse length: 50% more than (0); or subequal to proximodistal height (1). (Wilson, 2002)
262. Calcaneum: present (0); absent or unossified (1). (Wilson, 2002)
263. Metatarsal I distal condyle, transverse axis orientation: angled dorsomedially to axis of shaft (0); perpendicular (1). (polarity reversed, Wilson, 2002)
264. Metatarsal I distal condyle, posterolateral projection: absent (0); present (1). (Wilson, 2002)
265. Metatarsal III length divided by metatarsal I length: less than 1.3 (0); more than 1.3 (1). (González Riga et al., 2016:character number 331)
266. Longest metatarsal: metatarsal III (0); metatarsal IV (1). (González Riga et al., 2016:character number 334)
267. Pedal digit I ungual, length relative to pedal digit II ungual: 25% larger than that of digit II (0); subequal (1). (polarity reversed, Wilson, 2002)
268. Pedal digit I ungual, length: longer (0); or shorter (1) than metatarsal I. (polarity reversed, Wilson, 2002)
269. Number of phalanges in pedal digit II: 3 (0); 2 (1). (González Riga et al., 2016:character number 348)
270. Number of phalanges in pedal digit III: 4 (0); 3 (1). (González Riga et al., 2016:character number 349)
271. Number of phalanges in pedal digit IV: 2 or more (0); 1 (1). (González Riga et al., 2016:character number 350)
272. Osteoderms: absent (0); present (1). (Wilson, 2002)

*Camarasaurus* (Cope, 1877; D’Emic, 2012)

1. Lacrimal with long axis directed anterodorsally.
2. Quadratojugal with short anterior ramus that does not extend anterior to the laterotemporal fenestra.
3. Quadratojugal anterior process shorter than dorsal process.
4. Pterygoid with dorsomedially orientated basipterygoid hook.
5. Basal tubera width more than 1.6 times occipital condyle width.
6. Splenial posterior process separating anterior portions of angular and prearticular.
7. Conspicuous groove passing anteroventrally from the surangular foramen to the ventral margin of the dentary.
8. Forked chevrons restricted to distal tail.
9. Metacarpal V proximal end subequal in size to proximal end of metacarpal I.
10. Ischial blade directed posteriorly so that the long axis of its shaft passes though the pubic peduncle

*Brachiosaurus* *altithorax* (D’Emic, 2012)

1. Posterior dorsal vertebral column long relative to humerus length

*Euhelopus zdanskyi* (Wiman, 1929; D’Emic, 2012)

1. Maxillary ascending process flush with anterior margin of bone; subnarial fossa reduced.
2. Teeth procumbent with asymmetrical crown-root margin (i.e. the mesial margin closer to the apex of the crown).
3. Third cervical vertebral neural spine with laterally compressed, anteriorly projecting triangular process.
4. Anterior cervical vertebrae with three costal spurs on tuberculum and capitulum.
5. Cervical vertebrae with thin, horizontally orientated epipophyseal–prezygapophyseal lamina.

*Phuwiangosaurus sirindhornae* (Martin et al., 1994; D’Emic, 2012)

1. Quadrate with kinked posteromedial margin of quadrate fossa.
2. Axial centrum without paramedian fossae on anterior half of ventral centrum.
3. Posterior cervical and anterior dorsal vertebrae with tubercle on prdl.
4. Middle dorsal vertebrae with dorsally acuminate pneumatic openings.
5. Middle caudal vertebrae with kinked neural arch pedicle below postzygapophyses.

*Tangvayosaurus hoffeti* (Allain et al., 1999; D’Emic, 2012)

*Angolatitan adamastor* (Mateus et al., 2011)

1. Acute medioproximal margin of humerus.
2. Proximal outline of the ulna with anteromedial ridge (process).
3. Posterior facet of the distal epiphysis of metacarpal I with two small splint-like projections.

*Andesaurus delgadoi* (Calvo and Bonaparte, 1991; Mannion and Calvo, 2011)

1. Posterior dorsal neural spine height greater than twice the centrum height (autapomorphic within Macronaria).
2. Anterior–middle caudal centra square shaped in lateral view.
3. Anteroposteriorly elongate, elliptical fossa present on the anterior half of the lateral surface of the centrum of middle– posterior caudal vertebrae, close to the dorsal margin.
4. Ridge along the mishaft of the ventral surface of metacarpal I, close to the ventromedial margin.
5. Prominent ventromedial ridge along the distal half of metacarpal V.

*Rukwatitan bisepultus* (Gorscak et al., 2014)

1. Posterior cervical vertebrae with weakly developed carotid processes.
2. Posterior cervical vertebrae with paired, shallow, ventral fossa positioned anteriorly.
3. Elongate fossa deeply invading ventral surface of diapophysis of cervical vertebra.
4. Accessary tubercle on ventral surface of cervical rib capitulum.
5. Deep coracobrachialis fossa of the humerus.
6. Subquadrangular cross-section of the humerus.
7. Posteriorly curving, tear-drop shaped pubis peduncle of the ilium.

*Malawisaurus dixeyi* (Haughton, 1928; D’Emic, 2012)

1. Abbreviate premaxillary portion of snout, dentary arched ventrally.
2. Surangular notch and groove on dentary.

*Diamantinasaurus matildae* (Hocknull et al., 2009)

1. Intermediately robust humerus.
2. Metacarpal III phalange heavily reduced.
3. Cnemial crest projects cranially then laterally.
4. Intermediately robust fibula.

*Tapuiasaurus macedoi* (Zaher et al., 2011)

1. Hook-shaped posteroventral process of the quadratojugal.
2. Anterior process of the jugal tapering and forming most of the ventral margin of the antorbital fenestra.
3. Anterolateral tip of the pterygoid contacts the medial surface of the ectopterygoid.

*Isisaurus colberti* (Jain & Bandyopadhyay, 1997; Wilson and Upchurch, 2003)

1. Cervical centra broader than long.
2. Anteroposteriorly elongate cervical parapophyses.
3. Cervical neural arches with prespinal and postspinal laminae.
4. Cervical neural arches with divided cpol.
5. Anteriormost dorsal vertebra with pronounced coel between prezygodiapophyseal.(prdl), centroprezygapophyseal (cprl), and anterior centrodiapophyseal laminae (acdl).
6. Posterior dorsal neural arches with parapophyses positioned above level of prezygapophyses.
7. Anteroposteriorly compressed distal caudal chevron blades.

*Opisthocoelicaudia skarzynskii* (Borsuk-Bialynicka, 1977; Wilson, 2002)

1. Dorsal neural arches with enlarged coel between posterior centrodiapophyseal lamina (pcdl), spinodiapophyseal lamina (spdl), and centropostzygapophyseal lamina (cpol).
2. Anterior caudal prezygapophyses and postzygapophyses connect above transverse process.
3. Anterior caudal chevrons 1–4 unfused distally.
4. Chevrons disappearing after caudal 19.
5. Scapulocoracoid strongly arched medially.
6. Femoral fourth trochanter positioned distal to midshaft.

*Rapetosaurus krausei* (Curry Rogers & Forster, 2001; Rogers, 2009)

1. Antorbital fenestra large, extending anteriorly over tooth row.
2. Preantorbital fenestra positioned posterior to antorbital fenestra.
3. Anteriorly located, elongate subnarial foramen.
4. Narrow, posterodorsally elongate maxillary jugal process.
5. Frontal with median dome.
6. Quadrate with V-shaped quadratojugal articulation.
7. Supraoccipital with two anteriorly directed median parietal processes.
8. Pterygoid anterior process dorsoventrally expanded.
9. Basipterygoid articulation of pterygoid extremely shallow.
10. Basipterygoid processes divergent only at distal ends.
11. 11 alveoli extending for two-thirds the length of the dentary.
12. 43 precaudal vertebrae (17 cervical, 10 dorsal, 6 sacral vertebrae).
13. Prespinal lamina in anterior cervical vertebrae.
14. Dorsal vertebrae with high neural spine, ~40% vertebral height.
15. Dorsal vertebrae with intrapostzygopophyseal lamina bearing ventral extension and weblike pre- and postspinal laminae.
16. Sacral neural spines comprise half of total sacral vertebral height.
17. Mid-caudal neural spines taller than centrum, transversely expanded, anteroposteriorly reduced.
18. Mid-posterior caudal vertebrae with well developed pre- and postspinal lamina in shallow fossae.
19. Coracoid with oblique lateral ridge.
20. Humeral head level with proximal deltopectoral crest.
21. Straight distal humeral diaphysis.
22. Shelf-like iliac peduncle of ischium and pubis.
23. Femur strongly constricted at mid-diaphysis, gracile.

*Saltasaurus* *loricatus* (Bonaparte & Powell, 1980; Wilson, 2002)

1. Frontal with bulge near mid-orbit.
2. Cervical prezygapophyses low and wide.
3. Cervical parapophyses broad anteroposteriorly and extending the length of centrum.
4. Cervical ribs with tuberculum–capitulum angle less than 30°.
5. Acetabulum facing ventrolaterally and broadening anteriorly.
6. Pubis with small contribution to acetabulum.
7. Femur with vertically oriented posterior crest on proximal half of shaft.

*Neuquensaurus* (Powell, 1986; D’Emic and Wilson, 2011)

1. Sacral vertebral column length ~1.5 times the width between sacricostal yokes.
2. Sacral centra 4 and 5 less than half the width of the last sacral centrum.
3. Seven sacral vertebrae.
4. Prezygapophysis of middle caudal vertebrae with a non-articulating anterior process.
5. Longitudinal ridge below transverse process of middle caudal vertebrae.
6. PODL present and elongate on middle caudal vertebrae.
7. Fibula rotated anteromedially.

*Alamosaurus sanjuanensis* (Gilmore (1922; D’Emic, 2012)

1. Biconvex first caudal vertebra with circumferential depression on anterior condyle limited to ventral half of bone.
2. Cross-section of scapular blade asymmetrical, thicker ventral margin.
3. Ventral edge of scapular blade nearly straight, dorsal edge expanded distally.

*Paralititan stromeri* (Smith et al. 2001)

1. Prominent tabular process on caudoventral margin of scapula distal to the proximal expansion.
2. Humerus with medial ridge on the proximocaudal face.
3. Humerus with rectangular radial condyle.

*Baurutitan britoi* (Kellner et al., 2005)

1. Strongly pointed laterally directed process intercepting the spinoprezygapophyseal lamina on the caudal 1.
2. Anterolaterally directed spinoprezygapophyseal lamina of anterior caudal vertebrae.

*Maxakalisaurus topai* (Kellner et al., 2006)

1. Tail composed of anterior and mid-posterior caudal vertebrae with the anterior (and posterior) surface of the centrum dorsoventrally compressed.
2. Mid-posterior caudal vertebrae with the lateral surface of the centrum strongly concave (spool-shaped).
3. Dorsal margin of neural spine in mid-posterior caudal vertebrae inclined anteriorly.
4. Presence of at least one mid-posterior caudal with biconvex centrum.
5. Metacarpal IV about 12% shorter than metacarpal II.
6. Sacral centrum with keel-shaped ventral surface.

*Gondwanatitan faustoi* (Kellner and de Azevedo, 1999)

1. Deltopectoral crest of humerus very well developed and curved medially.
2. Tibia with anterior part of the proximal articulation projecting dorsally.
3. Cnemial crest only slightly curved laterally.

*Muylenesaurus pecheni* (Calvo et al., 2007a)

1. Basal tubera diverge 70 degree from each other.
2. Extensive, thin and concave medial lamina that unit basal tubera ventrally.
3. Basioccipital condyle wider than the proximal portion of the basal tubera.
4. Posterior dorsal neural spines with large prespinal lamina reinforced by two small accessory laminae.
5. Distal end of pubic blade rectangular and medially thick.

*Overosaurus paradasorum* (Coria et al., 2013)

1. Posterior cervical vertebrae with long pre- and postzygapophyses that project beyond the anterior and posterior borders of the centrum, respectively.
2. Postspinal lamina absent in all dorsal neural spines.
3. Wide and massive 9th and 10th caudal centra that are slightly excavated laterally and have relatively flat ventral surfaces.
4. Ilium proportionally shorter anteroposteriorly and taller dorsoventrally (than in other lithostrotians).
5. Preacetabular process of the ilium strongly deflected laterally and with a ventrolaterally tapering end.

*Epachthosaurus sciuttoi* (Powell, 1990; Martinez et al., 2004)

1. Middle and caudal dorsal vertebrae exhibiting accessory articular processes extending ventrolaterally from the hyposphene.
2. Strongly developed intraprezygapophyseal lamina of dorsal vertebrae.
3. Aliform processes projecting laterally from the dorsal portion of the spinodiapophyseal lamina of dorsal vertebrae.
4. Hyposphene-hypantrum articulations in caudals 1-14.
5. 2-2-3-2-0 pedal phalangeal formula.

*Panamericansaurus schroederi* (Porfiri and Calvo 2010)

1. Subsequent middle caudal vertebrae with anterodorsally inclined prezygapophysis equal or shorter than the length of the base of the vertebral body.
2. Postzygapophyses medially located with respect to the vertebral body of caudal vertebrae.
3. Spinoprezygapophyseal lamina dorsoventrally expanded in middle caudal vertebrae.
4. Humerus with an index of less than 0.40 robustness.

*Rinconsaurus caudamirus* (Calvo and Gonzalez Riga, 2003)

1. Mid-anterior dorsal vertebral neural spines inclined posteriorly more than 60 degrees with respect to the vertical.
2. Procoelous posterior caudal vertebral centra with the eventual intercalation of a series of amphicoelous-biconvex or amphicoelous-opisthocoelous-biconvex centra.

*Aeolosaurus maximus* (Santucci and de Arruda-Campos, 2011)

1. Well-developed posterior protuberance below the articular area on the anterior and middle haemal arches.
2. Lateral bulge on the distal portion of the articular process of the mid-posterior haemal arches.

*Paludititan nalatzensis* (Csiki et al., 2010)

1. Dorsal segment of the anterior centrodiapophyseal lamina curves anterodorsally and extends parallel to the dorsal segment of the posterior centrodiapophyseal lamina on dorsal vertebrae.
2. Anterior and anteriormost mid-caudal neural spines short, upright and with anteriorly projecting anterodorsal corner.
3. Presence of amphiplatyan and platycoelous caudal vertebrae intercalated between procoelous caudal vertebrae in the mid-section of the tail.
4. Presence of a posterolateral buttress on the iliac peduncle of the ischium.

*Trigonosaurus pricei* (Campos et al., 2005)

1. Elongated midcervical vertebrae, with low neural spine and concave ventral margin.
2. Elongated middorsal vertebrae with strongly posteriorly inclined neural spine.
3. Dorsal vertebrae 9 and 10 with incipient postzygodiapophyseal lamina.
4. Anterior caudal vertebrae with thin base broadening towards the top.
5. Anteriormost caudals (2-5), with two and middle caudals with one pronounced dorsal depression on the transverse process.
6. Articulation surfaces for haemal arches strongly developed starting on caudal 3 until the last preserved element (caudal 20).
7. Transverse processes well developed throughout the sequence formed by anterior and medial caudals (until at least caudal 20).

*Argentinosaurus huinculensis* (Bonaparte and Coria, 1993)

1. Hypantrum-hyposphene in dorsal vertebrae with accessory articulations.

*Argyrosaurus superbus* (Lydekker, 1893; Mannion and Otero, 2012)

1. Medial margin of humerus forms a transversely wide ridge that projects prominently anteriorly.
2. Midshaft of humerus extremely compressed anteroposteriorly (transverse to anteroposterior width ratio = 2.6).
3. Transverse width of distal end of radius only slightly greater than midshaft width (ratio = 1.3).
4. Radius is subtriangular in distal end view.
5. Extreme elongation of metacarpals (longest metacarpal to radius length ratio = 0.6).

*Futalognkosaurus dukei* (Calvo et al., 2007b)

1. Neurapophyses of the atlas laminar and quadrangular, posteriorly directed.
2. Neural spine of the axis high, triangular.
3. Posterior border of the neural spine on middle cervical elements concave.
4. Ventral depression between parapophyses on middle cervical centra.
5. Anterior dorsal vertebrae with horizontal and aliform diapophysis.
6. Pre- and postzygapophyses of anterior dorsal vertebrae horizontal.
7. First caudal vertebra with prespinal lamina bifurcated on its base forming two small infraprespinal laminae.
8. Supraspinal cavity in first caudal vertebra bordered by the prespinal and lateral laminae.
9. Second and third sacral ribs fused.

*Mendozasaurus neguyelap* (Gonzalez Riga, 2003)

1. Two subtriangular infrapostzygapophyseal fossae in anterior dorsal vertebrae.
2. “Postzygapostspinal” laminae parallel to postzygapophyseal facets in anterior dorsal vertebrae.
3. Interzygapophyseal cavity dorsoventrally extended and limited by the spinopostzygapophyseal and spinoprezygapophyseal laminae in anterior caudal vertebrae.
4. Middle caudal centra slightly procoelous with dorsally displaced condyles.
5. Laminar mid-posterior caudal neural spines with horizontal and straight dorsal border, and anterodorsal corner shaped at a right angle.
6. Large subconical osteoderms, lacking cingulum.

*Chubutisaurus insignis* (del Corro, 1975; Carballido et al., 2011)

1. Anterior dorsal vertebrae with a medial centroprezygapophyseal lamina that connects the medial part of the centroprezygapophyseal lamina with the ventral half of the intraprezygapophyseal lamina, forming the ventromedial edge of the associated subrectangular fossa.
2. Anterior dorsal vertebrae with a stout and internally pneumatized medial pillar between the neural canal and the ventral edge of the intraprezygapophyseal lamina.
3. Middle dorsal vertebrae with large and deep pleurocoels that present three inner laminae.

*Lirainosaurus asitbiae* (Sanz et al., 1999; Diaz et al., 2011, 2012, 2013a, b)

1. Presence of a foramen in the distal surface of each basal tubera.
2. The absence of median subcondylar foramina in the basioccipital, below the occipital condyle and between the basal tubera.
3. Presence of a lamina in the interzygapophyseal fossa in the proximal caudal vertebrae (podl that separates pocdf and posdf).
4. Spinopostzygapophyseal structure not posteriorly projected in the posterior caudal vertebrae.
5. Sternal plate with an anteroventral ridge, an anterolateral process and a concave lateral edge.

*Ligabuesaurus leanzai* (Bonaparte et al., 2006; D’Emic, 2012)

1. Distal scapular blade with rounded dorsal expansion.
2. Fossae on proximoventral faces of metatarsals II and III.
3. Deep pit on ventrodistal face of manual phalanx I.1.

*Pellegrinisaurus powelli* (Salgado, 1996)

1. Midposterior and posterior caudal vertebrae with anteriorly heightened neural spines.
2. Dorsal centra strongly depressed in height.

*Dreadnoughtus schrani* (Lacovara et al., 2014)

1. Ventral keel on first caudal vertebra.
2. Anterior caudal neural spines with subdivided pneumatocoel between spinoprezygapophyseal and -postzygapophyseal laminae.
3. Middle caudal neural spines with triangular anterodorsal processes that extends past anterior margin of the centrum.
4. Anterior haemal arches with paddle shape distal expansion.
5. Posterodorsally-anteroventrally oriented ridge on medial surface of the anterior portion of the scapular blade.
6. Posteromedial surface of the radius concave in proximal view.
7. Nearly square distal end of the radius.

*Normanniasaurus genceyi* (Le Loeuff et al., 2013).

1. Anterior caudal vertebra with ‘antepostzygapophysial’ foramen.
2. Amphicoelous middle caudal vertebra with cranially inserted neural arch.

*Nemegtosaurus mongoliensis* (Nowinski, 1971; Wilson, 2005)

1. Presence of a spur on the posterior squamosal.
2. Conspicuous fossa surrounding the preantorbital fenestra.
3. Accessory fenestra positioned anterodorsal to preantorbital fenestra.
4. Jugal foramen.
5. Coronoid foramen.

*Wintonotitan wattsi* (Hocknull et al., 2009; Poropat et al., 2014)

1. Middle–posterior dorsal neural spine summit with rounded median ridge linking pre- and postspinal laminae.
2. Anterior and anterior–middle caudal centra with a horizontal ridge at approximately mid-height which projects as far laterally as the lateral margins of the anterior and posterior articular surfaces of the centrum.
3. Anterior chevrons with proximal articular ends that are, in lateral view, narrower anteroposteriorly than are the proximal rami themselves at about mid-height of the haemal canal.
4. Scapular blade with fossa on medial surface close to the acromion–distal blade junction.
5. Radius proximal end subcircular with medially directed projection.
6. Metacarpus with deep fossa on proximal surface, at the intersections of metacarpals I, II and III.
7. Metacarpal III with distal end more expanded transversely than that of the proximal end.
8. Metacarpal IV with medially projecting bulge on the dorsal surface, close to shaft mid-length.

*Tastavinsaurus sanzi* (Canudo et al., 2008; D’Emic, 2012)

1. Distal neural spines of dorsal vertebrae with small fossae and foramina.
2. Distal neural spines of dorsal vertebrae with upwardly directed hook-like processes.
3. Sacrum narrow.
4. Sacricostal yoke projects well below ventral margin of sacral centra.
5. Fifth sacral rib dorsoventrally deep, flaring distally.
6. Metatarsal I with ventrally expanded distal condyles.
7. Metatarsal I without ventrally expanded proximal articular surface.
8. Metatarsals II–IV divided distally.
9. Metatarsal IV with divided distal articular surface.
10. Metatarsal V with proximoventral flange.
11. Pedal phalanx I.1 subrectangular.

*Malarguesaurus florenciae* (Gonzalez Riga et al., 2009)

1. Proximal caudal neural spines vertically directed, with a concave caudal border and a caudodorsal corner forming a right angle
2. Procoelous- opisthoplatyan proximal and middle caudal vertebrae associated with procoelous distal caudal centra;
3. Distal caudal neural spines having a concave and depressed dorsal border.

*Ampelosaurus atacis* (Le Loeuff, 1995; Le Loeuff, 2005)

1. Slightly spatulate teeth

*Bonitasaura salgadoi* (Gallina, 2011; Gallina and Apesteguia, 2011; Gallina and Apesteguia, 2015)

1. Thin and enlarged maxillary process of lacrimal oriented downward and forward.
2. Posterior dentary edentulous, with sharp dorsal edge, and highly vascularized lateral surface.
3. Ventral longitudinal keel in posterior half of axial centrum.
4. Tongue-like process on spinoprezygapophyseal laminae of mid-cervical vertebrae.
5. Circular, vertically oriented fossae aligned with anterior median lamina in mid-dorsal vertebrae.
6. Anterior, longitudinal ridge of the tibia with marked promontory just over anterior process of distal end.

*Daxiatitan binglingi* (You et al., 2008; D’Emic, 2012)

1. Femoral distal condyles bevelled 10° dorsolaterally with respect to shaft.

*Karongasaurus gittelmani* (Gomani, 2005)

1. Dorsal margin of lateral surface of dentary flares outward anteriorly.

*Atsinganosaurus velauciensis* (Garcia et al., 2010)

Garcia et al., 2010 does not explicitly state any autapomorphies but rather a unique combination of traits expressed within this titanosaurian.

*Lohuecotitan pandafilandi* (Diaz Diez et al., 2016)

1. Short postspinal lamina with a transversely expanded distal end represented by smooth scars in the dorsal vertebrae.
2. Anteriormost caudal vertebrae with the medial spinoprezygapophyseal lamina and medial spinopostzygapophyseal lamina ventrally connected with the prespinal and postspinal laminae, respectively.
3. Anterior neural spines on caudal vertebrae with a dorsal projection of the prespinal and postspinal laminae, resulting V-shaped outline.
4. Anterior neural spines on caudal vertebrae bear a greek-cross-like cross-section.
5. Middle caudal vertebrae having two rough structures in the dorsal edge of the posterior articulation, which extends to the dorsal surface of the centrum.
6. The articular ends of the rami of the posterior haemal arches are fully divided in two articular facets.

*Shingopana songwensis* (Gorscak et al., 2017)

1. Middle-posterior cervical vertebra with divided spinoprezygapophyseal lamina, forming a spinoprezygapophyseal lamina fossa, near the base of an expanded neural spine.

*Mansourasaurus shahinae* (Sallam et al., 2017)

1. Dentary with ten tooth positions.
2. Symphyseal region of dentary with pronounced ventral projection (‘chin’) that comprises nearly one-third dorsoventral depth of anterior end of bone.
3. Meckelian groove faces predominantly ventrally along dentary.
4. Capitulotubercular web of anterior cervical rib pierced by foramen.
5. Anterior to middle cervical vertebral centrum with elliptical foramen in posterior portion of lateral surface.
6. Anteroposterior length of parapophysis in anterior to middle cervical vertebra nearly equal to that of centrum.
7. Ratio of mediolateral to anteroposterior dimensions of distal radius roughly 4.0.

*Mnyamawamtuka moyowamkia* (Current study)

1. Middle and posterior dorsal vertebrae with vertical lamina between neural canal and interprezygapophyseal lamina that bifurcates dorsally.
2. Posterior dorsal vertebra with no interpostzygapophyseal lamina as the postspinal lamina continues to the dorsal margin of the neural canal.
3. Prominent dorsolateral expansion on the posterior centrum of the middle caudal vertebra.
4. Curved crest with accompanying fossa within the dorsomedial region of the proximal scapular blade.
5. Sternal plate unusually small estimated to be, at most, 42% of humerus length.

*Notocolossus gonzalezparejasi* (Gonzalez Riga et al., 2016)

1. Anterior dorsal vertebra with parapophyseal centrodiapophyseal fossa subdivided by two accessory laminae (one subvertical and visible in anterior and lateral views, the other anterodorsally oriented and visible only in lateral view).
2. Anterior caudal vertebrae with laminae that converge ventrally on the anterior surface of the neural spine, not reaching the prezygapophyses and forming a V-shaped conformation in anterior view.
3. Humerus with greatly expanded proximomedial process, the proximal apex of which lies well medial to the humeral midshaft.
4. Proportionally wide proximal end of humerus (proximal width: midshaft width ≈ 2.9).
5. Proximolaterally–distomedially oriented ridge bounding distal edge of coracobrachialis fossa of the humerus.
6. Pes with metatarsal I with proximal dorsoventral diameter greater than the proximodistal length of the bone.
7. Relatively short metatarsal III (only 1.2 times the length of metatarsal I).
8. Proximal phalanges more than half as wide as their corresponding metatarsals are long.
9. Pedal unguals reduced, rugose, and distally truncated.

*Patagotitan mayorum* (Carballido et al., 2017)

1. Anterior dorsal vertebrae (D1 – D3) with vertical prezygodiapophyseal lamina, due to the elevated position of the prezygapophysis respect to the diapophysis.
2. Anteriormost dorsal vertebrae (D1 – D2) with ventral bulge in prespinal lamina.
3. Hyposphene-hypantrum restricted to the articulation between D3 and D4.
4. First caudal vertebra with flat anterior and convex posterior articular surfaces.
5. Anterior caudal vertebrae with neural spines four to six times mediolaterally wider than anteroposteriorly long.
6. Anterior caudal neural spines incipiently bifid with anteriorly directed tips.
7. Straight lateral edge on distal femur.

3. Taxon/Character Modifications

New Characters: 152, 155, 180. 194, 265, 266, 269, 270, 271 (in bold above, S2)

Modified scorings: Character number (previous scoring – new scoring)

*Malawisaurus dixeyi*

53 (1-?); 151 (?-0); 217 (?-0); 218 (?-1); 219 (?-1)

*Mendozasaurus neguyelap*

170 (0-1)

*Rapetosaurus krausei*

146 (0-1); 223 (?-0); 224 (?-0)

*Mansourasaurus shahinae*

181 (?-1); 224 (1-0)

*Phuwiangosaurus sirindhornae*

263 (0-?); 264 (1-?)

*Tangvayosaurus hoffeti*

263 (0-?); 264 (1-?)

*Bonitasaura salgadoi*

263 (0-?); 264 (1-?)

4. References

Allain, R., P. Taquet, B. Battail, J. Dejax, P. Richir, M. Véran, F. Limon–Duparcmeur, R. Vacant, O. Mateus, P. Sayarath, B. Khenthavong, and S. Phouyavong. 1999. Un nouveau genre de dinosaure sauropode de la formation des Grès supérieurs (Aptien–Albien) du Laos. Comptes Rendus De l'Académie Des Sciences – Series IIA – Earth and Planetary Science 329:609–616.

Apesteguía, Sebastián. 2004. *Bonitasaura salgadoi* gen. et sp. nov.: a beaked sauropod from the Late Cretaceous of Patagonia. Naturwissenschaften 91:493–497.

Bonaparte, J. F., and J. E. Powell. 1980. A continental assemblage of tetrapods from the Upper Cretaceous beds of El Brete, northwestern Argentina (Sauropoda–Coelurosauria–Carnosauria– Aves). Mémoires de le Société Géologique de France (Nouvelle Série) 139:19–28.

Bonaparte, J. F., and R. A. Coria. 1993. Un nuevo y gigantesco saurópodo titanosaurio de la Formación Río Limay (Albiano–Cenomaniano) de la Provincia del Neuquén, Argentina. Ameghiniana 30:271–282.

Bonaparte, J. F., B. J. González Riga, and S. Apesteguía. 2006. *Ligabuesaurus leanzai* gen. et sp. nov.(Dinosauria, Sauropoda), a new titanosaur from the Lohan Cura Formation (Aptian, Lower Cretaceous) of Neuquén, Patagonia, Argentina. Cretaceous Research 27:364–376.

Borsuk–Bialynicka M. 1977. A new camarasaurid sauropod *Opisthocoelicaudia skarzynskii*, gen. n., sp. n. from the Upper Cretaceous of Mongolia. Palaeontologia Polonica 37:5–64.

Calvo, J. O., and J. F. Bonaparte. 1991. *Andesaurus delgadoi*, gen. et sp. nov. (Saurischia–Sauropoda), dinosaurio Titanosauridae de la Formacíon Río Limay (Albiano–Cenomaniano), Neuquén, Argentina. Ameghiniana 28:303–310.

Calvo, J. O., and B. J, Gonzaléz Riga. 2003. *Rinconsaurus caudamirus* gen. et sp. nov., a new titanosaurid (Dinosauria, Sauropoda) from the Late Cretaceous of Patagonia, Argentina. Revista Geologica de Chile 30:333–353.

Calvo, J. O., and J. D. Porfiri. 2010. *Panamericansaurus* *schroederi* gen. nov. sp. nov. Un nuevo Sauropoda (Titanosauridae-Aeolosaurini) de la Provincia del Neuquén, Cretácico Superior de Patagonia, Argentina. Brazilian Geographical Journal: Geosciences and Humanities Research Medium 1:100–115.

Calvo, J. O., B. J. González Riga, and J. D. Porfiri. 2007a. A new titanosaur sauropod from the Late Cretaceous of Neuquén, Patagonia, Argentina. Arquivos Do Museu Nacional 65:485–504.

Calvo, J. O., J. D. Porfiri, B. J. González Riga, and A. W. A. Kellner. 2007b. A new Cretaceous terrestrial ecosystem from Gondwana with the description of a new sauropod dinosaur. Anais Da Academia Brasileira De Ciencias. 79:529–541.

Calvo, J. O., J. D. Porfiri, B. J. González Riga, and A. W. Kellner. 2007c. Anatomy of *Futalognkosaurus dukei* Calvo, Porfiri, González Riga & Kellner, 2007 (Dinosauria, Titanosauridae) from the Neuquén Group (Late Cretaceous), Patagonia, Argentina. Arquivos Do Museu Nacional, Rio De Janeiro 65:511–526.

Canudo, J. I., R. Royo-Torres, and G. Cuenca-Bescos. 2008. A new sauropod: *Tastavinsaurus* *sanzi* gen. et sp nov from the Early Cretaceous (Aptian) of Spain. Journal of Vertebrate Paleontology 28:712–731.

Campos, D. A., A. W. A. Kellner, R. J. Bertini, and R. M. Santucci. 2005. On a titanosaurid (Dinosauria, Sauropoda) vertebral column from the Bauru group, Late Cretaceous of Brazil. Arquivos Do Museu Nacional 63:565–593.

Carballido, J. L., D. Pol, I. Cerda, and L. Salgado. 2011. The osteology of *Chubutisaurus insignis* del Corro, 1975 (Dinosauria: Neosauropoda) from the “middle” Cretaceous of Central Patagonia, Argentina. Journal of Vertebrate Paleontology 31:93–110.

Carballido, J. L., D. Pol, A. Otero, I. A. Cerda, L. Salgado, A. C. Garrido, J. Ramezani, N. R. Cúneo, and J. M. Krause. 2017. A new giant titanosaur sheds light on body mass evolution among sauropod dinosaurs. Proceedings: Biological Sciences 284:20171219–10.

Csiki, Z., V. Codrea, C. Jipa–Murzea, and P. Godefroit. 2010. A partial titanosaur (Sauropoda, Dinosauria) skeleton from the Maastrichtian of Nalat–Vad, Hateg Basin, Romania. Neues Jahrbuch Fur Geologie Und Palaontologie–Abhandlungen 258:297–324.

Cope, E. D. 1877. On a gigantic saurian from the Dakota epoch of Colorado. Paleontological Bulletin 25:5–10.

Coria, R. A., L. S. Filippi, L. M. Chiappe, R. Garcia, and A. B. Arcucci. 2013. *Overosaurus paradasorum* gen. et sp. nov., a new sauropod dinosaur (Titanosauria: Lithostrotia) from the Late Cretaceous of Neuquén, Patagonia, Argentina. Zootaxa 3683:357–376.

Curry Rogers, K. A, and C. A. Forster. 2001. The last of the dinosaur titans: a new sauropod from Madagascar. Nature 412:530–534.

Curry Rogers, K, and C. A. Forster. 2004. The skull of *Rapetosaurus* *krausei* (Sauropoda: Titanosauria) from the Late Cretaceous of Madagascar. Journal of Vertebrate Paleontology 24:121–144.

Curry Rogers, K. A. 2005. Titanosauria: a phylogenetic overview; pp. 50–103 in K. A. Curry Rogers and J. A. Wilson (eds.), The Sauropods: Evolution and Paleobiology. University of California Press, Berkeley.

Curry Rogers, K. A. 2009. The postcranial osteology of *Rapetosaurus krausei* (Sauropoda: Titanosauria) from the Late Cretaceous of Madagascar. Journal of Vertebrate Paleontology 29:1046–1086.

D’Emic, M. D. 2012. The early evolution of titanosauriform sauropod dinosaurs. Zoological Journal of the Linnean Society 166:624–671.

D'Emic, M. D., and A. W. Jeffrey. 2011. New remains attributable to the holotype of the sauropod dinosaur *Neuquensaurus australis*, with implications for saltasaurine systematics. Acta Palaeontologica Polonica 56:61–73.

del Corro, G. 1975. Un nuevo sauropodo del Cretacico *Chubutisaurus insignis* gen. et sp. nov. (Saurischia–Chubutisauridae nov.) del Cretacico Superior (Chubutiano), Chubut, Argentina, 12–16 August 1974; pp. 229–240 in Actas I Congreso Argentino de Paleontologıa y Bioestratigrafıa, Tucuman, Volume 2, Asociacion Paleontologica Argentina, Tucuman, Argentina.

Díez Díaz, V., X. Pereda Suberbiola, and J. Luis Sanz. 2011. Braincase anatomy of the titanosaurian sauropod *Lirainosaurus astibiae* from the Late Cretaceous of the Iberian Peninsula. Acta Palaeontologica Polonica 56:521–533.

Díez Díaz, V., X. P. Suberbiola, and J. L. Sanz. 2012. Juvenile and adult teeth of the titanosaurian dinosaur *Lirainosaurus* (Sauropoda) from the Late Cretaceous of Iberia. Geobios 45:265–274.

Díez Díaz, V., X. Pereda Suberbiola, and J. L. Sanz. 2013a. The axial skeleton of the titanosaur *Lirainosaurus astibiae* (Dinosauria: Sauropoda) from the latest Cretaceous of Spain. Cretaceous Research 43:145–160.

Díez Díaz, V., X. P. Suberbiola, and J. L. Sanz. 2013b. Appendicular skeleton and dermal armour of the Late Cretaceous titanosaur *Lirainosaurus astibia* (Dinosauria: Sauropoda) from Spain. Palaeontologia Electronica 16:19A.

Díez Díaz, V., P. Mocho, A. Páramo, F. Escaso, F. Marcos-Fernández, J. L. Sanz, and F. Ortega. 2016. A new titanosaur (Dinosauria, Sauropoda) from the Upper Cretaceous of Lo Hueco (Cuenca, Spain). Cretaceous Research 68:49–60.

Gallina, P. A. 2011. Notes on the axial skeleton of the titanosaur *Bonitasaura* *salgadoi* (Dinosauria-Sauropoda). Anais Da Academia Brasileira De Ciencias. 83:235–246.

Gallina, P. A., and S. Apesteguía. 2011. Cranial anatomy and phylogenetic position of the titanosaurian sauropod *Bonitasaura* *salgadoi*. Acta Palaeontologica Polonica 56:45–60.

Gallina, P. A., and S. Apesteguía. 2015. Postcranial anatomy of *Bonitasaura salgadoi* (Sauropoda, Titanosauria) from the Late Cretaceous of Patagonia. Journal of Vertebrate Paleontology e924957.

Garcia, G., S. Amico, F. Fournier, E. Thouand, and X. Valentin. 2010. A new Titanosaur genus (Dinosauria, Sauropoda) from the Late Cretaceous of southern France and its paleobiogeographic implications. Bulletin De La Societe Geologique De France 181:269–277.

Gilmore, C. W. 1922. Discovery of a sauropod dinosaur from the Ojo Alamo Formation of New Mexico. Smithsonian Miscellaneous Collections 81:1–9.

Gilmore, C. W. 1946. Reptilian fauna of the North Horn Formation of central Utah. U.S. Geological Survey 210:1–52.

Gomani, E. M. 2005. Sauropod dinosaurs from the Early Cretaceous of Malawi, Africa. Palaeontologia Electronica 8:1.

González Riga, B. J. 2003. A new titanosaur (Dinosauria, Sauropoda) from the Upper Cretaceous of Mendoza province, Argentina. Ameghiniana 40:155–172.

González Riga, B. J. 2005. New fossil remains of *Mendozasaurus neguyelap* (Sauropoda, Titanosauria) from the Late Cretaceous of Mendoza, Argentina. Ameghiniana 42:535–548.

González Riga, B. J., M. C. Lamanna, L. D. Ortiz David, J. O. Calvo, and J. P. Coria. 2016. A gigantic new dinosaur from Argentina and the evolution of the sauropod hind foot. Nature Scientific Reports 6:19165.

González Riga, B. J., E. Previtera, and C. A. Pirrone. 2009. *Malarguesaurus florenciae* gen. et sp. nov., a new titanosauriform (Dinosauria, Sauropoda) from the Upper Cretaceous of Mendoza, Argentina. Cretaceous Research 30:135–148.

Gorscak, E., and P. M. O Connor. 2016. Time-calibrated models support congruency between Cretaceous continental rifting and titanosaurian evolutionary history. Biology Letters 12:20151047–5.

Gorscak, E., P. M. O'Connor, N. J. Stevens, and E. M. Roberts. 2014. The basal titanosaurian *Rukwatitan bisepultus* (Dinosauria, Sauropoda) from the middle Cretaceous Galula Formation, Rukwa Rift Basin, southwestern Tanzania. Journal of Vertebrate Paleontology 34:1133–1154.

Gorscak, E., P. M. O'Connor, E. M. Roberts, and N. J. Stevens. 2017. The second titanosaurian (Dinosauria: Sauropoda) from the middle Cretaceous Galula Formation, southwestern Tanzania, with remarks on African titanosaurian diversity. Journal of Vertebrate Paleontology 30:e1343250–23.

Gradstein, F. M., J. G. Ogg, and F. J. Hilgen. 2012. On the geologic time scale. Newsletters on Stratigraphy 45:171–188.

Haughton, S. H. 1928. On some reptilian remains from the dinosaur beds of Nyasaland. Transactions of the Royal Society of South Africa 16:67–75.

Hocknull S. A., M. A. White, T. R. Tischler, A. G. Cook, N. D. Calleja, T. Sloan, and D. A. Elliot. 2009. New mid–Cretaceous (latest Albian) dinosaurs from Winton, Queensland, Australia. PLoS ONE 4:e6190. doi:10.1371/journal.pone.0006190.

Jacobs, L., D. A. Winkler, W. R. Downs, and E. M. Gomani. 1993. New material of an Early Cretaceous titanosaurid sauropod dinosaur from Malawi. Palaeontology 36:523–534.

Jain, S. L., and S. Bandyopadhyay.1997. New titanosaurid (Dinosauria: Sauropoda) from the Late Cretaceous of central India. Journal of Vertebrate Paleontology 17:114–136.

Kellner, A. W. A., and S. A. K. Azevedo. 1999. A new sauropod dinosaur (Titanosauria) from the Late Cretaceous of Brazil. National Science Museum Monographs 15:111–142.

Kellner, A. W. A., D. A. Campos, and M. N. Trotta. 2005. Description of a titanosaurid caudal series from the Bauru Group, Late Cretaceous of Brazil. Arquivos Do Museu Nacional 63:529–564.

Kellner, A. W. A., D. A. Campos, S. A. K. Azevedo, M. N. F. Trotta, D. D. R. Henriques, M. M. T. Craik, and H. de P. Silva. 2006. On a new titanosaur sauropod from the Bauru Group, Late Cretaceous of Brazil. Boletim Do Museu Nacional 74:1–31.

Lacovara, K. J., M. C. Lamanna, L. M. Ibiricu, J. C. Poole, E. R. Schroeter, P. V. Ullmann, K. K. Voegele, Z. M. Boles, A. M. Carter, E. K. Fowler, V. M. Egerton, A. E. Moyer, C. L. Coughenour, J. P. Schein, J. D. Harris, R. D. Martínez, and F. E. Novas. 2014. A gigantic, exceptionally complete titanosaurian sauropod dinosaur from Southern Patagonia, Argentina. Scientific Reports 4:6196.

Le Loeuff, J., 1995. *Ampelosaurus atacis* (nov. gen., nov. sp.) un nouveau Titanosauridae (Dinosauria, Sauropoda) du Crétacé supérieur de la Haute Vallée de l’Aude (France). Comptes Rendus de l’Académie des Sciences Paris 321 (IIa), 693–699.

Le Loeuff, J., 2005. Osteology of *Ampelosaurus atacis* (Titanosauria) from Southern France. In: Tidwell, V., Carpenter, K. (Eds.), Thunder-Lizards. The Sauropodomorph Dinosaurs. Indiana University Press, Bloomington, pp. 115–137.

Le Loeuff, J., S. Suteethorn, and E. Buffetaut. 2013. A new sauropod dinosaur from the Albian of Le Havre (normandy, France). Oryctos 10:23–30.

Lydekker, R. 1893. The dinosaurs of Patagonia. Anales del Museo de la Plata, Sección de Paleontología 2:1–14.

Mannion, P. D. 2009. Review and analysis of African sauropodomorph dinosaur diversity. Palaeontologia Africana 44:108–111.

Mannion, P. D., and J. O. Calvo. 2011. Anatomy of the basal titanosaur (Dinosauria, Sauropoda) *Andesaurus delgadoi* from the mid–Cretaceous (Albian–early Cenomanian) Río Limay Formation, Neuquén Province, Argentina: implications for titanosaur systematics. Zoological Journal of the Linnean Society 163:155– 181.

Mannion, P. D., and A. Otero. 2012. A reappraisal of the Late Cretaceous Argentinean sauropod dinosaur *Argyrosaurus superbus*, with a description of a new titanosaur genus. Journal of Vertebrate Paleontology 32:614–638.

Mannion, P. D., P. Upchurch, R. N. Barnes, and O. Mateus. 2013. Osteology of the Late Jurassic Portuguese sauropod dinosaur *Lusotitan* *atalaiensis* (Macronaria) and the evolutionary history of basal titanosauriforms. Zoological Journal of the Linnean Society 168:98–206.

Martin, V., E. Buffetaut, and V. Suteethorn. 1994. A new genus of sauropod dinosaur from the Sao Khua Formation (Late Jurassic or Early Cretaceous) of northeastern Thailand. Comptes Rendus de l’Academie des Sciences de Paris 319:1085–1092.

Martínez, R., O. Gimenez, J. Rodriguez, M. Luna, and M. Lamanna. 2004. An articulated specimen of the basal titanosaurian (Dinosauria : Sauropoda) *Epachthosaurus sciuttoi* from the early Late Cretaceous Bajo Barreal Formation of Chubut Province, Argentina. Journal of Vertebrate Paleontology 24:107–120.

Mateus, O., L. L. Jacobs, A. S. Schulp, M. J. Polcyn, T. S. Tavares, A. Buta Neto, M. L. Morais, and M. T. Antunes. 2011. *Angolatitan adamastor*, a new sauropod dinosaur and the first record from Angola. Anais Da Academia Brasileira De Ciencias. 83:221–233.

McIntosh J. S. 1990. Sauropoda. In: Weishampel DB, Dodson P, Ósmolska H, eds. *The Dinosauria*. First edn. Berkeley, CA: University California Press, 345–401.

Nowinski, A. 1971. *Nemegtosaurus mongoliensis* n. gen., n. sp., (Sauropoda) from the uppermost Cretaceous of Mongolia. Palaeontologia Polonica 25:57–81.

Otero. 2010. The appendicular skeleton of *Neuquensaurus*, a Late Cretaceous saltasaurine sauropod from Patagonia, Argentina. Acta Palaeontologica Polonica 55:399.

Poropat, S.F., P. D. Mannion, P. Upchurch, S. A. Hocknull, B. P. Kear, and D. A. Elliott. 2015a. Reassessment of the non‐titanosaurian somphospondylan *Wintonotitan* *wattsi* (Dinosauria: Sauropoda: Titanosauriformes) from the mid‐Cretaceous Winton Formation, Queensland, Australia. Papers in Palaeontology 1:59–106.

Poropat, S.F., P. Upchurch, P. D. Mannion, S. A. Hocknull, B. P. Kear, T. Sloan, G. H. Sinapius, and D. A. Elliott. 2015b. Revision of the sauropod dinosaur *Diamantinasaurus* *matildae* Hocknull et al. 2009 from the mid-Cretaceous of Australia: implications for Gondwanan titanosauriform dispersal. Gondwana Research 27:995–1033.

Powell, J. E. 1986. Revisión de los titanosáuridos de América del Sur. Unpublished PhD dissertation, Universidad Nacional de Tucumán, Argentina, 340 pp.

Powell, J. E. 1990. *Epachthosaurus sciuttoi* (gen. et sp. nov.) un dinosaurio sauropodo del Cretácico de Patagonia (Provincia de Chu–but, Argentina). V Congreso Argentino de Paleontologia y Bioestratigrafia, Tucumán, Actas 1:123–128.

Powell J. E. 1992. Osteologia de *Saltasaurus* *loricatus* (Sauropoda-Titanosauridae) del Cretacico Superior del Noroeste Argentino. In: Sanz JL, Buscalioni AD, eds. Los dinosaurios y su entorno biotico. Cuenca: Instituto Juan de Valdes, Serie Actas Academicas 165–230.

Rauhut, O. W. M., K. Remes, R. Fechner, G. Cladera, and P. Puerta. 2005. Discovery of a short-necked sauropod dinosaur from the Late Jurassic period of Patagonia. Nature 435:670–672.

Riggs, E. S. 1903. *Brachiosaurus altithorax*, the largest known dinosaur. American Journal of Science (Series 4) 15:299–306.

Romer, A. S. 1956. Osteology of the reptiles. University of Chicago Press. Chicago, IL.

Royo-Torres, R., A. Cobos, L. Luque, A. Aberasturi, E. Espílez, I. Fierro, A. González, L. Mampel, and L. Alcalá. 2009. High European sauropod dinosaur diversity during Jurassic-Cretaceous transition in Riodeva (Teruel, Spain). Palaeontology 52:1009–1027.

Royo-Torres, R., L. Alcalá, and A. Cobos. 2012. A new specimen of the Cretaceous sauropod *Tastavinsaurus* *sanzi* from El Castellar (Teruel, Spain), and a phylogenetic analysis of the Laurasiformes. Cretaceous Research 34:61–83.

Salgado, L. 2003. Should we abandon the name Titanosauridae?: some comments on the taxonomy of titanosaurian sauropods (Dinosauria). Revista Española De Paleontología 18:15–21.

Salgado L., and Calvo J. O. 1992. Cranial osteology of *Amargasaurus* *cazaui* Salgado & Bonaparte (Sauropoda, Dicraeosauridae) from the Neocomian of Patagonia. Ameghiniana 29:337–346.

Salgado, L., and R. A. Garcia. 2002. Morphological variation in the sequence of caudal vertebrae of some titanosaur sauropods. Revista Española De Paleontología 17:211–216.

Salgado, L., R. A. Coria, and J. O. Calvo. 1997. Evolution of titanosaurid sauropods: Phylogenetic analysis based on the postcranial evidence. Ameghiniana 34:3–32.

Sallam, H. M., E. Gorscak, P. M. O'Connor, I. A. El-Dawoudi, S. El-Sayed, S. Saber, M. A. Kora, J. J. W. Sertich, E. R. Seiffert, and M. C. Lamanna. 2018. New Egyptian sauropod reveals Late Cretaceous dinosaur dispersal between Europe and Africa. Nature Ecology & Evolution 390:445–451.

Santucci, R. M., and A. C. de Arruda–Campos. 2011. A new sauropod (Macronaria, Titanosauria) from the Adamantina Formation, Bauru Group, Upper Cretaceous of Brazil and the phylogenetic relationships of Aeolosaurini. Zootaxa 3085:1–33.

Sanz, J. L., J. E. Powell, J. Le Loeuff, R. Martinez, and X. Pereda Suberbiola. 1999. Sauropod remains from the Upper Cretaceous of Laño (northcentral Spain). Titanosaur phylogenetic relationships. Estudios Del Museo De Ciencias Naturales De Alava 14:235–255.

Smith, J. B., M. C. Lamanna, K. J. Lacovara, P. Dodson, J. R. Smith, J. C. Poole, R. Giegengack, and Y. Attia. 2001. A giant sauropod dinosaur from an Upper Cretaceous mangrove deposit in Egypt. Science 292:1704–1706.

Suteethorn S., J. Le Loeuff, E. Buffetaut, V. Suteethorn, C. Talubmook, and C. Chonglakmani. 2009. A new skeleton of *Phuwiangosaurus sirindhornae* (Dinosauria, Sauropoda) from NE Thailand; pp. 189–215 in E. Buffetaut, G. Cuny, J. Le Loeff, and V. Suteethorn (eds.), Late Paleozoic and Mesozoic Ecosystems in Southeast Asia. Special Publication of the Geological Society of London 315.

Suteethorn, S., J. Le Loeuff, E. Buffetaut, and V. Suteethorn. 2010. Description of topotypes of *Phuwiangosaurus sirindhornae*, a sauropod from the Sao Khua Formation (Early Cretaceous) of Thailand, and their phylogenetic implications. Neues Jahrbuch für Geologie und Paläontologie, Abhandlungen, 256:109–121.

Tang, F., X. M. Kang, X. S. Jin, F. Wei, and W. T. Wu. 2001. A new sauropod dinosaur of Cretaceous from Jiangshan, Zhejiang Province. Vertebrata PalAsiatica 39:277–282.

Upchurch, P. 1995. The evolutionary history of sauropod dinosaurs. Philosophical Transactions: Biological Sciences 349:365–390.

Upchurch, P. 1998. The phylogenetic relationships of sauropod dinosaurs. Zoological Journal of the Linnean Society 124:43–103.

Upchurch, P., P. M. Barrett, and P. Dodson. 2004. Sauropoda; pp. 259–322 in D. B. Weishampel, P. Dodson, and H. Osmólska (eds.), The Dinosauria, second edition. University of California Press, Berkeley.

Wilson J. A. 2002. Sauropod dinosaur phylogeny: critique and cladistic analysis. Zoological Journal of the Linnean Society 136:217–276.

Wilson, J. A. 2005. Redescription of the Mongolian sauropod *Nemegtosaurus mongoliensis* Nowinski (Dinosauria: Saurischia) and comments on Late Cretaceous sauropod diversity. Journal of Systematic Palaeontology 3:283–318.

Wilson, J. A., and P. C. Sereno. 1998. Early evolution and higher-level phylogeny of sauropod dinosaurs. Society of Vertebrate Paleontology Memoir 5:1–68.

Wilson, J. A., and P. Upchurch. 2003. A revision of *Titanosaurus* Lydekker (Dinosauria–Sauropoda), the first dinosaur genus with a “Gondwanan” distribution. Journal of Systematic Palaeontology 1:125–160.

Wilson J. A., and P. Upchurch. 2009. Redescription and reassessment of the phylogenetic affinities of *Euhelopus zdanskyi* (Dinosauria: Sauropoda) from the Early Cretaceous of China. Journal of Systematic Paleontology 7:199–239.

Wiman, C. 1929. Die Kreide–Dinosaurier aus Shantung. Palaeontologia Sinica (Series C) 6:1–67.

You, H., D. Li, L. Zhou, and Q. Ji. 2008. *Daxiatitan binglingi*: a giant sauropod dinosaur from the Early Cretaceous of China. Gansu Geology 17:1–12.

Zaher H., D. Pol, A. B. Carvalho, P. M. Nascimento, C. Riccomini, P. Larson, R. Juárez– Valieri, R. Pires–Domingues, N. J. da Silva Jr., and D. de Almeida Campos. 2011. A complete skull of an Early Cretaceous sauropod and the evolution of advanced titanosaurians. PLoS ONE 6:e16663. doi:10.1371/journal.pone.0016663.
